# The effectiveness of different exercise mode interventions in improving disease activity in patients with ankylosing spondylitis: a network and dose-dependent meta-analysis

**DOI:** 10.3389/fphys.2025.1715944

**Published:** 2026-01-20

**Authors:** Guancheng Ye, Luyuan Gao, Chunping Liu, Jian Huang, Xiaojia Zheng, Yingkai Gao, Hao Wang, Hailong Wang

**Affiliations:** Department of Rheumatology, Dongzhimen Hospital, Beijing University of Chinese Medicine, Beijing, China

**Keywords:** ankylosing spondylitis, disease activity, dose-response relationship, exercise intervention, network meta-analysis, non-pharmacological therapy, physical function

## Abstract

**Background:**

Ankylosing spondylitis (AS) is a chronic inflammatory disease that impairs physical function, reduces quality of life, and is associated with psychological burdens such as anxiety and depression. While non-steroidal anti-inflammatory drugs (NSAIDs) and biologic therapies are standard treatments, exercise therapy is crucial for maintaining mobility and function. This study aimed to comprehensively compare the effects of 12 exercise interventions on AS patients’ disease activity and chest expansion (CE) via network meta-analysis (NMA) and dose-response meta-analysis, and explore dose-dependent effects to inform personalized exercise prescriptions.

**Methodology:**

Following the Preferred Reporting Items for Systematic Reviews and Meta-Analyses (PRISMA) guidelines, randomized controlled trials (RCTs) were searched from PubMed, Cochrane Library, Embase, and Web of Science until December 31, 2024. Eligible studies included adults with American College of Rheumatology/European League Against Rheumatism (ACR/EULAR)-diagnosed AS, comparing exercise with conventional treatment/placebo/no intervention, with outcomes of Bath Ankylosing Spondylitis Functional Index (BASFI), Bath Ankylosing Spondylitis Disease Activity Index (BASDAI), Bath Ankylosing Spondylitis Metrology Index (BASMI), and CE. Two reviewers screened literature, extracted data, and assessed bias using the Cochrane Handbook. NMA and dose-response analysis (expressed as metabolic equivalents of task (MET) minutes/week) were performed (Prospero: CRD420251001511).

**Results:**

Thirty-two RCTs with 1757 participants were included. NMA showed hippotherapy simulation (HS) was most effective for reducing BASFI; aerobic exercise (AE) + Pilates was superior for BASDAI and BASMI; AE + Stretching Exercise (SE)+Supervise best improved CE. Dose-response analysis revealed non-linear relationships, with specific effective dose ranges identified for each outcome. Subgroup and sensitivity analyses confirmed result robustness.

**Conclusion:**

Exercise interventions, especially HS, AE + Pilates, and AE + SE + Supervise, effectively improve AS patients’ disease activity and CE. Non-linear dose-response relationships emphasize personalized prescriptions, providing evidence-based guidance for integrating exercise into AS management, with future large-scale RCTs needed to validate dose effects.

## Introduction

AS is a chronic inflammatory disorder predominantly affecting the spine and sacroiliac joints (Sieper and Poddubnyy, 2017). Its prevalence is modulated by factors such as gender, geographic location, and socio-economic status, with data from a national registry study in Sweden indicating an incidence rate of approximately 0.18% (Exarchou et al., 2015). This condition not only results in diminished physical function and reduced quality of life for affected individuals but is also frequently associated with psychological challenges, including anxiety and depression (Meesters et al., 2014). Furthermore, an escalation in disease activity is closely linked to a heightened incidence of psychological issues (Sağ et al., 2018). Consequently, a holistic management approach that addresses both the physical and psychological dimensions is essential for enhancing the prognosis of patients with AS.

Currently, the management of AS emphasizes symptom control, inflammation reduction, and the prevention of structural damage. NSAIDs serve as the first-line therapeutic agents, offering relief from pain and stiffness; however, their long-term safety requires further investigation, and they may not be appropriate for all patients (Khan et al., 2012). For individuals exhibiting inadequate responses to NSAIDs, biologic therapies, particularly tumor necrosis factor (TNF) inhibitors, have emerged as the cornerstone of treatment, demonstrating efficacy in controlling disease activity (Betts et al., 2016). Beyond pharmacological interventions, non-pharmacological strategies, such as exercise therapy, play a crucial role in preserving the activity and function of patients with AS. These strategies can synergistically enhance the effects of pharmacotherapy, thereby further reducing disease activity and improving quality of life (Coksevim et al., 2018).

This study constitutes a NMA and dose-response meta-analysis grounded in RCTs, adhering to the PRISMA guidelines. The objective is to synthesize existing RCT evidence to evaluate the comparative effects of 12 exercise interventions on BASFI, BASDAI, BASMI, and CE in patients with AS. Additionally, the study seeks to investigate the dose-response relationship of each exercise, thereby providing a scientific foundation for the development of personalized, evidence-based exercise treatment plans in clinical practice. This research addresses a critical gap in the current literature concerning the comparative analysis of multiple exercise interventions and the optimization of exercise dosage.

## Materials and methods

### Search strategy

Two independent reviewers, YGC and GLY, executed a comprehensive literature search employing both automated and manual techniques across multiple databases, including PubMed, Cochrane Library, Embase, and Web of Science, to augment existing research. The search time is from the establishment of each database to December 31, 2024. We referred to previous meta-analyses of the same type to refine our retrieval strategy (Murphy et al., 2023; Migliorini et al., 2023). Initially, the titles and abstracts of the identified articles were screened to exclude irrelevant studies. Subsequently, the full text of the remaining articles was assessed to ascertain their eligibility based on the inclusion criteria. The complete retrieval strategy is detailed in Supplementary Method 1. This strategy was originally developed for the PubMed database and was subsequently adapted to accommodate the specific characteristics of other databases. The systematic searches are conducted independently by the two reviewers, YGC and GLY. Any disagreements are resolved through discussion between the reviewers or, if necessary, by involving a third senior reviewer, WHL. The study selection process adhered to the guidelines of the Preferred Reporting Items for Systematic Reviews and Meta-Analyses (PRISMA) (Page et al., 2021), and the meta-analysis protocol has been registered in Prospero (No.CRD420251001511).

### Inclusion and exclusion criteria

The inclusion criteria for the studies are as follows: (1) The study must be a randomized controlled trial; (2) Participants must be aged 18 years or older; (3) The diagnostic criteria for AS must adhere to the standards set by the ACR or the EULAR (Ward et al., 2019; Kiltz et al., 2009); (4) Outcome measures must include AS activity indices such as the BASFI, BASDAI, BASMI, and CE; (5) The treatment group must have received at least one form of exercise therapy, whereas the control group may have been administered routine medication, a placebo, or no intervention. The exclusion criteria are as follows: (1) Studies that contain duplicate articles or information; (2) Studies involving animal experiments; (3) Studies involving pregnant individuals or pediatric subjects; (4) Conference reports, letters, or case reports, as well as articles for which the full text is unavailable.

Before undertaking this study, a comprehensive review of high-quality meta-analyses pertinent to the subject was conducted to inform the classification of intervention measures. Following the selection of relevant literature, we systematically categorized the intervention measures examined in this study, ultimately identifying 12 distinct interventions. These include conventional treatment (CT), AE, yoga, qigong, resistance exercise (RE), AE + Physical Therapy (PT), AE + SE + Supervise, AE + SE, HS, SE, Pilates, and AE + Pilates. Detailed definitions and classifications of exercise therapy are provided in Supplementary Table S1.

### Data extraction and quality assessment

All relevant literature was imported into Endnote 20, and duplicates were systematically removed. Subsequently, two researchers (YGC and GLY) independently conducted a review of the titles and abstracts to identify studies pertinent to the research topic. Eligible studies were then downloaded in full and assessed by two independent reviewers, while data extraction was performed by another pair of independent reviewers (YGC and LCP). Any discrepancies encountered during this process were resolved through discussion with the reviewers or, if necessary, by consulting a third reviewer (WHL). The extracted information included: the name of the first author, age, gender, year of disease onset as reported in the article, research design, and outcome indicator values. The risk of bias in RCTs was evaluated using the RCT bias risk assessment tool recommended in the Cochrane Handbook version 5.1.0 (Higgins and Green, 2011). The research quality assessment covers seven core bias areas (random sequence generation, allocation concealment, participant and personnel blinding, outcome evaluation blinding, incomplete outcome data, selective reporting, and other biases). In instances where data were reported from multiple sources or the same RCT with varying follow-up periods, the results immediately following the intervention were selected as the experimental outcomes. In cases of missing data, the authors were contacted to obtain the original data. Our research primarily examines baseline data and immediate post-intervention data. We extracted the mean and standard deviation (SD) of changes from baseline, which serve as the principal data sources analyzed in this study. In instances where the standard deviation is not provided, alternative statistical measures such as the standard error, 95% confidence interval (CI), range, and quartiles are utilized for calculation (Wan et al., 2014). The specific formula employed for these calculations is detailed in Supplementary Method 2.

### Exercise dose calculation

The intensity of each exercise unit is determined by multiplying the intensity of a specific exercise pattern, measured in MET, by both the duration of a single exercise session per week and the frequency of exercise sessions per week. The resulting value is expressed in MET minutes per week. The specific intensity of each exercise was coded following the guidelines outlined in the 2011 Physical Activity Outline, which includes 821 activity-specific codes encompassing nearly all forms of exercise (Ainsworth et al., 2011). This standardized measurement approach aligns with the principle of rigorous data validation in systematic reviews and meta-analyses, as emphasized by Bergamin et al., who highlighted that uniform and validated measurement tools are critical for ensuring the reliability of aggregated data across multiple studies (Bergamin et al., 2023). The intensity of each exercise unit is determined by multiplying the intensity of a specific exercise pattern, measured in MET, by both the duration of a single exercise session per week and the frequency of exercise sessions per week. All measurements were validated using standardized protocols with test-retest reliability coefficients >0.80. Furthermore, we employed the calculation methodologies from analogous studies conducted in prior periods to determine the exercise dose, thereby ensuring the credibility of our results. Exercise frequency is quantified as the total number of exercise sessions per week, accounting for multiple sessions conducted within a single day. Furthermore, only the duration of the primary exercise intervention is considered when calculating exercise duration, excluding time allocated to warm-up and cool-down activities, as specified in the study. In cases where exercise duration is ambiguous, the average duration from all relevant studies on that specific exercise intervention is used. If the exercise duration progressively increases over several weeks, the average total exercise time is calculated.

### Statistical analysis

In this study, the mvmeta package was utilized within Stata versions 16.0 and 12.0 for conducting both network and conventional meta-analyses, employing the mean ± standard deviation as the effect measure for quantitative data analysis. The quality of the literature and the risk of bias were assessed using RevMan 5.3 software. Heatmaps were generated using R version 4.3.1, and the Metafor core package was downloaded specifically for meta-analysis purposes, providing the rma () function to fit either Random Effects or Fixed Effects Models. The code implemented a weighted least squares regression model using the rma () function, with dose (MET) as the independent variable and effect size (yi) as the dependent variable. Standardized Mean difference (SMD) was used to represent effect size, and a 95% confidence interval (CI) was employed to evaluate credibility. The node splitting method was applied to detect local inconsistencies, and clinical and methodological features were compared to assess the similarity of evidence networks. The results of comparisons between any two intervention measures were presented in the form of a league table. At a significance level of α = 0.05, the inconsistency test within the closed loop assesses the consistency between direct and indirect comparisons. An inconsistency factor (IF) with a 95% CI that includes 0 suggests consistency, whereas an IF greater than 1 indicates the presence of cyclic inconsistency, necessitating the use of traditionalpaired meta-analysis to quantify the sources of heterogeneity. Heterogeneity is quantified using the I^2^statistic, with I^2^values exceeding 50.00% indicating significant heterogeneity. When P ≥ 0.05 and I^2^ ≤ 50.00%, a fixed effects model is employed; otherwise, a random effects model is utilized. In cases where significant heterogeneity is detected, a random effects model is applied to summarize the effect sizes, and potential sources of heterogeneity are investigated through subgroup analysis and meta-regression. Additionally, the robustness of the results is evaluated by systematically excluding high-risk studies or subgroups with significant heterogeneity one at a time. The surface under the cumulative ranking curve (SUCRA) values, ranging from 0.00% to 100.00%, rank the intervention effects based on probability, with higher values indicating a greater likelihood of being the most effective treatment. To account for variations in rating scales across different indicators, the SMD and 95% CI were employed to compute the summary statistical data. For detailed explanations of the standardized mean deviation, confidence interval, and other pertinent concepts, please consult Supplementary Table S2. Sample size adequacy was assessed using power calculations for network meta-analysis, with minimum effect sizes of SMD = 0.30 considered clinically meaningful. Power analysis indicated adequate sample size for detecting moderate effect sizes (1-β = 0.80, α = 0.05).

The multi-arm experiment entails the merging of subgroups during data processing and analysis, drawing on the dual-arm experiment, or alternatively, the division and analysis based on distinct intervention measures. For multi-arm trials, subgroups were merged if they shared the same intervention to avoid double-counting. Data from multi-arm studies were split into independent comparisons when analyzing different interventions. Specifically, subgroups characterized by identical types of intervention measures, such as those involving dietary supplements with the same active ingredients, were combined for analysis. This approach was employed to enhance sample size and improve statistical power. All measurement data are presented in the form of mean and standard deviation, and all data units are standardized. For quantitative data, including uric acid levels and lipid metabolism indicators, the combined mean and difference were computed. The combined mean was determined using a weighted average, where the weights were assigned based on the sample size of each subgroup. This approach ensures that subgroups with larger sample sizes exert a greater influence on the overall results. Data from multi-arm studies were split into independent comparisons when analyzing different interventions.

## Results

### Study selection

**FIGURE 1:**
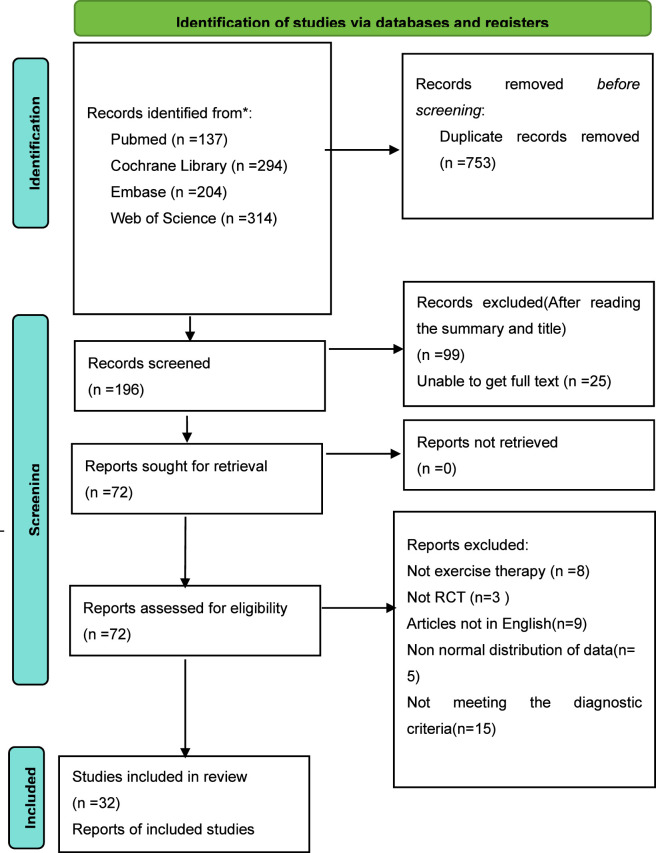
Document screening process and results.

Figure 1 illustrates the literature screening process. An initial total of 949 studies were retrieved from the database. Duplicate articles and studies not meeting the inclusion criteria were excluded. Following a thorough review of the texts, 32 articles satisfied the inclusion criteria and were incorporated into the meta-analysis. All articles were published in English and encompassed a total of 1,757 participants. The publication years of these studies span from 2002 to 2024. All 32 studies incorporated in the analysis were RCTs. A total of 12 intervention modalities were examined, including CT, AE, qigong, AE + SE, AE + SE + Supervise, SE, HS, AE + PT, RE, yoga, Pilates, and AE + Pilates. The fundamental characteristics of the included studies are presented in Supplementary Table S3, while detailed descriptions of the exercise intervention measures across all included literature are provided in Supplementary Table S4.

### Literature quality evaluation

**FIGURE 2:**
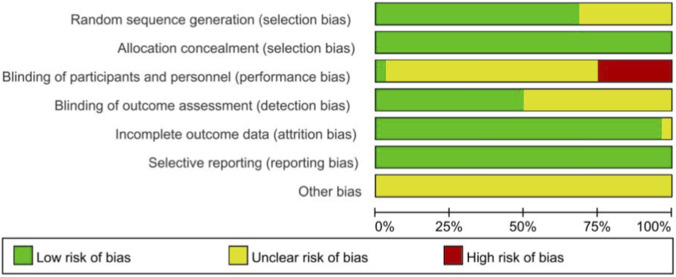
Risk of bias assessment included in the study.

We performed a quality assessment of the literature, focusing on 32 RCTs. This assessment was independently conducted by two researchers (YGC and LCP), with any disagreements resolved through consultation with a third researcher (WHL). Regarding random allocation methods, 22 articles were deemed low-risk as they employed either the random number table method or the computer-generated random allocation sequence (Acar et al., 2023a; Acar et al., 2023b; Aksoy et al., 2017; Alta et al., 2006; Altan et al., 2012; Analay et al., 2003; Aydın et al., 2016; Cetina et al., 2020; Dundar et al., 2014; Gandomi et al., 2022; Gencer et al., 2025; Hsieh et al., 2014; Jennings et al., 2015; Karapolat et al., 2009; Kjeken et al., 2013; Masiero et al., 2014; Salbaş and Karahan, 2023; Singh et al., 2023; Soufivand et al., 2024; Souza et al., 2017; Wang et al., 2022; Xie et al., 2019). Ten articles mentioned randomness but did not specify the random allocation scheme, thus were classified as having an unclear risk (Ayhan et al., 2011; Calik et al., 2021; Ciprian et al., 2013; Gurcay et al., 2008; Ince et al., 2006; Karahan et al., 2016; Lee et al., 2008; Oksüz and Unal, 2023; Roşu et al., 2014; Sweeney et al., 2002). Regarding blinding methods, 24 studies did not mention allocation scheme concealment or blinding, resulting in an unclear risk classification (Acar et al., 2023a; Acar et al., 2023b; Aksoy et al., 2017; Alta et al., 2006; Altan et al., 2012; Analay et al., 2003; Aydın et al., 2016; Cetina et al., 2020; Gencer et al., 2025; Hsieh et al., 2014; Jennings et al., 2015; Karapolat et al., 2009; Masiero et al., 2014; Salbaş and Karahan, 2023; Ayhan et al., 2011; Calik et al., 2021; Ciprian et al., 2013; Gurcay et al., 2008; Ince et al., 2006; Karahan et al., 2016; Lee et al., 2008; Oksüz and Unal, 2023; Roşu et al., 2014; Sweeney et al., 2002), and 8 studies employed an open-label design, lacking blinding for both participants and evaluators, and were consequently classified as high-risk (Dundar et al., 2014; Gandomi et al., 2022; Kjeken et al., 2013; Singh et al., 2023; Soufivand et al., 2024; Souza et al., 2017; Wang et al., 2022; Xie et al., 2019). All 32 studies possessed complete datasets; however, one study did not specify whether all outcome measures were comprehensively reported and was consequently assessed as having an uncertain risk of bias (Xie et al., 2019). None of the studies exhibited selective reporting of research results, thereby being classified as low-risk. Nonetheless, other potential sources of bias remain indeterminate. Utilize RevMan 5.3 software to generate the illustration depicted in Figure 2. The risk of bias for each study is presented in Supplementary Figure S1.

### Network meta analysis results

#### Ankylosing spondylitis efficacy score

##### BASFI

**FIGURE 3:**
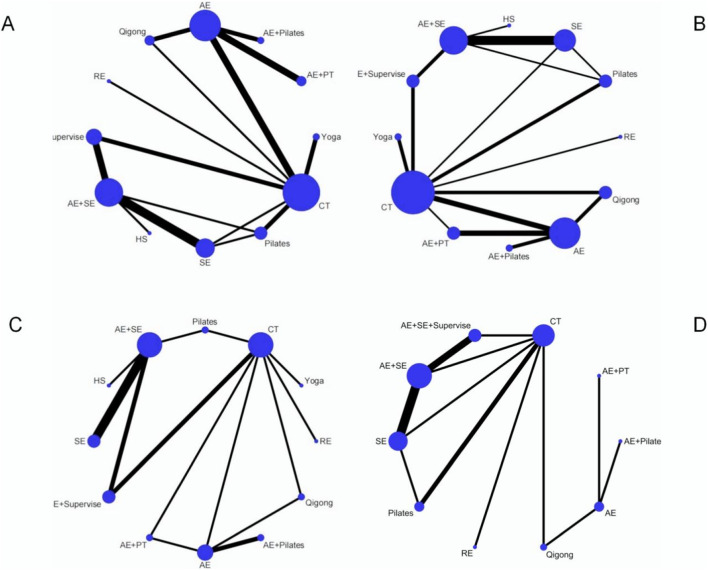
The evidence network of all papers on different treatments. **(A)** BASFI; **(B)** BASDAI; **(C)** BASMI; **(D)** CE. The thickness of the lines represents the number of studies, and the sizes of the nodes indicate the total sample sizes for each treatment.

**FIGURE 4:**
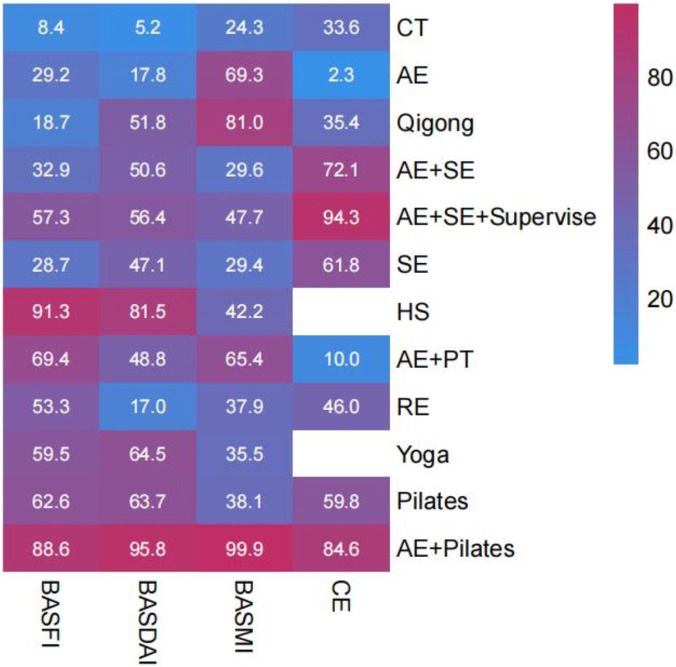
The SUCRA values of each treatment modality.

**FIGURE 5:**
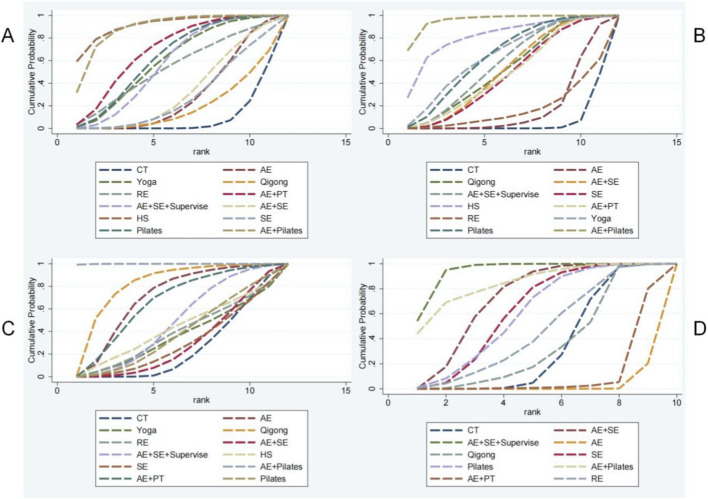
Ranking of the cumulative probabilities for core parameters. **(A)** BASFI; **(B)** BASDAI; **(C)** BASMI; **(D)** CE.

The investigation of BASFI encompassed 27 RCTs with a cumulative total of 1,503 patients, examining 11 distinct exercise interventions. The network relationships among these interventions are depicted in Figure 3A. Initially, each intervention was compared to conventional treatment through routine meta-analysis. The findings indicated that both Yoga (SMD = −0.47, 95% CI = −0.78 to −0.16; I^2^ = 39.90%, P < 0.01) and Pilates (SMD = −0.57, 95% CI = −1.01 to −0.12; I^2^ = 0.00%, P = 0.013) demonstrated statistically significant improvements, as illustrated in Supplementary Figure S2. Testing for inconsistency revealed a P-value greater than 0.05, thereby supporting the application of a consistency model for NMA, as shown in Supplementary Figure S3. The NMA further revealed that, compared to conventional treatment, interventions such as yoga (SMD = −0.49, 95% CI = −0.88 to −0.10), AE + PT (SMD = −0.60, 95% CI = −1.07 to −0.13), AE + SE + Supervise (SMD = −0.45, 95% CI = −0.82 to −0.08), HS (SMD = −1.08, 95% CI = −0.91 to −0.26), Pilates (SMD = −0.51, 95% CI = −0.93 to −0.10), and AE + Pilates combined with Pilates (SMD = −0.91, 95% CI = −1.45 to −0.38) exhibited significant advantages, with the differences being statistically significant. A pairwise comparison analysis indicates that both simulated equestrian exercise and a combination of AE + Pilates demonstrate superiority over AE alone, as well as over qigong and SE, with statistically significant differences (P < 0.05). Furthermore, HS is shown to be more effective than AE + SE, and AE + PT is more effective than AE alone, also with statistically significant differences (P < 0.05). No statistically significant differences (P > 0.05) were observed in other pairwise comparisons of exercise therapies. Detailed results of these comparisons are presented in Supplementary Table S5. According to the SUCRA analysis, simulated equestrian exercise, with a SUCRA value of 91.30%, emerges as the most effective intervention for reducing BASF scores. The SUCRA values for various interventions are illustrated in Figures 4, 5. Consequently, the findings from both routine and network meta-analyses are largely consistent. To assess potential local inconsistencies among the intervention measures, the node splitting method was employed. The results indicated P < 0.05, suggesting an absence of local inconsistency. In this study, we performed a consistency test on the closed loop formed by the intervention measures. Loop inconsistency, defined as the discrepancy between direct and indirect comparison results when different intervention measures create a closed loop in a NMA, can potentially impact the reliability of research conclusions and must be assessed using the IF. If the 95% confidence interval does not include 0, or if the IF value exceeds 1, it suggests the presence of loop inconsistency. The inconsistency test revealed that the combination of CT-AE + SE + Supervise-AE + SE−SE constitutes a closed loop (IF = 0.985, 95% CI [0.02, 1.95], P = 0.046), indicating a statistically significant difference between direct and indirect evidence, and thus inconsistency within the loop. In contrast, the inconsistency within other closed loops was relatively minor and not statistically significant, as presented in Table 1.

**TABLE 1:** Ring inconsistency detection results.

Outcome indicators	Closed loop	IF and 95%CI	P value
BASFI			
	CT-AE + SE + Supervise-AE + SE−SE	0.985 (0.02,1.95)	0.046
	AE + SE-SE-Pilates	0.474 (0,1.51)	0.37
	CT-AE + SE + Supervise-AE + SE-Pilates	0.295 (0,1.2)	0.521
	CT-AE-Qigong	0.228 (0,1.29)	0.674
	CT-SE-Pilates	0.216 (0,1.39)	0.719
BASDAI			
	CT-AE + SE-AE + SE + Supervise-SE	0.905 (0,2.27)	0.195
	AE + SE-SE-Pilates	0.825 (0,2.24)	0.254
	CT-AE-AE+PT	0.689 (0,1.93)	0.278
	CT-AE-Qigong	0.236 (0,1.25)	0.649
	CT-SE-Pilates	0.193 (0,1.42)	0.758
	CT-AE + SE-AE + SE + Supervise-Pilates	0.135 (0.1.2)	0.803
BASMI			
	CT-AE-Qigong	1.045 (0,2.15)	0.064
	CT-AE-HS	0.481 (0,1.54)	0.372
	CT-AE + SE-AE + SE + Supervise-Pilates	0.25 (0,1.33)	0.650
CE			
	CT-SE-Pilates	0.432 (0,1.64)	0.483
	CT-AE + SE-AE + SE + Supervise	0.244 (0,1.28)	0.643
	CT-AE+SE−SE	0.023 (0,1.14)	0.968

We employ conventional meta-analysis for direct pairwise comparisons to identify sources of heterogeneity. Although direct comparisons between CT and AE + SE + Supervise (I^2^ = 40.50%, p = 0.195), CT and SE (only one study), AE + SE + Supervise and AE + SE (I^2^ = 0%, p = 0.843), and AE + SE and SE (I^2^ = 0.00%, p = 0.647) did not indicate heterogeneity, the inconsistency test of the CT-AE + SE + Supervise-AE + SE−SE loops revealed inconsistencies, as illustrated in Supplementary Figures S4-6. This inconsistency may be attributed to the low accuracy of evidence for indirect comparisons within the loop, which arises from the absence of intervention measures that prevent direct comparisons. Consequently, the randomness in effect estimation introduces bias between the indirect path and the direct effect of the CT-SE comparison. It is recommended to prioritize direct evidence in pairwise comparisons while also considering the uncertainty associated with indirect pathways in clinical decision-making.

##### BASDAI

The investigation into the BASDAI encompassed 29 RCTs with a cumulative total of 1,585 patients, assessing 11 distinct exercise interventions. The network interconnections among these interventions are illustrated in Figure 3B. Initially, each intervention was compared to conventional treatment through routine meta-analysis. The findings indicated that AE (SMD = −0.41, 95% CI = −0.79 to −0.04; I^2^ = 0.00%, P = 0.032), Qigong (SMD = −0.57, 95% CI = −1.03 to −0.10; I^2^ = 0.00%, P = 0.016), AE + SE + Supervise (SMD = −0.84, 95% CI = −1.26 to −0.42], P < 0.01), yoga (SMD = −0.81, 95% CI = −1.13 to −0.49; I^2^ = 0.00%, P < 0.01), SE (SMD = −1.13, 95% CI = −1.97 to −0.30, P < 0.01), and Pilates (SMD = −0.91, 95% CI = −1.76 to −0.07, P = 0.034) demonstrated statistically significant improvements compared to conventional treatment, as depicted in Supplementary Figure S7. If the inconsistency model test yields a P > 0.05, the consistency model is deemed appropriate for conducting a NMA, as illustrated in Supplementary Figure S8. The results of the NMA indicate that, in comparison to conventional treatment, several interventions demonstrate statistically significant advantages: yoga (SMD = −0.78, 95% CI = [-1.30, −0.27]), AE + PT (SMD = −0.58, 95% CI = [-1.08, −0.09]), AE + SE + Supervise (SMD = −0.68, 95% CI = [-1.14, −0.22]), HS (SMD = −1.15, 95% CI = [-2.14, −0.16]), Pilates (SMD = −0.77, 95% CI = [-1.28, −0.26]), AE + Pilates (SMD = −1.51, 95% CI = [-2.20, −0.83]), Qigong (SMD = −0.63, 95% CI = [-1.09, −0.17]), AE + SE (SMD = −0.68, 95% CI = [-1.14, −0.22]), and SE (SMD = −0.59, 95% CI = [-1.14, −0.04]). All differences are statistically significant, with P < 0.05. Pairwise comparisons revealed that the combination of AE and Pilates therapy was significantly more effective than AE alone, qigong, SE AE + SE, AE + SE + Supervise, and RE, with statistically significant differences observed (P < 0.05). No statistically significant differences (P > 0.05) were found in pairwise comparisons among other exercise therapies. Consequently, the findings from both traditional and NMA are generally consistent, as detailed in Supplementary Table S6. According to the SUCRA analysis, AE + Pilates (95.8%) is likely the most effective intervention for reducing BASDAI scores. The SUCRA values for various intervention measures are presented in Figures 4, 5. The node-splitting method was employed to assess potential local inconsistencies among the intervention measures, with results indicating no local inconsistency (P < 0.05). A consistency test was also performed on the closed loop formed by the intervention measures in this study.

##### BASMI

The study on the BASMI incorporated 20 RCTs, encompassing a total of 1,004 patients and evaluating 11 distinct exercise interventions. The network relationships among these interventions are depicted in Figure 3C. Initially, each intervention was compared with conventional treatment through a traditional meta-analysis. The findings indicated that both AE (SMD = −0.85, 95% CI -1.40 to −0.29, P < 0.01) and qigong (SMD = −1.85, 95% CI -2.54 to −1.15, P < 0.01) demonstrated statistically significant improvements over conventional treatment, as illustrated in Supplementary Figure S9. In cases where the inconsistency model test yielded a P > 0.05, the consistency model was employed for NMA, as presented in Supplementary Figure S10. The NMA revealed that, in comparison to conventional treatment, (SMD = −0.96, 95% CI [-1.91, −0.02]), AE + Pilates (SMD = −3.33, 95% CI [-4.69, −1.96]), and qigong (SMD = −1.35, 95% CI [-2.43, −0.27]) all exhibited significant advantages, with the differences reaching statistical significance (P < 0.05). Pairwise comparisons revealed that AE + Pilates therapy was statistically superior to other exercise therapies, with significant differences observed (P < 0.05). In contrast, no statistically significant differences were found (P > 0.05) among other exercise therapies in pairwise comparisons. Detailed results of these comparisons are presented in Supplementary Table S7. Consequently, the findings from both routine and NMA are largely consistent. According to the SUCRA analysis, AE + Pilates (99.90%) emerges as the most effective intervention for reducing BASMI scores. The SUCRA values for the various intervention measures are illustrated in Figures 4, 5. To assess potential local inconsistencies among the intervention measures, we employed the node-splitting method, which indicated no local inconsistency (P < 0.05). Additionally, we performed a consistency test on the closed loop formed by the intervention measures in this study. Three closed loops were established, and the inconsistency analysis indicated that the CT-AE Qigong closed loop exhibited inconsistency, as evidenced by an IF > 1 (IF = 1.045, 95% CI [0, 2.15], P = 0.064), as presented in Table 1. Conventional meta-analysis was employed for direct pairwise comparisons to identify potential sources of heterogeneity. Notable heterogeneity was observed in the pairwise comparisons between CT, AE, and Qigong (I^2^ = 79.30%, p = 0.028). This suggests that the variability in therapeutic interventions may contribute to the observed heterogeneity. However, the pairwise comparison between AE and Qigong could not definitively determine heterogeneity due to the inclusion of only a single study, as illustrated in Supplementary Figures S11, 12.

### Chest expansion

The investigation into CE incorporated 17 RCTs encompassing a total of 781 patients and examining 9 distinct exercise interventions. The interventional network relationships are depicted in Figure 3D. Initially, each intervention was compared to conventional treatment through routine meta-analysis, which included AE + SE + Supervise (SMD = 0.80, 95% CI 0.17 to 1.43, P = 0.012), as illustrated in Supplementary Figure S13. In cases where the inconsistency model test yielded a P > 0.05, a consistency model was employed for the NMA, as demonstrated in Supplementary Figure S14. The NMA revealed that, in comparison to conventional treatment, both AE + SE + Supervise (SMD = 0.86, 95% CI [0.42, 1.29]) and the AE + SE (SMD = 0.46, 95% CI [0.05, 0.87]) exhibited significant advantages, with the differences being statistically significant (P < 0.05). The findings from both routine and NMA demonstrate a general consistency. Utilizing the SUCRA method, it can be inferred that supervised aerobic combined with SE (94.30%) is likely the most effective intervention for enhancing patients’ chest expansion. The SUCRA values for the various intervention strategies are presented in Figures 4, 5. Prior research has conclusively demonstrated that stretching exercises significantly enhance flexibility and muscle strength, aligning with the findings of the present study (Dutta et al., 2023). To assess potential local inconsistencies among the intervention measures, the node splitting method was employed, revealing a P < 0.05, which suggests the absence of local inconsistency. Furthermore, a consistency test was performed on the closed loops formed by the intervention measures in this study. Three closed loops were identified, and the loop inconsistency analysis indicated a lack of consistency within these loops, as detailed in Table 1.

### Cluster analysis

**FIGURE 6:**
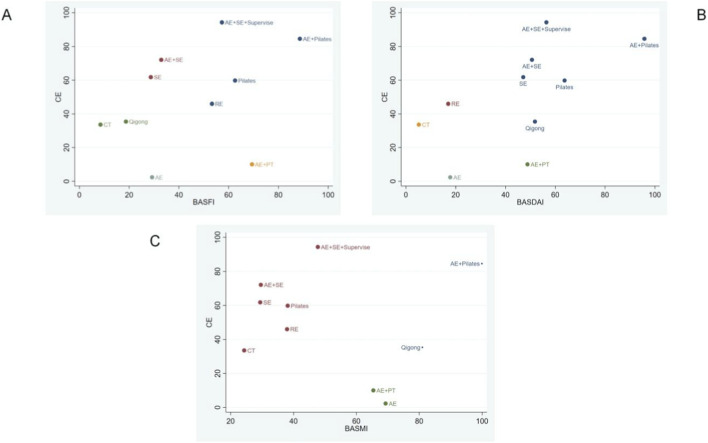
Cluster analysis plot of efficacy and degree of satisfaction. **(A)** BASFI score; **(B)** BASDAI score; **(C)** BASMI score.

Utilizing hierarchical cluster analysis with Ward’s linkage method and Euclidean distance measures, this study aims to identify the most effective exercise therapies by evaluating parameters such as the BASFI, the BASDAI, the BASMI, and CE. Cluster validity was assessed using silhouette analysis and Gap statistic. This methodological approach is grounded in the research framework established by Kuliś et al., employing cluster analysis of multidimensional biomechanical indicators to identify optimal exercise patterns (Kuliś et al., 2024). It substantiates the scientific validity of cluster analysis within the domain of ‘multi-indicator integration optimal solution screening’ research. This approach offers methodological support for the present study, facilitating the identification of optimal exercise interventions for AS through the clustering of clinical indicators. As illustrated in Figure 6, the results of the cluster analysis reveal that, in comparison to other exercise therapies, AE + Pilates emerges as the most advantageous approach. This therapy not only optimally enhances BASFI, BASDAI, and BASMI scores in patients with AS and reduces disease activity but also significantly aids in improving chest expansion.

### Evaluation of small sample effects

**FIGURE 7:**
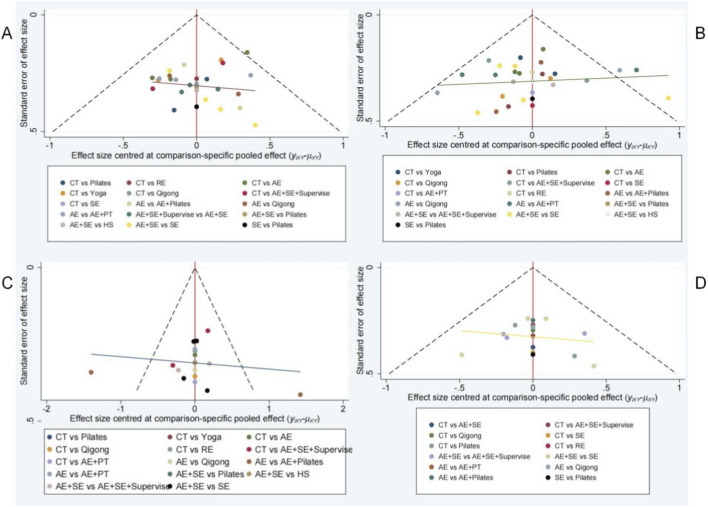
Funnel plot. **(A)** BASFI; **(B)** BASDAI; **(C)** BASMI; **(D)** CE.

The calibration comparison funnel plots for BASFI, BASDAI, BASMI, and CE demonstrate that the study points are approximately symmetrically distributed on either side of the midline. This symmetry suggests a minimal likelihood of publication bias within this study. The corresponding results are illustrated in Figure 7.

We employed a conventional meta-analytic approach to conduct funnel plot analysis and applied the Egger’s test to assess potential publication bias across the four evaluated outcome measures. The results of the Egger’s test indicated that, with the exception of the BASDAI indicator, the Egger’s values for the other indicators exceeded 0.05, suggesting an absence of publication bias. For the BASDAI indicator, even after applying the trim-and-fill method, the P-value remained below 0.01, indicating no evidence of result reversal and suggesting that the original findings were relatively robust.

#### Meta regression and subgroup analysis

Despite demonstrating low heterogeneity in this study through ring inconsistency testing and the node splitting method, we employed meta-regression to identify potential sources of heterogeneity and to thoroughly evaluate factors that could influence the results. By performing direct pairwise comparisons with CT, we conducted meta-regression analyses on variables such as publication year, different intervention measures, countries, sample sizes of the included studies, and exercise doses reported in the articles for the four outcome indicators. The results indicated statistically significant differences (P < 0.05) in publication year and sample size concerning the BASFI score, suggesting that these factors may be the primary sources of heterogeneity contributing to ring inconsistency in the BASFI score, as detailed in Supplementary Table S9. While we have successfully identified the primary source of heterogeneity, it is important to recognize that this remains a limitation of our study, which will be further elaborated upon in the Limitations section. We advise a cautious interpretation of the results regarding the efficacy of exercise therapy in improving BASFI scores. Future research should prioritize the implementation of advanced RCTs and adopt a large-sample, multicenter approach to furnish more robust and reliable evidence-based insights into the impact of exercise interventions on BASFI scores in patients with AS.

Following an evaluation of the risk quality of 8 studies identified as having a high risk of bias, a subgroup analysis was performed to ascertain the impact of these studies on the overall findings. The analysis revealed that the exclusion of articles with a high risk of bias did not alter the reduction in BASFI, BASDAI, and BASMI scores achieved through exercise therapy, thereby suggesting that the primary results are relatively robust, as illustrated in Supplementary Figures S15-17 and Supplementary Tables S10-12. Regarding the SUCRA score, the removal of high-risk studies indicated that simulated equestrian exercise remains potentially the most effective intervention for enhancing the BASFI score (89.10%), while AE + Pilates appears most effective for improving the BASDAI score (96.90%) and BASMI score (99.00%). These findings are entirely consistent with our previous research, underscoring the high stability of the main results, as demonstrated in Supplementary Table S13 and Supplementary Figures S18-20. Upon conducting a small sample effect evaluation and excluding high-risk studies, our analysis of the calibration comparison funnel plots for BASFI, BASDAI, and BASMI revealed that the data points were approximately symmetrically distributed around the midline. This suggests a relatively low likelihood of publication bias in this study. These findings are largely consistent with previous results, as illustrated in Supplementary Figures S21-23. In relation to CE, the construction of a network was not feasible following the exclusion of high-risk studies, as illustrated in Supplementary Figure S24. Consequently, a conventional meta-analysis was employed to perform pairwise comparisons. The results indicated that AE + SE + Supervise was superior not only to CT but also to the combination of AE and SE alone. Additionally, Qigong therapy demonstrated advantages over AE, aligning with findings from previous studies, as depicted in Supplementary Figures S25-27. Upon comparison with AE, a significant degree of heterogeneity was observed. Consequently, a sensitivity analysis was conducted using a stepwise exclusion method. Notably, a study focusing on Pilates from Turkey exhibited substantial heterogeneity. This observation led us to hypothesize that regional variations and differing interventions could be contributing factors to the observed heterogeneity, as illustrated in Supplementary Figure S28.

We conducted an evaluation of all pairwise comparisons related to the four outcome measures using the Confidence in Network Meta-Analysis (CINeMA) approach. For the BASF score, the quality of evidence supporting the efficacy of yoga therapy, AE + PT therapy, HS, and AE + Pilates in reducing the BASFI score was assessed as “moderate.” This suggests a reasonable level of confidence in the therapeutic benefits of these interventions. Regarding the BASDAI score, the evidence quality for the effectiveness of AE + PT and AE + Pilates in reducing the BASDAI score was also rated as “moderate.” Similarly, for the BASMI score, the evidence quality for the impact of qigong therapy and AE + Pilates was deemed “moderate.” Lastly, in the context of CE, AE + SE + Supervise, AE + Pilates, and qigong therapy was classified as “moderate.” The evidence quality for the aforementioned therapies is relatively robust. In contrast, the comparative evidence quality among other therapies was assessed as “low” or “very low,” reflecting a significant level of uncertainty. Caution is advised when implementing this conclusion in clinical practice. Further detailed evaluation results are available in Supplementary Tables S14-17. Based on these findings, we assert that additional high-quality research is necessary to further substantiate the efficacy of exercise therapy for patients with AS.

#### Dose response meta-analysis

##### BASFI

We identified a non-linear dose-response relationship between the total exercise dose of various intervention measures and the BASFI score. Specifically, when the AE dose reaches 987.25 MET minutes per week, further increases in exercise dose result in a detrimental effect on the BASFI score, as indicated by the upper limit of the 95% confidence interval being less than zero. The predicted response at this point is (SMD = −0.49; 95% CI [-0.99, 0.00]). The initial dose of AE is 210 MET minutes per week, suggesting that AE within the range of 210–987.25 MET minutes per week can effectively reduce the BASFI score in patients with AS. The minimum effective dose for AE + SE is 446.72 MET minutes per week (SMD = −0.40; 95% CI [-0.81, 0.00]), with an effective dose range of 446.72–2550 MET minutes per week. For AE + SE + Supervise, the minimum effective dose is 430.54 MET minutes per week (SMD = −0.58; 95% CI [-1.16, 0.00]), with an effective dose range of 430.54–2452 MET minutes per week. The initial qigong dosage is set at 180 MET minutes per week. Upon reaching a dosage of 217 MET minutes per week, further increases in exercise dosage result in a negative impact on the improvement of the BASFI score. The anticipated response is indicated by a SMD of −0.36, with a 95% CI ranging from −0.72 to 0. This suggests that qigong can effectively reduce the BASFI score in patients with AS within the dosage range of 180–217 MET minutes per week. The minimum effective dosage for SE is identified as 375.16 MET minutes per week, with an SMD of −0.68 and a 95% CI of [-1.36, 0.00]. The effective dosage range for SE is between 375.16 and 700 MET minutes per week. No statistically significant dosage intervals were observed for other exercise therapies. For further details, please refer to Supplementary Figures S29-37.

##### BASDAI

A non-linear dose-response relationship exists between the total exercise dose and the BASDAI score across various intervention measures. Specifically, when the dose of AE + PT therapy reaches 1,553.19 MET minutes per week, further increases in exercise dose result in a detrimental effect on the BASDAI score, as indicated by the upper limit of the 95% confidence interval being less than zero. The predicted response at this point is (SMD = −1.16; 95% CI [-2.31, 0.00]). The initial dose of AE + PT is 525 MET minutes per week, suggesting that AE can effectively reduce the BASDAI score in patients with AS within the dose range of 525–1,553.19 MET minutes per week. The minimum effective dose for aerobic combined with stretching exercise is 468.45 MET minutes per week (SMD = −0.47; 95% CI [-0.94, 0.00]), with an effective dose range of 468.45–1215 MET minutes per week. The minimum effective dose of supervised aerobic and stretching exercise is 691.5 MET minutes per week (SMD = −1.56; 95% CI [-3.12, 0]). The effective dose range for this type of exercise is between 691.5 and 2452 MET minutes per week. For qigong exercise, the minimum effective dose is 274.25 MET minutes per week (SMD = −1.98; 95% CI [-3.96, 0.00]), with a maximum effective dose of 378.36 MET minutes per week (SMD = −3.29; 95% CI [-6.58, 0.00]). The effective dose range for qigong is therefore 274.25 to 378.36 MET minutes per week. When the dose of stretching exercise reaches 2,225.14 MET minutes per week, there is an adverse effect on the improvement of the BASDAI score, as indicated by the upper limit of the 95% CI being less than 0 (SMD = −1.04; 95% CI [-2.08, 0.00]). The initial dose of stretching exercise is 315 MET minutes per week, suggesting that stretching exercise within the range of 315–2,225.14 MET minutes per week can effectively reduce the BASDAI score in patients with AS. No statistically significant dose interval was identified for other exercise therapies. For further details, refer to Supplementary Figures S38-46.

##### BASMI

A non-linear dose-response relationship exists between the total exercise dose and the BASMI score across various intervention measures. The minimum effective dose for AE + SE is 725.28 MET minutes per week (SMD = −0.30; 95% CI [-0.59, 0.00]). The effective dose range for this combination is 725.28–1215 MET minutes per week. For SE alone, the minimum effective dose is 510.71 MET minutes per week (SMD = −0.44; 95% CI [-0.87, 0.00]). The effective dose range for SE is 510.71–1225 MET minutes per week. No statistically significant dose interval was identified for other exercise therapies. For further details, refer to Supplementary Figures S47-54.

### Chest expansion

There exists a non-linear dose-response relationship between the total exercise dose and chest expansion across various intervention measures. Specifically, when the dose of AE + SE reaches 514.27 MET minutes per week, a beneficial effect on chest expansion becomes evident, as indicated by the lower limit of the 95% CI exceeding zero (MD = 0.30; 95% CI [0.00,0.60]). However, when the dose is increased to 1,607.36 MET minutes per week, further increases in the dose do not enhance chest expansion in patients with AS (MD = 0.44; 95% CI [0.00,0.90]). Therefore, it can be concluded that AE + SE effectively enhances chest expansion in AS patients within the dose range of 514.27–1,607.36 MET minutes per week. For further details, refer to Supplementary Figures S55-60.

## Discussion

### Principal findings

Our study presents a comprehensive network meta-analysis examining the efficacy of exercise therapy in the management of AS. The findings from this meta-analysis underscore the potential benefits of exercise therapy in enhancing disease activity and physical function in individuals with AS. Our analysis incorporated data from 32 studies, encompassing a total of 1,757 participants, to evaluate the effectiveness of various exercise therapies compared to conventional treatments. Utilizing the NMA method, we evaluated the SUCRA of various efficacy indicators for all exercise therapies in the treatment of AS to determine the most effective intervention. The study revealed that simulated equestrian sports may be the most effective in reducing BASFI scores. Aerobic and Pilates exercises demonstrated the greatest efficacy in decreasing BASDAI and BASMI scores among AS patients. Furthermore, AE + SE + Supervise appeared to enhance CE more effectively in AS patients. Additionally, our findings indicated that exercise therapy generally does not lead to severe adverse reactions, with patients exhibiting good tolerance and a reliable safety profile.

We identified a non-linear dose-response relationship between exercise and the indices BASFI, BASDAI, BASMI, and CE. The term “exercise dose” encompasses the combination of exercise intensity and duration over a specified period, with both exercise dose and MET serving as crucial metrics for assessing exercise intensity and energy expenditure. MET is a standard measure for evaluating the intensity of physical activity, commonly used to estimate an individual’s energy expenditure and physical activity level. One MET is defined as the amount of oxygen consumed per kilogram of body weight per minute at rest, approximately 3.5 mL of oxygen per kilogram of body weight per minute (Douglas et al., 2022). Regarding the BASFI score,our findings suggest that the minimum effective dose of Qigong is 180 MET minutes per week, which is equivalent to walking for 51 min (3.50 METs/min) and cycling for 45 min (4 METs/min) per week (Ainsworth et al., 2011). Conversely, the maximum effective dose for AE + SE can reach 2550 MET minutes per week. In relation to BASDAI, the results indicate that the minimum effective dose of Qigong is 274.25 MET minutes per week, while the maximum effective dose for SE is 2,225.24 MET minutes per week. In relation to the BASMI score, our study identifies that the minimum effective dose for AE + SE is 725.28 MET minutes per week, while SE alone remain effective at 1225 MET minutes per week. Regarding CE, AE + SE demonstrate efficacy at 514.27 MET minutes per week and continue to enhance chest expansion in patients with AS up to a maximum dose of 1,607.36 MET minutes per week. The World Health Organization (WHO) advises an upper limit of 1200 MET minutes per week for energy expenditure and physical activity levels. However, our findings indicate that for patients with AS, engaging in exercise beyond the WHO’s recommended upper limit of 1200 MET minutes per week yields additional benefits (Bull et al., 2020). This observation aligns with existing literature, which suggests a strong correlation between the exercise habits and physical activity levels of individuals with ankylosing spondylitis and their disease activity. Specifically, the weekly total energy expenditure (MET) of patients experiencing high disease activity was significantly lower than that of patients with low disease activity and the healthy control group (Fongen et al., 2013). These results imply that increasing physical activity levels may be advantageous for patients experiencing high disease activity.

### Comparison with previous studies

Previous research has demonstrated that various exercise interventions are advantageous for patients with AS. For instance, home-based exercise has been shown to enhance quality of life-related indicators (Liang et al., 2015), while activities such as running, Pilates, and yoga have been found to be more effective than traditional therapies in alleviating symptoms (Luo et al., 2024). Nevertheless, existing meta-analyses predominantly concentrate on the effects of individual exercise interventions lacking systematic horizontal comparisons across multiple exercise modalities (Hu et al., 2020). Furthermore, a network meta-analysis conducted in 2024 included only 10 RCTs, with limited coverage of intervention measures (Luo et al., 2024). Additionally, the influence of exercise dosage on rehabilitation outcomes remains unclear, complicating the formulation of precise exercise prescriptions. These research gaps underscore the need for comprehensive and systematic analyses.

### Explanation of the research results

#### Why choose exercise therapy

##### The effectiveness of HS therapy

HS, alternatively referred to as equine-assisted therapy, is gaining recognition for its potential therapeutic benefits in addressing a variety of conditions, particularly those associated with posture control and psychosocial enhancement. This therapeutic approach leverages the movement of horses to engage patients in physical activities that promote balance, coordination, and overall postural stability (Sterba et al., 2002; Park et al., 2014). For safety considerations, the present study did not employ actual equestrian sports; instead, it utilized simulated equestrian activities through specialized equipment. A preliminary investigation assessed the effects of equestrian rehabilitation (ER) and Ono therapy on adult patients with intellectual disabilities. The findings indicated significant improvements in the patients’ autonomy and social integration capabilities. The study employed assessment tools grounded in the International Classification of Functioning, Disability, and Health for Children and Adolescents (ICF-CY) to evaluate outcomes, revealing that both ER and Ono therapy yielded substantial benefits over time, with enduring advancements in both physical and psychosocial performance (Borioni et al., 2012). Numerous studies have demonstrated that equestrian therapy can enhance motor function and posture control in children with cerebral palsy (Sterba, 2007). The effects of AS on posture are complex and multifaceted. AS may result in reduced spinal mobility and flexibility, subsequently impacting balance and gait. Research indicates that individuals with AS often experience balance disorders compared to healthy individuals, primarily due to restricted spinal activity and pronounced kyphosis (Gündüz et al., 2017). Furthermore, the progression of AS can lead to the development of osteophytes along the spine, characterized by bony hyperplasia, which exacerbates spinal stiffness and postural alterations. The formation of osteophytes is associated with an imbalance in bone metabolism, driven by inflammatory cytokines. This imbalance is more pronounced in male patients, who generally exhibit higher levels of bone resorption markers and inflammatory cytokines than female patients (Huang et al., 2012). Consequently, equestrian activities can significantly improve the physical balance of AS patients, enhance spinal mobility, and strengthen coordination, thereby substantially improving patients’ quality of life.

##### The effectiveness of AE therapy

AE has been demonstrated to exert numerous beneficial effects on patients with AS. One study assessing the efficacy of aerobic training in individuals with AS reported significant enhancements in walking distance and aerobic capacity; however, it did not observe additional benefits in functional ability, activity levels, disease activity, or quality of life when compared to SE alone (Jennings et al., 2015). Conversely, another study investigating the impact of swimming, walking, and traditional exercise on lung function, aerobic capacity, and quality of life in AS patients concluded that AE, such as swimming and walking, in conjunction with traditional exercise, augmented patients’ functional abilities and enhanced their quality of life (Karapolat et al., 2009). Furthermore, this study underscores the importance of regular AE in alleviating pain and stiffness, thereby contributing to improved overall disease management (Regnaux et al., 2019). The advantages of AE extend beyond mere physical enhancement. A research study investigated the interconnections among body mass index (BMI), disease activity, and exercise in patients with AS, revealing that an elevated BMI correlates with heightened disease activity. The findings of this study indicate that AE could potentially mitigate some of the negative consequences associated with a high BMI, including increased inflammation and disease activity (Liew et al., 2022).

##### The effectiveness of pilates therapy

Pilates is recognized for its focus on core strength, flexibility, and posture, attributes that may be particularly advantageous for individuals with AS. This popular form of exercise aims to enhance the strength and endurance of core muscles, core stability, and joint flexibility through a comprehensive series of whole-body exercises. Empirical studies have demonstrated that Pilates can improve body composition, muscle endurance, and joint flexibility (Karpouzi et al., 2025). Furthermore, Pilates training has been shown to effectively alleviate pain, enhance functional levels, and increase core muscle thickness in individuals suffering from chronic lower back pain (Batıbay et al., 2021). Additionally, Pilates is considered to positively influence the daily lives of patients with chronic musculoskeletal disorders by fostering improvements in physical function and self-management skills (Gaskell and Williams, 2019). Beyond its muscular benefits, Pilates also positively impacts bone health. Research indicates that Pilates training can increase bone density, particularly in postmenopausal women with osteoporosis, thereby enhancing bone density, physical performance, and quality of life (Ang et al., 2015). Moreover, Pilates is believed to improve balance in the elderly, thereby reducing the risk of falls (Moreno-Segura et al., 2018). Regarding joint health, Pilates is purported to enhance joint stability and function through the improved control of core musculature. Empirical evidence indicates that Pilates exercises can effectively engage both deep and superficial abdominal muscles, thereby augmenting the stability of the lumbar spine (Moreno-Segura et al., 2018). Furthermore, Pilates has been employed to enhance core muscle activation in individuals with chronic low back pain, highlighting its potential to improve core muscle strength. AS is a chronic inflammatory condition primarily affecting the spine, sacroiliac joints, and adjacent soft tissues. Individuals with AS often experience bone fusion and new bone formation in the spine and sacroiliac joints, resulting in significant functional impairment and pain (Magrey and Khan, 2017). Muscle tissue atrophy and fat degeneration are common in AS patients, contributing to restricted spinal mobility. These patients typically exhibit a reduced lumbar multifidus muscle area and an increased degree of fat degeneration, which may exacerbate pain and functional limitations (Ozturk and Yagci, 2021). Considering the pathological characteristics of AS, Pilates exercises can effectively support the musculoskeletal system and enhance vertebral stability, thereby alleviating pain. Our study indicates that combination therapy generally offers significant benefits, with the integration of aerobic and Pilates exercises demonstrating a synergistic effect in the rehabilitation of ankylosing spondylitis. This approach markedly enhances patients’ disease activity, functional status, and quality of life. Furthermore, the combined exercise regimen not only augments physical function but also positively influences psychological well-being, rendering it a promising rehabilitation strategy for broader implementation. Pilates places a strong emphasis on core muscle stability and overall body coordination, offering potential benefits for enhancing the biomechanical function of patients with AS. Studies indicate that individuals with AS frequently experience structural damage and inflammation of the spine, resulting in sagittal plane imbalance (Li et al., 2021). Pilates may ameliorate this imbalance by strengthening and increasing the flexibility of the core muscles. In AS patients, spinal stiffness and chest deformation often contribute to restricted respiratory function. Research has identified a correlation between sagittal rotation of the diaphragm and pulmonary dysfunction in these patients (Liu et al., 2019). The respiratory training component of Pilates may enhance diaphragm activity, thereby positively influencing lung function. Post-exercise tremors in the lower limb muscles, indicative of neuromuscular conduction efficiency and the degree of muscle activation, have been substantiated in studies focusing on speed skaters. The present study demonstrates that the frequency and amplitude of these tremors serve as objective indicators of the exercise load’s impact on the neuromuscular system, thereby affirming that targeted exercise interventions can enhance the synergistic interaction between muscles and nerves (Kuliś et al., 2025). Furthermore, Pilates practice can improve posture and balance, which are essential for the daily activities of individuals with AS (Sawacha et al., 2012).

#### Respiratory function exercises for individuals diagnosed with AS

AS frequently results in restricted spinal mobility and diminished chest expansion. Research indicates that lung function in AS patients is generally lower than in the general population, with restrictive ventilation disorders being more prevalent (Berdal et al., 2012). The reduction in CE is closely associated with decreased spinal mobility, particularly in AS patients, where spinal stiffness and deformity can further compromise respiratory function (Szewczyk et al., 2024). AE can enhance cardiovascular function and overall endurance in AS patients, thereby aiding in the improvement of their respiratory function. Studies have demonstrated that appropriate AE can effectively increase lung capacity and respiratory muscle strength, thereby enhancing respiratory function (Basbug et al., 2023). The integration of aerobic and stretching exercises holds significant importance in the management of AS patients. By enhancing respiratory function, spinal mobility, and overall health, this exercise regimen can assist patients in effectively managing disease-related challenges and improving their quality of life. Our research also revealed that supervised exercise programs yield more favorable outcomes compared to unsupervised ones, facilitating better rehabilitation and quality of life improvements for AS patients. This is primarily because supervision offers more effective guidance and feedback, thereby enhancing the efficacy of the training. Under the guidance of professional physical therapists, exercise regimens can be adjusted promptly and effectively, thereby increasing the specificity and effectiveness of the treatment. Consequently, we recommend that supervision be considered a crucial component of exercise therapy for AS, and it should be consistently integrated throughout the treatment process to optimize therapeutic outcomes. Prior research has demonstrated that digital interventions possess significant scalability and can effectively reach individuals with chronic pain. Looking ahead, the implementation of additional digital intervention therapies for patients with ankylosing spondylitis may enhance the monitoring of their exercise regimens and facilitate the development of personalized exercise prescriptions (Blake et al., 2024).

#### Exploring the therapeutic potential of qigong in the management of AS

Our research indicates that qigong may be a highly promising exercise modality for ameliorating spinal joint symptoms in patients with ankylosing spondylitis. Notably, the required exercise dosage for qigong is minimal compared to other forms of exercise, making it particularly suitable for patients with comorbid conditions that preclude vigorous physical activity, as well as for elderly individuals. As a traditional exercise rooted in Chinese medicine, qigong has garnered significant attention in recent years due to its potential benefits in enhancing joint and muscle function. Empirical studies have demonstrated that qigong practice substantially improves postural stability, joint proprioception, and symptomatology in patients with knee osteoarthritis (Ye et al., 2020). For instance, the Eight Section Brocade Qigong has been shown to significantly enhance knee joint proprioception and postural stability, while concurrently alleviating pain, stiffness, and functional impairment in elderly patients (Ye et al., 2020). Furthermore, Wuqinxi Qigong has exhibited positive effects on the physical function of patients with early-stage knee osteoarthritis. Compared to conventional physical therapy, Wuqinxi Qigong is more efficacious in promoting balance and alleviating pain (Xiao et al., 2020). These findings underscore the potential of qigong as a therapeutic exercise intervention. The influence of qigong on muscle strength is noteworthy. Empirical studies have demonstrated that qigong practice can substantially enhance muscle strength and static posture control in middle-aged and elderly women (Carcelén-Fraile et al., 2021). Additionally, qigong has been shown to improve muscle strength in young women and contribute to physical health by mitigating oxidative stress and augmenting antioxidant capacity (Klarod et al., 2020). These findings suggest that qigong not only aids in strengthening muscles but also promotes overall health by modulating oxidative stress and maintaining antioxidant balance. In conclusion, qigong, a traditional exercise form rooted in Chinese medicine, exhibits significant benefits in enhancing joint and muscle function. It has proven effective in alleviating symptoms of knee osteoarthritis and improving muscle strength and posture control. Although research on the application of qigong for ankylosing spondylitis is limited in scope and sample size, its potential warrants further investigation. Future large-scale, multicenter RCTs should be conducted to validate the efficacy of qigong in managing ankylosing spondylitis.

## Limitations

Nevertheless, our research is subject to several limitations that warrant acknowledgment. Firstly, given that AS is a chronic condition, the majority of participants in the study were concurrently undergoing treatment with immunosuppressants or biologics. It is crucial to recognize that these confounding variables could not be accounted for in the analysis, necessitating caution in the interpretation of these findings. Secondly, the dose-response analysis of certain exercises did not yield significant results. This may be attributed not only to the inherent limitations of the exercises themselves but also to the insufficient dose range covered by existing studies on specific exercises, which may not be adequate to detect meaningful and significant dose effects. Consequently, we should exercise caution in interpreting dose prediction results and emphasize the need for future research to focus on the effects of varying exercise doses on AS patients. In future research, large-scale multicenter randomized controlled trials should be conducted to unify measurement equipment and record relevant parameters in detail, thereby enhancing the rigor of the methodology. Thirdly, although we included 32 studies, the relatively small number of studies remains a significant limitation that could potentially bias the results. Lastly, the comprehensive nature of this analysis, which encompasses a substantial number of studies, inevitably introduces high heterogeneity in certain intervention measures. Through the application of sensitivity analysis and meta-regression, we identified potential factors contributing to significant heterogeneity and successfully mitigated them to an acceptable range via subgroup analysis. While elevated heterogeneity can compromise the reliability and generalizability of findings, our enhancements to the subgroup analysis effectively address this concern. We advocate for future research designs to achieve greater consistency, particularly regarding measurement tools and selection criteria for study populations, to minimize the impact of heterogeneity. Lastly, due to the inherent challenges in implementing double-blind procedures and simulations for exercise prescriptions, the quality of evidence in this study was assessed as ranging from extremely low to moderate, which may further influence the overall quality of the evidence.

## Conclusion

In conclusion, this network meta-analysis and dose-response meta-analysis synthesize existing evidence from prior studies and offer theoretical insights into exercise therapy for clinical practice and academic research. Our comprehensive analysis confirms the efficacy of various exercise interventions in reducing activity levels and enhancing chest expansion in patients with AS. We identified a non-linear dose-response relationship between exercise and both disease activity levels and chest expansion. The evidence, albeit of low quality, indicates that simulated equestrian exercise, a combination of aerobic and Pilates exercises, and AE + SE + Supervise may be effective in improving disease activity and chest expansion in AS patients. Moreover, traditional sports qigong demonstrates notable efficacy concerning exercise dosage, requiring a lower dose compared to other exercises to achieve its effects. It is advisable to adjust exercise intensity based on the physical condition of AS patients. Future large-scale RCTs are warranted to explore the impact of different exercise dosages on AS.

## Data Availability

The datasets presented in this study can be found in online repositories. The names of the repository/repositories and accession number(s) can be found in the article/Supplementary Material.
