# Supplementary material for: The effectiveness of different exercise mode interventions in improving disease activity in patients with ankylosing spondylitis: a network and dose-dependent meta-analysis
Source: Front Physiol. 2026 Jan 20;16:1715944. doi: 10.3389/fphys.2025.1715944 (PMC12864062; doi:10.3389/fphys.2025.1715944)
Supplement: Supplementary file 1 [file Supplementaryfile1.docx]

**SUPPLEMENTARY MATERIAL**

**Table of Contents**

**Supplementary Methods 1 –** Database search strategy.

**Supplementary Methods** **2 –** Conversion formula for standard deviation (SD).

**Supplementary Table S1** **–** Exercise Therapy Classification.

**Supplementary Table S2 –** Explanation of technical terms.

**Supplementary Table S****3 –** Demographic characteristics of included studies

# **Supplementary Table S4** **–** Details of interventions conducted in the trial.

**Supplementary Table S5 –** Results of network Meta-Analysis(BASFI).

**Supplementary Table S6 –** Results of network Meta-Analysis(BASDAI).

**Supplementary** **Table S7** **–** Results of network Meta-Analysis(BASMI).

**Supplementary Table S8 –** Results of network Meta-Analysis(CE).

**Supplementary Table S9 –** Meta Regression.

**Supplementary Table S10 –** Results of network Meta-Analysis(BASFI, Removal of High Risk Studies)

**Supplementary Table S11 –** Results of network Meta-Analysis(BASDAI, Removal of High Risk Studies).

**Supplementary Table S12 –**Results of network Meta-Analysis(BASMI, Removal of High Risk Studies).

**Supplementary Table S13 –** The SUCRA Values of Each Treatment Modality (Removal of High Risk Studies).

**Supplementary Table S14 –** The Grading of Recommendations Assessment, Development and Evaluation (GRADE) assessment for BASFI.

**Supplementary Table S15 –** The Grading of Recommendations Assessment, Development and Evaluation (GRADE) assessment for BASDAI.

**Supplementary Table S16 –** The Grading of Recommendations Assessment, Development and Evaluation (GRADE) assessment for BASMI.

**Supplementary Table S17 –** The Grading of Recommendations Assessment, Development and Evaluation (GRADE) assessment for CE.

**Supplementary Figure S1 –** Risk of bias assessment included in the study.

**Supplementary Figure S2 –** A pairwise comparison of the BASFI scores was conducted between all exercise interventions and conventional therapy(CT).

**Supplementary Figure S3 –** Non-concordance testing for BASFI scores.

**Supplementary Figure S4 –** A pairwise comparison was conducted among the BASFI scores, AE + SE + Supervise, and CT, as well as between SE and CT.

**Supplementary Figure S5 –** BASFI score, pairwise comparison between AE + SE + Supervise and AE + SE.

**Supplementary Figure S6 –** BASFI score, pairwise comparison between AE + SE and SE.

**Supplementary Figure S7 –** A pairwise comparison of the BASDAI scores was conducted between all exercise interventions and CT.

**Supplementary Figure S8 –** Non-concordance testing for BASDAI scores.

**Supplementary Figure S9 –** A pairwise comparison of the BASMI scores was conducted between all exercise interventions and CT.

**Supplementary Figure S10 –** Non-concordance testing for BASMI scores.

**Supplementary Figure S11 –** BASMI score, pairwise comparison of AE and Qigong with CT.

**Supplementary Figure S12 –** Sensitivity analysis of pairwise comparison between AE, Qigong, and CT.

**Supplementary Figure S13 –** A pairwise comparison of the CE was conducted between all exercise interventions and CT.

**Supplementary Figure S14 –** Non-concordance testing for CE.

**Supplementary Figure S15 –** BASFI network diagram after removing high-risk studies.

**Supplementary Figure S16 –** BASDAI network diagram after removing high-risk studies.

**Supplementary Figure S17 –** BASMI network diagram after removing high-risk studies.

**Supplementary Figure S18 –** BASFI score SUCRA chart for high-risk research removal.

**Supplementary Figure S19 –** BASDAI score SUCRA chart for high-risk research removal.

**Supplementary Figure S20 –** BASDAI score SUCRA chart for high-risk research removal.

**Supplementary Figure S21 –** BASFI scoring funnel plot for removing high-risk studies.

**Supplementary Figure S22 –** BASDAI scoring funnel plot for removing high-risk studies.

**Supplementary Figure S23 –** BASMI scoring funnel plot for removing high-risk studies.

**Supplementary Figure S24 –** CE network diagram after removing high-risk studies.

**Supplementary Figure S25 –** A pairwise comparison of the CE was conducted between all exercise interventions and CT(removal of High Risk Studies).

**Supplementary Figure S26 –** A direct pairwise comparison was conducted between SE and AE, as well as between AE+SE+Supervise and AE(removal of High Risk Studies).

**Supplementary Figure S27 –** A pairwise comparison was conducted between AE+Pilates, AE+PT, and Qigong, each directly compared with AE(removal of High Risk Studies).

**Supplementary Figure S28 –** Sensitivity analysis of pairwise comparison between AE+Pilates, AE+PT, Qigong and CT(removal of High Risk Studies).

**Supplementary Figure S29 –** Dose changes of AE reducing BASFI score.

**Supplementary Figure S30 –** Dose changes of AE+Pilates reducing BASFI score.

**Supplementary Figure S31 –** Dose changes of AE+PT reducing BASFI score.

**Supplementary Figure S32 –** Dose changes of AE+SE reducing BASFI score.

**Supplementary Figure S33 –** Dose changes of AE+SE+Supervise reducing BASFI score.

**Supplementary Figure S34 –** Dose changes of Pilates reducing BASFI score.

**Supplementary Figure S35 –** Dose changes of Qigong reducing BASFI score.

**Supplementary Figure S36 –** Dose changes of SE reducing BASFI score.

**Supplementary Figure S37 –** Dose changes of Yoga reducing BASFI score.

**Supplementary Figure S38 –** Dose changes of AE reducing BASDAI score.

**Supplementary Figure S39 –** Dose changes of AE+Pilates reducing BASDAI score.

**Supplementary Figure S40 –** Dose changes of AE+PT reducing BASDAI score.

**Supplementary Figure S41 –** Dose changes of AE+SE reducing BASDAI score.

**Supplementary Figure S42 –** Dose changes of AE+SE+Supervise reducing BASDAI score.

**Supplementary Figure S43 –** Dose changes of Pilates reducing BASDAI score.

**Supplementary Figure S44 –** Dose changes of Qigong reducing BASDAI score.

**Supplementary Figure S45 –** Dose changes of SE reducing BASDAI score.

**Supplementary Figure S46 –** Dose changes of SE reducing BASDAI score.

**Supplementary Figure S47 –** Dose changes of AE reducing BASMI score.

**Supplementary Figure S48 –** Dose changes of AE+Pilates reducing BASMI score.

**Supplementary Figure S49 –** Dose changes of AE+PT reducing BASMI score.

**Supplementary Figure S50 –** Dose changes of AE+SE reducing BASMI score.

**Supplementary Figure S51 –** Dose changes of AE+SE+Supervise reducing BASMI score.

**Supplementary Figure S52 –** Dose changes of Pilates reducing BASMI score.

**Supplementary Figure S53 –** Dose changes of Qigong reducing BASMI score.

**Supplementary Figure S54 –** Dose changes of SE reducing BASMI score.

**Supplementary Figure S55 –** Dose variation of AE to enhance chest expansion.

**Supplementary Figure S56 –** Dose variation of AE+SE to enhance chest expansion.

**Supplementary Figure S57 –** Dose variation of AE+SE+Supervise to enhance chest expansion.

**Supplementary Figure S58 –** Dose variation of Pilates to enhance chest expansion.

**Supplementary Figure S59 –** Dose variation of Qigong to enhance chest expansion.

**Supplementary Figure S60 –** Dose variation of SE to enhance chest expansion.

**Supplementary Methods 1 –** Database search strategy.

**Pubmed**

#1

(Spondylitis, Ankylosing[MeSH Terms]) OR (Bechterew's Disease[Title/Abstract]) OR (Bechterews Disease[Title/Abstract]) OR (Marie-Struempell Disease[Title/Abstract]) OR (Marie Struempell Disease[Title/Abstract]) OR (Spondylarthritis Ankylopoietica[Title/Abstract]) OR (Rheumatoid Spondylitis[Title/Abstract]) OR (Spondylitis, Rheumatoid[Title/Abstract]) OR (Ankylosing Spondylitis[Title/Abstract]) OR (Ankylosing Spondylarthritis[Title/Abstract]) OR (Ankylosing Spondylarthritides[Title/Abstract]) OR (Spondylarthritides, Ankylosing[Title/Abstract]) OR (Spondylarthritis, Ankylosing[Title/Abstract]) OR (Ankylosing Spondyloarthritis[Title/Abstract]) OR (Ankylosing Spondyloarthritides[Title/Abstract]) OR (Spondyloarthritides, Ankylosing[Title/Abstract]) OR (Spondyloarthritis, Ankylosing[Title/Abstract]) OR (Spondylitis Ankylopoietica[Title/Abstract]) OR (Bechterew Disease[Title/Abstract]) OR (Spondyloarthritis Ankylopoietica[Title/Abstract]) OR (Spondylitis, Ankylosing[Title/Abstract])

#2

(Exercise Therapy[MeSH Terms]) OR (Exercise Therapy[Title/Abstract]) OR (Rehabilitation Exercise[Title/Abstract]) OR (Exercise, Rehabilitation[Title/Abstract]) OR (Exercises, Rehabilitation[Title/Abstract]) OR (Rehabilitation Exercises[Title/Abstract]) OR (Rehabilitation Exercises[Title/Abstract]) OR (Therapy, Exercise[Title/Abstract]) OR (Exercise Therapies[Title/Abstract]) OR (Therapies, Exercise[Title/Abstract]) OR (Remedial Exercise[Title/Abstract]) OR (Exercise, Remedial[Title/Abstract]) OR (Exercises, Remedial[Title/Abstract]) OR (Remedial Exercises[Title/Abstract])

#3

(Muscle Stretching Exercises[MeSH Terms]) OR (Exercise, Muscle Stretching[Title/Abstract]) OR (Muscle Stretching Exercise[Title/Abstract]) OR (Active Stretching[Title/Abstract]) OR (Stretching, Active[Title/Abstract]) OR (Static-Active Stretching[Title/Abstract]) OR (Static Active Stretching[Title/Abstract]) OR (Stretching, Static-Active[Title/Abstract]) OR (Proprioceptive Neuromuscular Facilitation (PNF) Stretching[Title/Abstract]) OR (Proprioceptive Neuromuscular Facilitation[Title/Abstract]) OR (Neuromuscular Facilitation, Proprioceptive[Title/Abstract]) OR (Proprioceptive Neuromuscular Facilitations[Title/Abstract]) OR (PNF Stretching[Title/Abstract]) OR (PNF Stretchings[Title/Abstract]) OR (Stretching, PNF[Title/Abstract]) OR (PNF Stretching Exercise[Title/Abstract]) OR (Exercise, PNF Stretching[Title/Abstract]) OR (PNF Stretching Exercises[Title/Abstract]) OR (Stretching Exercise, PNF[Title/Abstract]) OR (Passive Stretching[Title/Abstract]) OR (Stretching, Passive[Title/Abstract]) OR (Static-Passive Stretching[Title/Abstract]) OR (Static Passive Stretching[Title/Abstract]) OR (Stretching, Static-Passive[Title/Abstract]) OR (Relaxed Stretching[Title/Abstract]) OR (Stretching, Relaxed[Title/Abstract]) OR (Static Stretching[Title/Abstract]) OR (Stretching, Static[Title/Abstract]) OR (Isometric Stretching[Title/Abstract]) OR (Stretching, Isometric[Title/Abstract]) OR (Dynamic Stretching[Title/Abstract]) OR (Stretching, Dynamic[Title/Abstract]) OR (Ballistic Stretching[Title/Abstract]) OR (Stretching, Ballistic[Title/Abstract])

#4

(Aquatic Therapy[MeSH Terms]) OR (Hydrotherapy[Mesh]) OR (Therapy, Aquatic[Title/Abstract]) OR (Pool Therapy[Title/Abstract]) OR (Therapy, Pool[Title/Abstract]) OR (Ai Chi Therapy[Title/Abstract]) OR (Therapies, Ai Chi[Title/Abstract]) OR (Therapy, Ai Chi[Title/Abstract]) OR (Water Tai Chi Therapy[Title/Abstract]) OR (Aquatic Exercise Therapy[Title/Abstract]) OR (Exercise Therapy, Aquatic[Title/Abstract]) OR (Therapy, Aquatic Exercise[Title/Abstract]) OR (Water Exercise Therapy[Title/Abstract]) OR (Exercise Therapy, Water[Title/Abstract]) OR (Therapy, Water Exercise[Title/Abstract]) OR (Water-based Exercise[Title/Abstract]) OR (Aqua[Title/Abstract])

#5

(Resistance Training[MeSH Terms]) OR (Resistance Training[Title/Abstract]) OR (Training, Resistance[Title/Abstract]) OR (Strength Training[Title/Abstract]) OR (Training, Strength[Title/Abstract]) OR (Weight-Lifting Strengthening Program[Title/Abstract]) OR (Strengthening Programs, Weight-Lifting[Title/Abstract]) OR (Strengthening Program, Weight-Lifting[Title/Abstract]) OR (Weight Lifting Strengthening Program[Title/Abstract]) OR (Weight-Lifting Strengthening Programs[Title/Abstract]) OR (Weight-Lifting Exercise Program[Title/Abstract]) OR (Exercise Programs, Weight-Lifting[Title/Abstract]) OR (Exercise Program, Weight-Lifting[Title/Abstract]) OR (Weight Lifting Exercise Program[Title/Abstract]) OR (Weight-Lifting Exercise Programs[Title/Abstract]) OR (Weight-Bearing Strengthening Program[Title/Abstract]) OR (Strengthening Programs, Weight-Bearing[Title/Abstract]) OR (Strengthening Program, Weight-Bearing[Title/Abstract]) OR (Weight Bearing Strengthening Program[Title/Abstract]) OR (Weight-Bearing Strengthening Programs[Title/Abstract]) OR (Weight-Bearing Exercise Program[Title/Abstract]) OR (Exercise Programs, Weight-Bearing[Title/Abstract]) OR (Exercise Program, Weight-Bearing[Title/Abstract]) OR (Weight Bearing Exercise Program[Title/Abstract]) OR (Weight-Bearing Exercise Programs[Title/Abstract]) OR (Muscle Strength Training[Title/Abstract])

#6

(Circuit-Based Exercise[MeSH Terms]) OR (Circuit Based Exercise[Title/Abstract]) OR (Circuit-Based Exercises[Title/Abstract]) OR (Exercise, Circuit-Based[Title/Abstract]) OR (Exercises, Circuit-Based[Title/Abstract]) OR (Circuit Training[Title/Abstract]) OR (Training, Circuit[Title/Abstract])

#7

"Yoga"[Mesh] OR "Tai Ji"[Mesh] OR (Tai-ji) OR (Tai Chi) OR (Chi, Tai) OR (Tai Ji Quan) OR (Ji Quan, Tai) OR (Quan, Tai Ji) OR (Taiji) OR (Taijiquan) OR (T'ai Chi) OR (Tai Chi Chuan) OR ("Qigong"[Mesh]) OR (Qi Gong OR Ch'i Kung) OR ("Exercise Movement Techniques"[Mesh]) OR (Movement Techniques, Exercise) OR (Exercise Movement Technics) OR (Pilates-Based Exercises) OR (Exercises, Pilates-Based) OR (Pilates Based Exercises) OR (Pilates Training) OR (Training, Pilates) OR (McKenzie method) OR (Heckscher training) OR (running) OR (jogging) OR (exergame) OR (swiss balls)

#8

("Breathing Exercises"[Mesh]) OR (Inspiratory muscle training) OR (Expiratory muscle training) OR (Exercise, Breathing) OR (Respiratory Muscle Training) OR (Muscle Training, Respiratory) OR (Training, Respiratory Muscle)

#9

("Exercise"[Mesh]) OR ("High-Intensity Interval Training"[Mesh]) OR (High Intensity Interval Training) OR (High-Intensity Interval Trainings) OR (Interval Training, High-Intensity) OR (Interval Trainings, High-Intensity) OR (Training, High-Intensity Interval) OR (Trainings, High-Intensity Interval) OR (High-Intensity Intermittent Exercise) OR (Exercise, High-Intensity Intermittent) OR (Exercises, High-Intensity Intermittent) OR (High-Intensity Intermittent Exercises) OR (Sprint Interval Training) OR (Sprint Interval Trainings) OR (Interval Training) OR (Aerobic Interval Training)

#10

(balance training[Title/Abstract]) OR (balance trainings[Title/Abstract]) OR (balance exercise[Title/Abstract]) OR (balance exercises [Title/Abstract]) OR (flexibility training[Title/Abstract]) OR (flexibility trainings[Title/Abstract]) OR (flexibility exercise[Title/Abstract]) OR (flexibility exercises[Title/Abstract]) OR (joint mobility training[Title/Abstract]) OR (joint mobility trainings[Title/Abstract]) OR (joint mobility exercise[Title/Abstract]) OR (joint mobility exercises[Title/Abstract]) OR (functional movement training[Title/Abstract]) OR(functional movement trainings[Title/Abstract]) OR (functional movement exercise[Title/Abstract]) OR (functional movement exercises[Title/Abstract]) OR (stability training[Title/Abstract]) OR (stability trainings[Title/Abstract]) OR (stability exercise[Title/Abstract]) OR (stability exercises[Title/Abstract]) OR (medical gymnastics[Title/Abstract])

#11

(randomized controlled trial [pt] OR "controlled clinical trial"[Publication Type] OR "randomized"[Title/Abstract] OR "placebo"[Title/Abstract]) OR ("clinical trials as topic" [mesh]) OR (randomly [tiab] OR trial [ti]) NOT (animals [mh] NOT humans [mh])

#1 AND (#2 OR #3 OR #4 OR #5 OR #6 OR #7 OR #8 OR #9 OR #10) AND #11

**Web of Science**

#1

TS=(Spondylitis, Ankylosing OR Bechterew's Disease OR Bechterews Disease OR Marie-Struempell Disease OR Marie Struempell Disease OR Spondylarthritis Ankylopoietica OR Rheumatoid Spondylitis OR Spondylitis, Rheumatoid OR Ankylosing Spondylitis OR Ankylosing Spondylarthritis OR Ankylosing Spondylarthritides OR Spondylarthritides, Ankylosing OR Spondylarthritis, Ankylosing OR Ankylosing Spondyloarthritis OR Ankylosing Spondyloarthritides OR Spondyloarthritides, Ankylosing OR Spondyloarthritis, Ankylosing OR Spondylitis Ankylopoietica OR Bechterew Disease OR Spondylarthritis Ankylopoietica)

#2

TS=((Exercise Therapy OR Rehabilitation Exercise OR Exercise, Rehabilitation OR Exercises, Rehabilitation OR Rehabilitation Exercises OR Therapy, Exercise OR Exercise Therapies OR Therapies, Exercise OR Remedial Exercise OR Exercise, Remedial OR Exercises, Remedial OR Remedial Exercises) OR (Muscle Stretching Exercises OR Exercise, Muscle Stretching OR Muscle Stretching Exercise OR Active Stretching OR Stretching, Active OR Static-Active Stretching OR Static Active Stretching OR Stretching, Static-Active OR Proprioceptive Neuromuscular Facilitation (PNF) Stretching OR Proprioceptive Neuromuscular Facilitation OR Neuromuscular Facilitation, Proprioceptive OR Proprioceptive Neuromuscular Facilitations OR PNF Stretching OR PNF Stretchings OR Stretching, PNF OR PNF Stretching Exercise OR Exercise, PNF Stretching OR PNF Stretching Exercises OR Stretching Exercise, PNF OR Passive Stretching OR Stretching, Passive OR Static-Passive Stretching OR Static Passive Stretching OR Stretching, Static-Passive OR Relaxed Stretching OR Stretching, Relaxed OR Static Stretching OR Stretching, Static OR Isometric Stretching OR Stretching, Isometric OR Dynamic Stretching OR Stretching, Dynamic OR Ballistic Stretching OR Stretching, Ballistic) OR (Aquatic Therapy OR Hydrotherapy OR Therapy, Aquatic OR Pool Therapy OR Therapy, Pool OR Ai Chi Therapy OR Therapies, Ai Chi OR Therapy, Ai Chi OR Water Tai Chi Therapy OR Aquatic Exercise Therapy OR Exercise Therapy, Aquatic OR Therapy, Aquatic Exercise OR Water Exercise Therapy OR Exercise Therapy, Water OR Therapy, Water Exercise OR Water-based Exercise OR Aqua) OR (Resistance Training OR Training, Resistance OR Strength Training OR Training, Strength OR Weight-Lifting Strengthening Program OR Strengthening Programs, Weight-Lifting OR Strengthening Program, Weight-Lifting OR Weight Lifting Strengthening Program OR Weight-Lifting Strengthening Programs OR Weight-Lifting Exercise Program OR Exercise Programs, Weight-Lifting OR Exercise Program, Weight-Lifting OR Weight Lifting Exercise Program OR Weight-Lifting Exercise Programs OR Weight-Bearing Strengthening Program OR Strengthening Programs, Weight-Bearing OR Strengthening Program, Weight-Bearing OR Weight bearing Strengthening Program OR Weight-Bearing Strengthening Programs OR Weight-Bearing Exercise Program OR Exercise Programs, Weight-Bearing OR Exercise Program, Weight-Bearing OR Weight bearing Exercise Program OR Weight-Bearing Exercise Programs OR Muscle Strength Training) OR ("Yoga" OR "Tai Ji" OR Tai-ji OR Tai Chi OR Chi, Tai OR Tai Ji Quan OR Ji Quan, Tai OR Quan, Tai Ji OR Taiji OR Taijiquan OR "T'ai Chi" OR Tai Chi Chuan OR "Qigong" OR Qi Gong OR Ch'i Kung OR "Exercise Movement Techniques" OR Movement Techniques, Exercise OR Exercise Movement Technics OR Pilates-Based Exercises OR Exercises, Pilates-Based OR Pilates Based Exercises OR Pilates Training OR Training, Pilates OR McKenzie method OR Heckscher training OR running OR jogging OR exergame OR Swiss balls) OR ("Breathing Exercises" OR Inspiratory muscle training OR Expiratory muscle training OR Exercise, Breathing OR Respiratory Muscle Training OR Muscle Training, Respiratory OR Training, Respiratory Muscle) OR ("Exercise" OR "High-Intensity Interval Training" OR High Intensity Interval Training OR High-Intensity Interval Trainings OR Interval Training, High-Intensity OR Interval Trainings, High-Intensity OR Training) OR (balance training OR balance trainings OR balance exercise OR balance exercises OR flexibility training OR flexibility trainings OR flexibility exercise OR flexibility exercises OR joint mobility training OR joint mobility trainings OR joint mobility exercise OR joint mobility exercises OR functional movement training OR functional movement trainings OR functional movement exercise OR functional movement exercises OR stability training OR stability trainings OR stability exercise OR stability exercises OR medical gymnastics))

#3

TS=(randomized controlled trial OR controlled clinical trial OR randomized OR placebo OR clinical trials as topic OR RCT)

#1 AND #2 AND #3

**Cochrane Library**

(Spondylitis, Ankylosing OR Bechterew's Disease OR Bechterews Disease OR Marie - Struempell Disease OR Marie Struempell Disease OR Spondylarthritis Ankylopoietica OR Rheumatoid Spondylitis OR Spondylitis, Rheumatoid OR Ankylosing Spondylitis OR Ankylosing Spondylarthritis OR Ankylosing Spondylarthritides OR Spondylarthritides, Ankylosing OR Spondylarthritis, Ankylosing OR Ankylosing Spondyloarthritis OR Ankylosing Spondyloarthritides OR Spondyloarthritides, Ankylosing OR Spondyloarthritis, Ankylosing OR Spondylitis Ankylopoietica OR Bechterew Disease OR Spondylarthritis Ankylopoietica) AND ((Exercise Therapy OR Rehabilitation Exercise OR Exercise, Rehabilitation OR Exercises, Rehabilitation OR Rehabilitation Exercises OR Therapy, Exercise OR Exercise Therapies OR Therapies, Exercise OR Remedial Exercise OR Exercise, Remedial OR Exercises, Remedial OR Remedial Exercises) OR (Muscle Stretching Exercises OR Exercise, Muscle Stretching OR Muscle Stretching Exercise OR Active Stretching OR Stretching, Active OR Static - Active Stretching OR Static Active Stretching OR Stretching, Static - Active OR Proprioceptive Neuromuscular Facilitation (PNF) Stretching OR Proprioceptive Neuromuscular Facilitation OR Neuromuscular Facilitation, Proprioceptive OR Proprioceptive Neuromuscular Facilitations OR PNF Stretching OR PNF Stretchings OR Stretching, PNF OR PNF Stretching Exercise OR Exercise, PNF Stretching OR PNF Stretching Exercises OR Stretching Exercise, PNF OR Passive Stretching OR Stretching, Passive OR Static - Passive Stretching OR Static Passive Stretching OR Stretching, Static - Passive OR Relaxed Stretching OR Stretching, Relaxed OR Static Stretching OR Stretching, Static OR Isometric Stretching OR Stretching, Isometric OR Dynamic Stretching OR Stretching, Dynamic OR Ballistic Stretching OR Stretching, Ballistic) OR (Aquatic Therapy OR Hydrotherapy OR Therapy, Aquatic OR Pool Therapy OR Therapy, Pool OR Ai Chi Therapy OR Therapies, Ai Chi OR Therapy, Ai Chi OR Water Tai Chi Therapy OR Aquatic Exercise Therapy OR Exercise Therapy, Aquatic OR Therapy, Aquatic Exercise OR Water Exercise Therapy OR Exercise Therapy, Water OR Therapy, Water Exercise OR Water - based Exercise OR Aqua) OR (Resistance Training OR Training, Resistance OR Strength Training OR Training, Strength OR Weight - Lifting Strengthening Program OR Strengthening Programs, Weight - Lifting OR Strengthening Program, Weight - Lifting OR Weight Lifting Strengthening Program OR Weight - Lifting Strengthening Programs OR Weight - Lifting Exercise Program OR Exercise Programs, Weight - Lifting OR Exercise Program, Weight - Lifting OR Weight Lifting Exercise Program OR Weight - Lifting Exercise Programs OR Weight - Bearing Strengthening Program OR Strengthening Programs, Weight - Bearing OR Strengthening Program, Weight - Bearing OR Weight bearing Strengthening Program OR Weight - Bearing Strengthening Programs OR Weight - Bearing Exercise Program OR Exercise Programs, Weight - Bearing OR Exercise Program, Weight - Bearing OR Weight bearing Exercise Program OR Weight - Bearing Exercise Programs OR Muscle Strength Training) OR ("Yoga" OR "Tai Ji" OR Tai - ji OR Tai Chi OR Chi, Tai OR Tai Ji Quan OR Ji Quan, Tai OR Quan, Tai Ji OR Taiji OR Taijiquan OR "T'ai Chi" OR Tai Chi Chuan OR "Qigong" OR Qi Gong OR Ch'i Kung OR "Exercise Movement Techniques" OR Movement Techniques, Exercise OR Exercise Movement Technics OR Pilates - Based Exercises OR Exercises, Pilates - Based OR Pilates Based Exercises OR Pilates Training OR Training, Pilates OR McKenzie method OR Heckscher training OR running OR jogging OR exergame OR Swiss balls) OR ("Breathing Exercises" OR Inspiratory muscle training OR Expiratory muscle training OR Exercise, Breathing OR Respiratory Muscle Training OR Muscle Training, Respiratory OR Training, Respiratory Muscle) OR ("Exercise" OR "High - Intensity Interval Training" OR High Intensity Interval Training OR High - Intensity Interval Trainings OR Interval Training, High - Intensity OR Interval Trainings, High - Intensity OR Training) OR (balance training OR balance trainings OR balance exercise OR balance exercises OR flexibility training OR flexibility trainings OR flexibility exercise OR flexibility exercises OR joint mobility training OR joint mobility trainings OR joint mobility exercise OR joint mobility exercises OR functional movement training OR functional movement trainings OR functional movement exercise OR functional movement exercises OR stability training OR stability trainings OR stability exercise OR stability exercises OR medical gymnastics)) AND (randomized controlled trial OR controlled clinical trial OR randomized OR placebo OR clinical trials as topic OR RCT)

**Embase**

#1

"bechterews disease*":ab,ti,kw OR "marie-struempell disease*":ab,ti,kw OR "marie struempell disease*":ab,ti,kw OR "rheumatoid spondylitis*":ab,ti,kw OR "spondylitis, rheumatoid*":ab,ti,kw OR "ankylosing spondylitis*":ab,ti,kw OR "ankylosing spondylarthritis*":ab,ti,kw OR "ankylosing spondylarthritides*":ab,ti,kw OR "spondylarthritides, ankylosing*":ab,ti,kw OR "spondylarthritis, ankylosing*":ab,ti,kw OR "ankylosing spondyloarthritis*":ab,ti,kw OR "ankylosing spondyloarthritides*":ab,ti,kw OR "spondyloarthritides, ankylosing*":ab,ti,kw OR "spondyloarthritis, ankylosing*":ab,ti,kw OR "spondylitis ankylopoietica*":ab,ti,kw OR "bechterew disease*":ab,ti,kw OR "spondylarthritis ankylopoietica*":ab,ti,kw

##2

"rehabilitation exercise*":ab,ti,kw OR "rehabilitation exercises":ab,ti,kw OR "exercises, rehabilitation":ab,ti,kw OR "exercises rehabilitation":ab,ti,kw OR "rehabilitation therapies":ab,ti,kw OR "therapies, exercise":ab,ti,kw OR "exercise therapy*":ab,ti,kw OR "exercise therapies":ab,ti,kw OR "remedial exercise*":ab,ti,kw OR "remedial exercises":ab,ti,kw OR "exercise, remedial":ab,ti,kw OR "exercises, remedial":ab,ti,kw OR "muscle stretching exercise*":ab,ti,kw OR "muscle stretching exercises":ab,ti,kw OR "exercise, muscle stretching":ab,ti,kw OR "active stretching":ab,ti,kw OR "stretching, active":ab,ti,kw OR "static-active stretching":ab,ti,kw OR "static active stretching":ab,ti,kw OR "stretching, static-active":ab,ti,kw OR "proprioceptive neuromuscular facilitation (PNF) stretching":ab,ti,kw OR "proprioceptive neuromuscular facilitation":ab,ti,kw OR "neuromuscular facilitation, proprioceptive":ab,ti,kw OR "proprioceptive neuromuscular facilitations":ab,ti,kw OR "PNF stretching":ab,ti,kw OR "PNF stretchings":ab,ti,kw OR "stretching, PNF":ab,ti,kw OR "PNF stretching exercise":ab,ti,kw OR "exercise, PNF stretching":ab,ti,kw OR "PNF stretching exercises":ab,ti,kw OR "stretching exercise, PNF":ab,ti,kw OR "passive stretching":ab,ti,kw OR "stretching, passive":ab,ti,kw OR "static-passive stretching":ab,ti,kw OR "static passive stretching":ab,ti,kw OR "stretching, static-passive":ab,ti,kw OR "relaxed stretching":ab,ti,kw OR "stretching, relaxed":ab,ti,kw OR "static stretching":ab,ti,kw OR "stretching, static":ab,ti,kw OR "isometric stretching":ab,ti,kw OR "stretching, isometric":ab,ti,kw OR "dynamic stretching":ab,ti,kw OR "stretching, dynamic":ab,ti,kw OR "ballistic stretching":ab,ti,kw OR "stretching, ballistic":ab,ti,kw OR "aquatic therapy":ab,ti,kw OR "hydrotherapy":ab,ti,kw OR "therapy, aquatic":ab,ti,kw OR "pool therapy":ab,ti,kw OR "therapy, pool":ab,ti,kw OR "ai chi therapy":ab,ti,kw OR "therapies, ai chi":ab,ti,kw OR "therapy, ai chi":ab,ti,kw OR "water tai chi therapy":ab,ti,kw OR "aquatic exercise therapy":ab,ti,kw OR "exercise therapy, aquatic":ab,ti,kw OR "therapy, aquatic exercise":ab,ti,kw OR "water exercise therapy":ab,ti,kw OR "exercise therapy, water":ab,ti,kw OR "therapy, water exercise":ab,ti,kw OR "water-based exercise":ab,ti,kw OR "aqua":ab,ti,kw OR "resistance training":ab,ti,kw OR "training, resistance":ab,ti,kw OR "strength training":ab,ti,kw OR "training, strength":ab,ti,kw OR "weight-lifting strengthening program*":ab,ti,kw OR "strengthening programs, weight-lifting":ab,ti,kw OR "strengthening program, weight-lifting":ab,ti,kw OR "weight lifting strengthening program":ab,ti,kw OR "weight-lifting strengthening programs":ab,ti,kw OR "weight-lifting exercise program*":ab,ti,kw OR "exercise programs, weight-lifting":ab,ti,kw OR "exercise program, weight-lifting":ab,ti,kw OR "weight lifting exercise program":ab,ti,kw OR "weight-lifting exercise programs":ab,ti,kw OR "weight-bearing strengthening program*":ab,ti,kw OR "strengthening programs, weight-bearing":ab,ti,kw OR "strengthening program, weight-bearing":ab,ti,kw OR "weight bearing strengthening program":ab,ti,kw OR "weight-bearing strengthening programs":ab,ti,kw OR "weight-bearing exercise program*":ab,ti,kw OR "exercise programs, weight-bearing":ab,ti,kw OR "exercise program, weight-bearing":ab,ti,kw OR "weight bearing exercise program":ab,ti,kw OR "weight-bearing exercise programs":ab,ti,kw OR "muscle strength training":ab,ti,kw OR "yoga":ab,ti,kw OR "tai ji":ab,ti,kw OR "tai-ji":ab,ti,kw OR "tai chi":ab,ti,kw OR "chi, tai":ab,ti,kw OR "tai ji quan":ab,ti,kw OR "ji quan, tai":ab,ti,kw OR "quan, tai ji":ab,ti,kw OR "taiji":ab,ti,kw OR "taijiquan":ab,ti,kw OR "t'ai chi":ab,ti,kw OR "tai chi chuan":ab,ti,kw OR "qigong":ab,ti,kw OR "qi gong":ab,ti,kw OR "ch'i kung":ab,ti,kw OR "exercise movement techniques":ab,ti,kw OR "movement techniques, exercise":ab,ti,kw OR "exercise movement technics":ab,ti,kw OR "pilates-based exercises":ab,ti,kw OR "exercises, pilates-based":ab,ti,kw OR "pilates based exercises":ab,ti,kw OR "pilates training":ab,ti,kw OR "training, pilates":ab,ti,kw OR "mckenzie method":ab,ti,kw OR "heckscher training":ab,ti,kw OR "running":ab,ti,kw OR "jogging":ab,ti,kw OR "exergame":ab,ti,kw OR "swiss balls":ab,ti,kw OR "breathing exercises":ab,ti,kw OR "inspiratory muscle training":ab,ti,kw OR "expiratory muscle training":ab,ti,kw OR "exercise, breathing":ab,ti,kw OR "respiratory muscle training":ab,ti,kw OR "muscle training, respiratory":ab,ti,kw OR "training, respiratory muscle":ab,ti,kw OR "exercise":ab,ti,kw OR "high-intensity interval training":ab,ti,kw OR "high intensity interval training":ab,ti,kw OR "high-intensity interval trainings":ab,ti,kw OR "interval training, high-intensity":ab,ti,kw OR "interval trainings, high-intensity":ab,ti,kw OR "training":ab,ti,kw OR "balance training":ab,ti,kw OR "balance trainings":ab,ti,kw OR "balance exercise":ab,ti,kw OR "balance exercises":ab,ti,kw OR "flexibility training":ab,ti,kw OR "flexibility trainings":ab,ti,kw OR "flexibility exercise":ab,ti,kw OR "flexibility exercises":ab,ti,kw OR "joint mobility training":ab,ti,kw OR "joint mobility trainings":ab,ti,kw OR "joint mobility exercise":ab,ti,kw OR "joint mobility exercises":ab,ti,kw OR "functional movement training":ab,ti,kw OR "functional movement trainings":ab,ti,kw OR "functional movement exercise":ab,ti,kw OR "functional movement exercises":ab,ti,kw OR "stability training":ab,ti,kw OR "stability trainings":ab,ti,kw OR "stability exercise":ab,ti,kw OR "stability exercises":ab,ti,kw OR "medical gymnastics":ab,ti,kw

#3

randomized*:ab,ti,kw OR controlled clinical trial*:ab,ti,kw OR random*:ab,ti,kw OR placebo*:ab,ti,kw OR RCT:ab,ti,kw

#1 AND #2 AND #3

**Supplementary Methods 2 –** Conversion formula for standard deviation (SD).

**Standard Error to Standard Deviation:**

SD=SE×$\sqrt{N}$ where SE is the standard error and N is the sample size.

**95% Confidence Interval to Standard Deviation:**

1. For sample sizes in the test and control groups that are greater than or equal to 100, the standard deviation (SD) can be calculated as follows: SD = (Upper limit of credible intervals - Lower limit of credible intervals) / 3.92.
2. For sample sizes in the test and control groups that are less than or equal to 60, the standard deviation (SD) is determined by the formula: SD = (Upper limit of credible intervals - Lower limit of credible intervals) / tinv(1-0.95, n-1). 3. For studies where the sample sizes in each group range between 60 and 100, either of the aforementioned methods may be employed.

**Combined Subgroup**

In the combined subgroup analysis, let the sample size of subgroup A be denoted as N_1_, with a mean of M_1_ and a standard deviation of SD_1_. For subgroup B, let the sample size be N_2_, with a mean of M_2_ and a standard deviation of SD_2_. The total combined sample size is (N = N_1_ + N_2_). The combined mean M is calculated as (M = N_1_M_1_+ N_2_M_2_)/(N_1_ + N_2_).


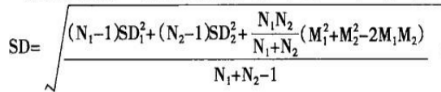


**Supplementary Table S1 Exercise Therapy Classification.**

| Modality | Defnition |
| --- | --- |
| Aerobic Exercise | Aerobic exercise constitutes a type of physical activity that enhances cardiovascular function by elevating both heart rate and respiratory rate. It typically involves sustained, moderate-intensity activities such as jogging, swimming, and cycling^1^. |
| Yoga | Yoga, a discipline rooted in ancient Indian traditions, encompasses a holistic approach to physical and mental development by integrating diverse practices, including asanas (body postures), pranayama (breath control), and dhyana (meditation). As a multifaceted method for relaxation and physical exercise, yoga has been extensively adopted to enhance muscular strength, flexibility, coordination of limbs, endurance, and the regulation of breathing, alongside fostering improved concentration^2^. |
| Qigong | Qigong is a traditional Chinese practice aimed at health preservation and cultivation, integrating physical movement, respiratory regulation, and mental concentration. It is regarded as a traditional plant-based biofeedback therapy that modulates physical and mental states through sustained physiological conditioning. In our study, the Eight Section Brocade, Yi Jin Jing, and Tai Chi are all classified within this category^3^. |
| Resistance Exercise | Resistance exercise encompasses exercises designed to enhance the strength, functionality, endurance, and hypertrophy of skeletal muscles^4^. |
| Stretching Exercise | Stretching exercises constitute a physical activity designed to enhance muscular flexibility and joint range of motion. These exercises are commonly employed as a preparatory activity prior to engaging in physical exercise and as a relaxation technique post-exercise, with the objectives of minimizing injury risk and facilitating muscle recovery. Stretching exercises can be categorized into several types, including static stretching, dynamic stretching, and proprioceptive neuromuscular facilitation (PNF) stretching^5-6^. |
| Hippotherapy Simulation | Equestrian sports help stabilize muscles and improve core strength, as well as enhance circulatory function, promote balance, and enhance overall health. To ensure safety, the equestrian simulator (HS) system is used to mimic the original movements of live horses^7-9^. |
| Pilates | Pilates, a prevalent exercise modality within the domains of rehabilitation and fitness, was developed by Joseph H. Pilates. Over a span of 50 years, he refined this method to encompass over 500 exercises focused on stretching and strengthening. The primary objective of Pilates is to enhance overall physical health by improving muscle strength, flexibility, and posture^10^. |

**References**

1.Powell KE, Paluch AE, Blair SN. Physical activity for health: what kind? How much? How intense? On top of what? Ann Rev Public Health. 2011;32:349–65.doi: 10.1146/annurev-publhealth-031210-101151.

2.Broderick J, Crumlish N, Waugh A, Vancampfort D. Yoga versus non-standard care for schizophrenia. Cochrane Database Syst Rev. 2017;9(9): CD012052.doi: 10.1002/14651858.CD012052.pub2.

3.Matos Luís Carlos,Sousa Cláudia Maria,Gonçalves Mário,et al: Qigong as a Traditional Vegetative Biofeedback Therapy: Long-Term Conditioning of Physiological Mind-Body Effects. BIOMED RESEARCH INTERNATIONAL 2015

4.Powell KE, Paluch AE, Blair SN. Physical activity for health: what kind? How much? How intense? On top of what? Ann Rev Public Health. 2011;32:349–65.doi: 10.1146/annurev-publhealth-031210-101151.

5.Budini Francesco,Gallasch Eugen,Christova Monica,et al: One minute static stretch of plantar flexors transiently increases H reflex excitability and exerts no effect on corticospinal pathways. EXPERIMENTAL PHYSIOLOGY 2017

6.Behm David G,Kay Anthony D,Trajano Gabriel S,et al: Mechanisms underlying performance impairments following prolonged static stretching without a comprehensive warm-up. EUROPEAN JOURNAL OF APPLIED PHYSIOLOGY 2020

7.terba JA, Rogers BT, France AP, et al. Horseback riding in children with cerebral palsy: effect on gross motor function. Dev Med Child Neurol. 2002;44(5):301–308.

doi: 10.1111/j.1469-8749.2002.tb00815.x.

8.Park ES, Rha DW, Shin JS, et al. Effects of hippotherapy on gross motor function and functional performance of children with cerebral palsy. Yonsei Med J.

2014;55(6):1736–1742. doi: 10.3349/ymj.2014.55.6.1736.

9.[Salbaş](https://pubmed.ncbi.nlm.nih.gov/?term=Salba%C5%9F+E&cauthor_id=37643349) E, [Karahan](https://pubmed.ncbi.nlm.nih.gov/?term=Karahan+AY&cauthor_id=37643349) AY.The effectiveness of hippotherapy simulation exercises for muscle strength, disease activity and quality of life in sedentary adults with ankylosing spondylitis.Ann Med. 2023;55(2):2249822. doi: 10.1080/07853890.2023.2249822.

1. Franks Jennifer,Thwaites Claire,Morris Meg E : Pilates to Improve Core Muscle Activation in Chronic Low Back Pain: A Systematic Review. HEALTHCARE 2023

**Supplementary Table S2 Explanation of technical terms.**

| **Standardized mean difference (SMD)** | The SMD serves as an effect size metric employed to quantify the mean difference between two data groups. By normalizing the mean difference between groups through division by the pooled standard deviation, it mitigates the influence of dimensionality, thereby facilitating the comparability of effect sizes across diverse studies or variables. This standardization enables the translation of differences into a common metric, allowing for the comparison of studies conducted on varying scales or units. |
| --- | --- |
| **95% credible intervals (CI)** | In the context of frequentism metwork meta-analysis, the 95% confidence interval (CI) serves as a fundamental tool for evaluating the uncertainty associated with the effects of interventions. This approach is grounded in classical statistical theory, which posits that the effect size is a fixed parameter. The CI quantifies the "true effect" by synthesizing both direct and indirect comparison evidence, thereby delineating the potential range of effect sizes. Its application spans several critical scenarios: it can quantify the impact of intervention measures (such as risk ratios and mean differences) and determine statistical significance by assessing whether the CI includes null values (for instance, whether the CI encompasses 0 for continuous variables or 1 for binary variables). Furthermore, the CI can assess the reliability of evidence by examining the extent of overlap between direct and indirect evidence in consistency tests. Unlike the credible interval used in Bayesian network meta-analysis, the 95% CI in this context is fundamentally an inference about true values under repeated sampling, emphasizing objective evidence inference. It provides a rigorous statistical framework for integrating complex evidence networks and supports evidence-based decision-making. |
| **Statistical difference** | In the context of frequentism metwork meta-analysis, the determination of statistical significance is grounded in classical statistical theory. The primary objective is to assess whether the observed differences between interventions are "non-random" by examining the effect size and its corresponding 95% confidence interval (CI). For relative effect measures, such as the hazard ratio (HR) or odds ratio (OR), a 95% CI that does not encompass the null value of 1 indicates a statistically significant difference. Similarly, for absolute effect measures, such as the mean difference (MD) or standardized mean difference (SMD), a 95% CI that does not include 0 signifies statistical significance. This evaluative criterion is applied consistently, regardless of whether the comparison is a direct head-to-head test or an indirect comparison facilitated through an intermediate intervention. |
| **Heterogeneity** | In the context of meta-analysis, heterogeneity pertains to the variability among included studies, which can manifest across various dimensions such as study outcomes, methodologies, population characteristics, or intervention strategies. This variability fundamentally indicates that the effect size of each individual study cannot be adequately accounted for by a single common effect. The concept of heterogeneity encompasses statistical, clinical, and methodological dimensions. Statistical heterogeneity is assessed by quantifying the degree of variation in effect sizes, with the I² statistic frequently employed as a measure; for instance, an I² value exceeding 50% is indicative of moderate to high heterogeneity. |
| **SUCRA (Surface Under the Cumulative RANK Curve)** | In the context of network meta-analysis, SUCRA (Surface Under the Cumulative RANK Curve) serves as a metric to assess the likelihood of ranking the relative efficacy of various interventions. This measure effectively consolidates the ranking probabilities of different interventions into a single value ranging from 0 to 1, facilitating intuitive comparison and ranking. A SUCRA value approaching 1 suggests that an intervention is more likely to be effective, whereas a value nearing 0 indicates a higher likelihood of being the least effective treatment. By weighting the cumulative probabilities of all potential ranking positions, SUCRA synthesizes evidence from both direct and indirect comparisons, thereby overcoming the limitations associated with rankings based solely on point estimates. Additionally, the 95% confidence interval provides insight into the stability of these rankings. As a non-parametric measure, SUCRA does not require assumptions regarding the distribution of effect sizes. In the context of network comparisons involving multiple interventions, SUCRA offers a quantitative basis for clinical decision-making, aiding researchers and policymakers in identifying the most favorable interventions. |

# **Supplementary TableS****3 Demographic characteristics of included studies.**

This supplementary file shows the Demographic characteristics of included studies. The study indicates the name of the author and the year of publication. The exact dose parameter indicates the exact estimated METs per week that participants accumulated in the study. The dose indicates the group of doses by approximation. The residual dose indicates the difference between the exact dose and the dose allocated by approximation. The frequency is the number of days those participants were involved in physical activity. CT:Conventional Therapy; AE:Aerobic Exercises; RE: Resistance Exercise; PT:Physical Therapy; SE:Stretching Exercise; HS:Hippotherapy Simulation; UK: the United Kingdom; USA: the United States of America; NA: Not Available; BASFI:Bath Ankylosing Spondylitis Functional Index; BASDAI:Bath Ankylosing Spondylitis Disease Activity Index; BASMI:Bath Ankylosing Spondylitis Metrology Index; CE:Chest Expansion .

| Study | | Interventions/control,  sample size (male) | Age  (mean±sd) | BMI  (mean±sd) | Intervention  duration  (weeks) | Frequency | Time  /session | Exact  dose | Disease  duration (years) | Region | Biological Agents  Treatment | Outcome |
| --- | --- | --- | --- | --- | --- | --- | --- | --- | --- | --- | --- | --- |
| Wang2022 | | CT=28(21) | 33.2±6.2 | 23.8±3.3 | 16 | NA | NA | 0 | 10.1±6.1 | China | No | ①②③ |
|  | | AE=26(20) | 31.2±6.3 | 22.4±2.8 | 16 | 5 | 30 | 1050 | 10.9±5.5 |  |  |  |
| Acar2023 | | CT=27(16) | 45.33±7.24 | 26.76±3.04 | 8 | NA | NA | 0 | 13.63±7.4 | Turkey | No | ①② |
|  | | Yoga=28(15) | 44.14±8.03 | 27.2±4.47 | 8 | 3 | 60 | 450 | 13.93±7.8 |  |  |  |
| Xie2019 | | NI=23(18) | NA | NA | 12 | NA | NA | 0 | NA | China | No | ①②③④ |
|  | | Baduanjin=23(17) | NA | NA | 12 | 2-3 | 20 | 225 | NA |  |  |  |
| Souza2017 | | CT=30(21) | 43.8±10.2 | NA | 16 | NA | NA | 0 | 9.6±7.8 | Brazil | No | ①②③④ |
|  | | RE=30(23) | 45±9.8 | NA | 16 | 2 | 50 | 600 | 8.8±6.6 |  |  |  |
| Salbaş2023 | | AE+SE=22(14) | 35.5±7.3 | 27.6±5.1 | 12 | NA | NA | 630 | NA | Turkey | Anti-TNF | ①②③ |
|  | | HS=20(13) | 34.4±7.3 | 28.4±4.5 | 12 | 4 | 40 | 910 | NA |  |  |  |
| Altan2006 | | AE=30(NA) | NA | NA | 24 | 7 | 30 | 1200 | NA | Turkey | No | ①③④ |
|  | | AE+PT=30(NA) | NA | NA | 24 | 7 | 30 | 1200 |  |  |  |  |
| Gencer2024 | | AE=30(9) | 49.53±10.2 | NA | 12 | 2 | 25 | 315 | 10±6.49 | Turkey | No | ①③④ |
|  | | Baduanjin=29(8) | 46.32±9.74 | NA | 12 | 2 | 25 | 210 | 8.21±4.84 |  |  |  |
| Cetin2020 | | AE=18(10) | 44.66±8.02 | 30.66±8.65 | 10 | 2 | 30 | 456 | 7.42±5.27 | Turkey | No | ①②③ |
|  | | Taiji=18(9) | 46.88±10.46 | 34.93±8.02 | 10 | 2 | 30 | 180 | 8.83±4.69 |  |  |  |
| Kjeken2013 | | CT=49(26) | 48.6±9.4 | 33.6±5.2 | 12 | NA | NA | 0 | 16.1±12 | Norway | No | ①②③ |
|  | | AE+SE+Supervise=46(36) | 49.4±10.3 | 49±9.8 | 12 | 3 | 90-121 | 2452 | 14.9±9.6 |  |  |  |
| Analay2003 | | AE+SE=22(18) | NA | NA | 6 | 3 | 50 | 857 | NA | Turkey | No | ①④ |
|  | | AE+SE+Supervise=23(20) | NA | NA | 6 | 3 | 50 | 857 | NA |  |  |  |
| Calik2021 | | SE=14(4) | 42.85±11.07 | 27.6±6.08 | 12 | 3 | 30 | 315 | NA | Turkey | Anti-TNF | ①②③ |
|  | | AE+SE=17(8) | 46.58±11.94 | 30.75±4.51 | 12 | 3 | 60 | 1215 | NA |  |  |  |
| Karahan2016 | | CT=29(23) | 36.6±11.3 | 28.2±3.2 | 8 | NA | NA | 0 | NA | Turkey | Anti-TNF | ①③ |
|  | | AE=28(24) | 36.1±12.4 | 27.7±2.1 | 8 | 5 | 30 | 825 | NA |  |  |  |
| Sweeney2002 | CT=80(53) | | 45.9 | 31.3±4.2 | 16 | 3 | 30 | 585 | NA | UK | No | ①③ |
|  | AE=75(51) | | 46.5 | 33.9±5.4 | 16 | 3 | 30 | 540 | NA |  |  |  |
| Ayhan2011 | | AE=31(20) | 37±9.6 | 33.2±4.1 | 3 | 3 | NA | 405 | 7.4±7.7 | Turkey | No | ①③ |
|  | | AE+PT=29(24) | 42.5±10.6 | 33.5±4.7 | 3 | 3 | NA | 1050 | 9.9±7.7 |  |  |  |
| Acar2023 | | AE+SE=21(13) | 41.8±8.6 | 26.6±3.7 | 8 | 3 | 30 | 315 | 9.4±9 | Turkey | No | ①②③ |
|  | | Pilates=21(11) | 43.9±8.9 | 27.4±3.7 | 8 | 3 | 40 | 360 | 11.3±7.7 |  |  |  |
| Masiero2014 | | CT=21(17) | 46.15±10.3 | NA | 6 | 2 | NA | 0 | 9.15±4.23 | Italy | Anti-TNF | ①②③④ |
|  | | AE+SE+Supervise=21(16) | 49.11±11.8 | NA | 6 | 2 | 60 | 660 | 9.11±6.9 |  |  |  |
| Aksoy2017 | | AE+SE=21(17) | 37.47±11.09 | NA | 1 | 4 | 40 | 700 | NA | Turkey | No | ①②③④ |
|  | | AE+SE+Supervise=20(15) | 37.95±9.84 | NA | 1 | 4 | 40 | 700 | NA |  |  |  |
| Jennings2015 | | SE=35(23) | 40.2±9.3 | 25.86±4 | 12 | 3 | 30 | 315 | 13.4±7.8 | Brazil | Anti-TNF | ①②③④ |
|  | | AE+SE=35(26) | 42.9±9.9 | 26.69±4.58 | 12 | 3 | 70 | 735 | 16±8.9 |  |  |  |
| Oksüz2023 | | AE=13(11) | 41.7±12.5 | 24.2±4 | 8 | 3 | 30 | 236 | 10.8±7.4 | Turkey | No | ①②③ |
|  | | AE+Pilates=13(7) | 46.2±11.6 | 26.9±6.7 | 8 | 3 | ＞30 | 506 | 11.8±8.7 |  |  |  |
| Aydın2016 | | AE+SE=19(11) | 33.52±7.72 | NA | 8 | 5 | 20 | 302 | 7.1±2.79 | Turkey | Anti-TNF | ①②③④ |
|  | | AE+SE+Supervise=18(9) | 35.83±8.08 | NA | 8 | 5 | 20 | 302 | 7.22±2.19 |  |  |  |
| Karapolat2009 | | SE=12(9) | 48.42±9.47 | 24.3±3.78 | 6 | 6 | 30 | 630 | 18.63±7.52 | Turkey | No | ①②③④ |
|  | | AE+SE=13(10) | 50.15±12.4 | 25.81±4.11 | 6 | 3-6 | 30-60 | 1152 | 20.62±10.1 |  |  |  |
| Roşu2014 | | AE=48(40) | 24.98±3.83 | NA | 48 | 3 | 20 | 210 | 5.35±3.11 | Romania | Yes | ①②③④ |
|  | | AE+Pilates=48(39) | 25.33±3.77 | NA | 48 | 3 | 50 | 495 | 5.81±3.02 |  |  |  |
| Gurcay2008 | | AE=28(22) | 41.3±8.59 | NA | 3 | 5 | 30 | 525 | 13.53±9.33 | Turkey | No | ①②③ |
|  | | AE+PT=29(27) | 40.2±10.38 | NA | 3 | 5 | 30 | 525 | 16.21±10.22 |  |  |  |
| Altan2012 | | AE=24(NA) | 43.6±10.1 | NA | 12 | 0 | 0 | 0 | NA | Turkey | No | ①②③④ |
|  | | Pilates=29(NA) | 46.5±11.2 | NA | 12 | 3 | 60 | 540 | NA |  |  |  |
| Gandomi2022 | | CT=14(NA) | 38.07±8.69 | 26.48±3.42 | 6 | 0 | 0 | 0 | 7.42±4.89 | Iran | Anti-TNF | ① |
|  | | SE=14(NA) | 39.21±10.25 | 25.53±2.76 | 6 | 4 | 60 | 700 | 10.42±10.76 |  |  |  |
|  | | Pilates=12(NA) | 42.58±14.18 | 28.2±3.73 | 6 | 4 | 60 | 1100 | 11.25±7.6 |  |  |  |
| Singh2023 | | CT=52(43) | 35.09±9.86 | NA | 12 | 3 | 0 | 0 | NA | India | No | ①③ |
|  | | Yoga=57(48) | 34.42±9.39 | NA | 12 |  | 60 | 300 | NA |  |  |  |
| Hsieh2014 | | SE=10(7) | 42.1±8.8 | 24.7±3.7 | 12 | 3 | 25-35 | 360 | 17.3±10.7 | Taiwan, China | No | ①③④ |
|  | | AE+SE=9(6) | 36.2±11.7 | 23.8±7.8 | 12 | 2-3 | 25-35 | 2550 | 11.1±6.8 |  |  |  |
| Ciprian2013 | | CT=15(14) | 45.6±11.8 | NA | 2 | 0 | 0 | 1206 | 13.2±8.8 | Italy | Anti-TNF | ②③ |
|  | | AE+PT=15(14) | 47.8±10 | NA | 2 | 5 | 1650 | 1080 | 13.9±8.6 |  |  |  |
| Dundar2014 | | SE=34(6) | 43.1±11.7 | 29.7±2.7 | 4 | 5 | 60 | 1225 | 14.1±12.2 | Turkey | No | ②③④ |
|  | | AE+SE=35(5) | 42.3±11.3 | 29.6±2.4 | 4 | 5 | 60 | 1100 | 13.7±12.5 |  |  |  |
| Lee2008 | | NI=17(15) | 34.9±12.9 | 32.1±3.2 | 8 | 0 | 0 | 0 | 2.7±1.1 | South Korea | No | ③ |
|  | | Taiji=13(10) | 35.2±11.5 | 32.0±3.4 | 8 | 1-2 | 30 | 630 | 2.9±1 |  |  |  |
| Soufivand2024 | | CT=23(NA) | 38.07±8.69 | 26.48±3.48 | 6 | 0 | 0 | 0 | NA | Iran | Anti-TNF | ③④ |
|  | | SE=24(NA) | 39.21±10.25 | 25.53±2.76 | 6 | 4 | 35-45 | 825 | NA |  |  |  |
|  | | Pilates=29(NA) | 42.58±14.18 | 28.2±3.73 | 6 | 4 | 45 | 720 | NA |  |  |  |
| Ince2006 | | CT=15(9) | 36.13±7.2 | NA | 12 | 3 | 0 | 0 | 9.79±6.46 | Turkey | No | ④ |
|  | | AE+SE=15(9) | 33.67±5.15 | NA | 12 | 3 | 35 | 654 | 8.27±5.71 |  |  |  |

①BASFI;②BASMI;③BASDAI;④CE

# **Supplementary TableS4 Details of interventions conducted in the trial.**

| **Study** | **Group 1** | **Group 2** | **Group 3** |
| --- | --- | --- | --- |
| **Acar2023** | Participants used the Zoom videoconferencing program to attend remote yoga classes 3 times a week for 8 weeks, with a maximum of 5 people in each group. Each yoga class lasted 60 min on average and included warm-up (loosening exercises, Surya Namaskar A or B), vinyasa flow (asana sequence), pranayama, deep relaxation (savasana), and meditation | Participants in the control group were asked to continue their routine activities and not to begin any other  exercise program for the following 2 months. After the study was completed, participants in the control group were given the opportunity to participate in the tele-yoga program. | NA |
| **Acar2023** | The Pilates group was divided into 3 subgroups (10 participants each) for the training. Participants were taught about the five key elements (breathing, centering, ribcage placement, shoulder blade placement, head-neck placement) in one session before the training. Pilates training sessions lasting 60 min each were held 3 times a week for 8 weeks. Each session consisted of a 5-min warm-up, 5-min key elements, 40-min main program, and 10- min cool-down. | Home-based exercises were demonstrated by a physiotherapist  and brochures were given to the patients in the control group. The home-based exercise program included spinal ROM exercises; trunk flexibility exercises; strengthening exercises for the abdominal, back, hip, and shoulder muscles; stretching exercises for the  major muscle groups (trapezius, hamstring, and pectoral muscles; hip flexor and adductor muscles); and posture exercises. Patients were asked to follow this exercise program at home individually for at least 30 min per session 3 times a week for 8 weeks and continue their normal daily activities. An exercise diary was used to monitor compliance with home exercise. After the 8 weeks, the Pilates exercise intervention was also offered to this group. | NA |
| **Aksoy2017** | A structured education and exercise program, spanning five days, was implemented for the first cohort of patients (Group 1). Each cohort comprised 4-5 patients who engaged in activities such as stretching and deep breathing exercises under the supervision of a physical therapist. These patients participated in small group sessions throughout the educational program, exercising for 40 minutes daily. | The second cohort adhered to routine clinical care, where the physiotherapist prescribed necessary exercises as part of a family plan. These exercises encompassed dexterity, stretching, deep breathing, spinal stretching, and range of motion exercises targeting the spine, shoulders, and hips. Participants in this group were instructed to continue these exercises four times. | NA |
| **Altan2006** | In the first cohort, patients participated in bath therapy at the outpatient clinic during the early morning hours. This involved immersion in a treatment pool containing hot spring water maintained at 39 ºC for a duration of 30 minutes, once daily, over a period of three weeks. Subsequently, they were instructed to engage in a 30-minute home exercise regimen each day following the bath, which comprised breathing and posture exercises as well as back and waist stretching exercises. | The second cohort adhered to the identical exercise regimen but did not partake in the bath therapy. | NA |
| **Altan2012** | In the first group, comprising 30 participants, individuals engaged in a structured Pilates exercise regimen administered by a qualified instructor for one hour, three times per week over a 12-week period. | The second group functioned as a control group, consisting of 25 participants who maintained their existing treatment protocol. Participants in the control group received standard care and were advised to persist with their customary physical activities. | NA |
| **Analay2023** | Patients in the first group participated in a physical therapy program overseen by a physical therapist. A total of 23 subjects engaged in an intensive exercise regimen over a six-week period, consisting of 50-minute sessions three times per week. The exercise program encompassed stretching, activities, and strengthening exercises targeting the lower limbs, upper limbs, and back, as well as aerobic exercises on stationary bicycles, and posture and breathing exercises. Initially, after achieving proper posture, the hip flexors and pectoral muscles were stretched, followed by the strengthening of the hip and back extensors, along with both upper and lower limbs. Subsequently, resistance-free exercises were performed according to each individual's tolerance level. | Patients in the second cohort were instructed to independently perform the same exercises as the first cohort at home over a six-week period, with a frequency of three days per week. They received weekly telephone follow-ups but did not benefit from in-person supervision. | NA |
| **Aydın2016** | The first group engaged in a structured exercise regimen comprising five sessions per week over an eight-week period. This regimen consisted of three days dedicated to aerobic exercises and two days allocated to relaxation exercises. The aerobic sessions emphasized large, rhythmically performed movements integrated with breathing techniques. These exercises were continuous and repetitive, targeting major muscle groups through aerobic movements and footwork. The sessions also incorporated a regional training sequence and concluded with a five-minute rest period. Aerobic activities were conducted three times weekly, each lasting one hour and comprising a 15-minute warm-up, 20 minutes of intensive training, 10 minutes of relaxation, and an additional 15 minutes of relaxation. The relaxation exercises were scheduled for 20 minutes, twice a week. All exercise sessions were conducted under the supervision of physiotherapists at the hospital, who provided in-person guidance to the participants. | The second group was the family group, which required exercise at home, and the exercise plan was consistent with that of the first group, and the exercise plan was tracked daily by phone, and the patients in this group did not have offline supervision. |  |
| **Ayhan2011** | The first group comprised the inpatient rehabilitation group, wherein patients diagnosed with ankylosing spondylitis underwent a treatment regimen beginning with the application of a hot pack to the spine for a duration of 10 minutes per session. This was followed by ultrasound therapy administered at an intensity of 0.5 watts/cm² for 6 minutes, also lasting 10 minutes per session. Subsequently, Faraday current was applied to the paravertebral region for an additional 10 minutes per session. The rehabilitation protocol included posture training, respiratory exercises, and endurance training specifically tailored for individuals with ankylosing spondylitis. Additionally, the regimen encompassed shoulder and hip range of motion (ROM) exercises, neck stretching, and ROM training. Trunk rotation, lateral flexion, cat stretching, and lower limb stretching exercises were also incorporated into the training program. Active range of motion exercises and quadriceps isometric strengthening protocols were implemented for hospitalized patients, organized into two groups with 20 sessions per day. Patients were instructed to engage in mirror reflection posture training and posture exercises. Breathing exercises, including deep breathing, abdominal breathing, air transfer, and pursed-lip breathing, were conducted for 15 minutes each session. Additionally, individualized walking endurance training was administered three times per week. The patient was discharged following a three-week in-hospital rehabilitation program. | Participants in the home exercise cohort were directed to execute the identical exercise regimen as previously detailed. They were encouraged to complete two sets of 20 sessions daily at home over a period of three weeks. Additionally, these participants were prescribed respiratory and endurance training exercises. It is important to note that participants in the home exercise group did not have the opportunity to consult with a physical therapist and were not subject to direct supervision. Adherence to the treatment protocol was verbally assessed every three months. | NA |
| **Calik2021** | The intervention group participated in a structured protocol that integrated aerobic exercise with spinal mobility exercises. Participants engaged in aerobic exercise on a treadmill for 40 minutes, maintaining an intensity of 55-80% of their maximum heart rate. Additionally, they performed 20 distinct spinal mobility exercises for approximately 30 minutes, aimed at enhancing spinal mobility and flexibility. These exercises targeted the flexibility of the cervical, thoracic, and lumbar regions of the spine, as well as shoulder range of motion. They included stretching exercises for the hamstrings, quadriceps, and erector spinae muscles, strengthening exercises for the abdominal, back, and proximal muscles, and exercises focused on diaphragmatic breathing and chest expansion. The entire program was conducted under the supervision of a physical therapist and was practiced three times per week over a 12-week period. | The control group engaged solely in spinal mobility exercises, with all other conditions mirroring those of the intervention group. | NA |
| **Cetin2020** | The Tai Chi regimen developed by the Tai Chi group for patients was derived from the 24 simplified forms of Yang Style and identified as the 10 fundamental forms of Tai Chi. Each session lasted for one hour, comprising 15 minutes of warm-up exercises, 30 minutes of Tai Chi practice, and 15 minutes of relaxation exercises. | the home exercise group engaged in a series of 20 exercises targeting cervical, thoracic, and lumbar flexibility; stretching of the peri-shoulder, hamstring, and erector spinae muscles; as well as exercises aimed at strengthening the abdominal, back, and proximal muscles. This regimen was conducted over 10 sessions, each lasting one hour, and was performed twice a week. | NA |
| **Ciprian2013** | Interventions in the spa-exercise group included, mud pack (40–45℃) applied to the entire spinal area for 15 min, followed by thermal bath (immersion to the  level of the neck in a tank of water at 37–38℃ for 10 min); group rehabilitation session performed for an hour in a pool of thermal water (32–34℃) under the supervision of a specialized physiotherapist. The session included exercises for spine mobilization (flex/extension and  torsion of the trunk), exercises for muscular spine strengthening and respiratory kinesitherapy (thoraco  pulmonary mobilization). | Patients in the control group continued with the original TNF inhibitor treatment without change. | NA |
| **Dundar2014** | Aquatic therapy group (Group I) participated under the supervision of a physiotherapist during the therapy program. Aquatic exercise program consisted of 20 sessions, 5× per week for 4 weeks in a swimming  pool at 32–33 °C. Each session was conducted in groups of 8–9 patients and lasted 60 min. The program started with 15 min of poolside exercises including warming up, active range of motion (ROM), and stretching. Poolside exercises were followed with 40 min of aquatic exercises in the pool including warming up (such as walking forwards and backwards in the pool); aerobic exercises (such as jumping, jogging); active ROM of the joints of the all extremities and stretching of neck, trunk and all extremities; strengthening (such as hip adduction and abduction, knee flexion and extension); straight posture, respiratory exercises, and  relaxation (such as lying supine). The program ended with cooling-down (such as slow walking and squatting) for 5 min. | Group II received a home-based exercise (land-based exercise) program for 4 weeks, performing  each exercise once a day with 15–20 repetitions lasting for 60 min. Exercise program including muscle relaxation (such as deep breathing, stretching, and relaxing of differ  ent muscle groups in the body, lying supine) for 10 min; flexibility exercises for cervical, thoracic, and lumbar spine (such as bringing chin to the chest, looking up to the ceiling, thoracic flexion and extension, lumbar flexion and extension) ROM exercises of hip joints, stretching exercises for the major muscle groups (such as stretching exercises for shoulder muscles, abdominal muscles, erector spine, hamstring, quadriceps, hip flexors, and stretching of neck, trunk and all extremities) for 30 min; straight posture, respiratory exercises, and muscular strengthening (such as  strengthening of muscle groups of upper and lower limbs and isometric exercise of the superficial trunk muscles) for 20 min | NA |
| **Gandomi2022** | As for Aqua‑based Stretch group , 24 Aqua Stretch exercise sessions were held (four sessions per week), lasting for six weeks. Exercises were performed in a swimming pool at 32–33°c, and each session lasted 60 min. The program started with 10–15 min of poolside exercises including warming-up (forward, backward, and sideward walking and jogging). Self-wall side passive stretching exercises, executed by noodles, were done for 30-min during the first two weeks, followed by self-wall side active stretching exercises were performed in 35-min sessions for the second two weeks, and in 45-min sessions for the third two weeks. Each stretching exercise lasted 15s,  30 s, and 45 s for the first, second, and third two weeks, respectively. Stretching exercises were performed in two series; the first series of stretches included stretching exercises performed on each joint . The second series of stretches was performed based on the pattern of muscle chains. The program ended  with cooling-down including slow walking and passive stretching for 5–15 min. | A total of 24 sessions of aqua-based Pilates exercises was performed (four sessions per week), lasting for six weeks. The exercises were performed in a swimming pool at 32–33 °C, and each session lasted 60 min. The program started with 10 min poolside  exercises including warming-up (five min of walking and five min of general active range of motion (ROM) and stretching). Poolside exercises were followed by 45 min of main exercises such as: breathing exercises, standing criss cross, T balance with noodle, standing spine stretch (3 set, 20 s hold), spine twists, standing single kicks (3 set, 10 repetition) for the first two weeks.  Breathing, standing spine stretch and single side leg lift, plank on a noodle, side kick, hip work, single leg stretch  (by cycling on a noodle), T balance exercises (3 set, 10 repetition) for the second two weeks. Finally, breathing and side kick exercises, side plank with one hand on a noodle, spine twist (3 set, 10 repetition), cat stretch  and T balance in water perturbation (3 set, 20 s hold). The program ended with cooling-down, including slow  walking and passive stretching for five min. | The control group patients continued  their usual activities and drug treatment (NSAIDs & Anti TNF). |
| **Gencer2024** | The intervention group performed a Baduanjin Qigong online exercise program (via Zoom video conference) for 12 weeks under the supervision of two physiotherapists. Baduanjin qigong exercise forms are  listed below. Baduanjin Qigong is characterized by slow and relaxing  movements.14 Baduanjin qigong exercises were performed two days a  week. Each session took 45 min (10 min for warming up exercises, 25 min for Baduanjin qigong exercises, and 10 min for cooling down ex  ercises). | The control group program in the study was created based on home based exercise programs applied in the literature for patients with  AS .The home exercise program included stretching for cervical,  thoracic, and lumbar flexibility, shoulder girdle, hamstring, and erector spinal muscles, and strengthening exercises for abdominal, back, and proximal muscles (a total of 20 exercises). Home exercises were per formed 45 min twice a week for 12 weeks. The exercises were taught to the patients by physiotherapists, and the accuracy of the exercises was checked whether they performed them once a week. | NA |
| **Gurcay2008** | The first group of patients participated in a structured exercise regimen, supplemented by a daily 20-minute session of Stanger's bath, over a period of three weeks, totaling 15 sessions. The exercise regimen encompassed activities designed to enhance range of motion, strengthen muscles, improve breathing, and correct posture. Patients were instructed to replicate this exercise program at home for 30 minutes per day, five days a week, for three weeks. | The second cohort of patients engaged in an identical exercise regimen but did not receive the Stanger's bath therapy. | NA |
| **Hsieh2014** | The COMB group received not only range-of-motion exercise, but also strengthening of the muscles of the major joints (including the cervical spine, thoracolumbar spine, shoulder, elbow, wrist, hip, knee, and ankle) and aerobic  exercise (including fast walking, cycling, and swimming as suggested). Each set of strengthening exercises consisted of 10 repetitions, and the intensity was set at 60% to 80% of one repetition maximum. Each subject was asked to perform two sets of strengthening exercises each time, 2 times per week. A rest interval between sets was 2 to 3 minutes.Aerobic  exercise program consisted of 5 min stretching of the exercise muscles, 5 min warm-up, 20–30 min aerobic exercise, and 5 min cooling-down. | Subjects in the ROM group received  instruction in range-of-motion exercise of the spine and major joints (including the shoulder, elbow, wrist, hip, knee, and ankle) from a senior physical therapist. Chest expansion and breathing exercise were also included. An exercise booklet was also given to each subject. After participants learned how to perform the range-of-motion exercise, they are instructed to conduct exercise at home daily for 3 months. Each range-of-motion exercise was repeated 5 times. | NA |
| **Ince2006** | The multi-exercise group underwent a regimen comprising aerobic, stretching, and pulmonary exercises. The aerobic component included a variety of movements such as marching (step), tap up-tap down (up and down step), V step, step touch (point step), turn step, grapevine, grapevine with knee lift, grapevine with leg curl, among eight other exercises. The intensity of these exercises was determined using the Karvonen formula, with a metronome and the Borg perceived exertion scale employed for monitoring, ensuring a low-intensity training level. The stretching component consisted of 14 exercises, which included head flexion and posterior flexion, head flexion, chest and shoulder stretch, deltoid stretch, triceps brachii stretch, overhead stretch, lateral trunk stretch, arched back stretch, leg extensor and pelvic flexor stretch, spinal torsion stretch, paravertebral muscle stretch, loosen-up stretch, upper back prayer, and knee-to-chest stretch. Lung exercises encompass various techniques such as nasal inhalation at twice the normal rate, standard nasal inhalation, thoracic and abdominal breathing, deep inhalation followed by oral exhalation, and inspiratory muscle resistance exercises, including hand pressure chest breathing. | The control group was administered pharmacological treatment for ankylosing spondylitis, consisting of nonsteroidal anti-inflammatory drugs (NSAIDs) and sulfasalazine at a dosage of 2 grams per day, without the incorporation of any supplementary exercise interventions. | NA |
| **Jennings2015** | The intervention group engaged in a regimen that combined aerobic training with stretching exercises. The aerobic component of the training consisted of a structured protocol: a 5-minute warm-up period, followed by a 40-minute walking session, and concluded with a 5-minute cool-down phase. The stretching exercises were specifically designed to target the trunk, upper limbs, and lower limbs, with each stretch being repeated three times for a duration of 30 seconds. | The control group participated solely in stretching exercise therapy. | NA |
| **Karahan2016** | The sports game group participated in a sports game therapy regimen, which encompassed simulations of football, table tennis, skiing, tennis, golf, volleyball, and bowling. Each participant engaged in 30-minute gaming sessions, totaling 40 sessions over a period of 5 days per week for 8 consecutive weeks. | The control group continued with conventional therapy, which did not include any exercise-based interventions. | NA |
| **Karapolat2009** | Participants in the routine exercise cohort engaged in a 30-minute session of standardized physical activity, which encompassed flexibility exercises targeting the cervical, thoracic, and lumbar regions of the spine. Additionally, the regimen included stretching exercises focusing on major muscle groups, such as the erector spinae, shoulders, hip flexors, hamstrings, and quadriceps. The exercise protocol also incorporated breathing exercises, specifically lip contraction breathing, abdominal strengthening during exhalation, and synchronization of chest and abdominal movements. This routine was conducted once daily over a period of six days. | Participants in the combined treatment group were required to engage in freestyle swimming in a community swimming pool maintained at a temperature of 32 °C, in conjunction with their routine exercise regimen, three times per week over a period of six weeks. | NA |
| **Kjeken2013** | The exercise group underwent a comprehensive rehabilitation program that integrated gym-based exercises, aquatic therapy in a heated pool, and outdoor physical activities. This regimen included stretching, strengthening, and cardiorespiratory conditioning to enhance overall health. | The control group received only standard treatment without the rehabilitation exercises during the study period. However, they were provided with the rehabilitation exercise program upon the conclusion of the experiment. |  |
| **Lee2008** | The participants in the Tai Chi group underwent a regimen of Tai Chi, which included a 10-minute warm-up exercise, 21 main movements lasting 30 minutes, and a 5-minute relaxation exercise. | The control group did not receive any alternative treatment throughout the study duration and did not engage in any structured exercise program. | NA |
| **Masiero2014** | The rehabilitation cohort participated in a structured program consisting of 12 supervised sessions, each lasting 60 minutes, conducted twice weekly. These sessions were organized in groups of 6 to 8 participants and were facilitated by a team of experienced physical therapists. The training regimen encompassed exercises focused on stretching, strengthening, aerobics, and enhancing dexterity of the chest, spine, and hips. Following the supervised sessions, participants were instructed to independently perform the learned exercises at home a minimum of three times per week. | The control group did not engage in any exercise training or educational sessions and continued solely with standard treatment involving TNF inhibitors. | NA |
| **Oksüz2023** | The intervention group, which combined aerobic exercise with Pilates, engaged in sessions led by a physical therapist three times per week over an eight-week period. Each session included aerobic training, where participants performed a 5-minute warm-up and a 5-minute relaxation exercise before and after treadmill walking, respectively. Participation in Pilates was mandatory prior to commencing aerobic exercise. | The control group participated solely in aerobic exercise sessions. | NA |
| **Roşu2014** | The intervention group participated in a regimen comprising both Pilates and aerobic exercises.The exercise protocol consisted of 50-minute sessions conducted three times per week over a 48-week period. | The control group engaged exclusively in aerobic exercises. | NA |
| **Salbaş2023** | The experimental group engaged in simulated equestrian exercises utilizing an equestrian simulator | The control group participated in a home-based exercise regimen that encompassed warm-up routines, weight and strength training, balance exercises, and static stretching. | NA |
| **Singh2023** | The yoga intervention group engaged in a comprehensive regimen comprising yoga postures (asanas), breathing techniques (pranayama), meditation (dhyana), and relaxation exercises. Participants attended bi-weekly yoga sessions over a period of three months, with each session lasting 60 minutes. These sessions incorporated relaxation exercises, yoga postures, breathing practices, and meditation. To facilitate home practice, participants were provided with supplementary video and image-based modules that encapsulated the content of each class. | The control group continued to receive standard medical care without the addition of yoga practices. | NA |
| **Soufivand2024** | In the Aqua Stretch group, passive stretching exercises against the wall, facilitated by floating strips, were implemented during the initial two weeks. Subsequently, these exercises transitioned to active stretching against the wall over the following two weeks. The duration of each stretching session progressively increased from 15 seconds to 45 seconds. The stretching protocol was organized to target individual joints initially, followed by a sequence involving interconnected joint movements. | The Aqua Pilates group performed Pilates exercises in the pool for 6 weeks, with a total of 24 training sessions (4 sessions per week). | The control group did not participate in any exercise intervention and continued with standard pharmacological treatment, which included non-steroidal anti-inflammatory drugs (NSAIDs) and tumor necrosis factor inhibitors (Anti-TNF). Additionally, they did not follow any specific drug regimen throughout the study period. |
| **Souza2017** | The Intervention group performed resistance exercises on a Swiss ball in group of a maximum of 4 patients under supervision of a trained physiotherapist. The ball size was chosen according to patient height. For 16  weeks, these patients performed eight exercises twice a week in 50-minute sessions. | The control group remained with medical treatment only, and the identical treatment was offered to them after the end of the study. | NA |
| **Sweeney2002** | The intervention group received educational materials on exercise interventions via mail, aimed at enhancing patients' self-management and encouraging regular physical activity. These materials specifically focused on simple aerobic exercises suitable for individuals with ankylosing spondylitis, to be performed at home. | The control group did not receive these interventions and was only subjected to standard follow-up procedures. | NA |
| **Wang2022** | A 16-week combined exercise program consisting of in-person counseling sessions, supervised training sessions, and aerobic  and functional home-based exercise was given to patients in the intervention group after randomization | The control group did not receive the above-mentioned intervention measures, and only received conventional drug treatment. |  |
| **Xie2019** | Patients in the exercise group (Baduanjin qigong) underwent a 12-week, 2-phase Baduanjin qigong training program. The entire set of Baduanjin qigong exercises in this study consisted of 10 postures. In the first phase, patients were required to attend classes  twice per week for 4 weeks. In the second phase, patients were required to practice Baduanjin qigong at home at least 3 times per week for 8 weeks. | Participants in the no treatment group were required to maintain their current lifestyles for 12 weeks. | NA |

# **Supplementary Table S5 Results of network Meta-Analysis(BASFI).**

| **HS** |  |  |  |  |  |  |  |  |  |  |  |
| --- | --- | --- | --- | --- | --- | --- | --- | --- | --- | --- | --- |
| -0.17 (-1.15,0.81) | **AE+Pilates** |  |  |  |  |  |  |  |  |  |  |
| -0.48 (-1.43,0.46) | -0.32 (-0.89,0.26) | **AE+PT** |  |  |  |  |  |  |  |  |  |
| -0.57 (-1.40,0.27) | -0.40 (-1.07,0.28) | -0.08 (-0.70,0.54) | **Pilates** |  |  |  |  |  |  |  |  |
| -0.59 (-1.50,0.32) | -0.43 (-1.09,0.23) | -0.11 (-0.72,0.49) | -0.03 (-0.59,0.54) | **Yoga** |  |  |  |  |  |  |  |
| -0.63 (-1.42,0.16) | -0.46 (-1.11,0.18) | -0.15 (-0.73,0.43) | -0.07 (-0.54,0.41) | -0.04 (-0.57,0.49) | **AE+SE+Supervise** |  |  |  |  |  |  |
| -0.65 (-1.68,0.39) | -0.48 (-1.30,0.34) | -0.16 (-0.94,0.62) | -0.08 (-0.83,0.67) | -0.05 (-0.79,0.68) | -0.01 (-0.74,0.71) | **RE** |  |  |  |  |  |
| -0.84 (-1.55,-0.13) | -0.67 (-1.35,0.00) | -0.36 (-0.98,0.26) | -0.27 (-0.72,0.17) | -0.25 (-0.82,0.32) | -0.21 (-0.56,0.14) | -0.19 (-0.95,0.56) | **AE+SE** |  |  |  |  |
| -0.88 (-1.76,-0.01) | -0.71 (-1.16,-0.27) | -0.40 (-0.75,-0.04) | -0.32 (-0.82,0.19) | -0.29 (-0.78,0.20) | -0.25 (-0.71,0.21) | -0.24 (-0.93,0.46) | -0.04 (-0.55,0.46) | **AE** |  |  |  |
| -0.89 (-1.67,-0.10) | -0.72 (-1.42,-0.02) | -0.40 (-1.05,0.24) | -0.32 (-0.79,0.15) | -0.29 (-0.89,0.31) | -0.25 (-0.70,0.19) | -0.24 (-1.01,0.53) | -0.05 (-0.38,0.29) | 0.00 (-0.54,0.54) | **SE** |  |  |
| -1.01 (-1.93,-0.08) | -0.84 (-1.43,-0.24) | -0.52 (-1.05,0.01) | -0.44 (-1.03,0.16) | -0.41 (-0.99,0.17) | -0.37 (-0.93,0.19) | -0.36 (-1.12,0.40) | -0.17 (-0.76,0.43) | -0.12 (-0.52,0.27) | -0.12 (-0.74,0.50) | **Qigong** |  |
| -1.08 (-1.91,-0.26) | -0.91 (-1.45,-0.38) | -0.60 (-1.07,-0.13) | -0.51 (-0.93,-0.10) | -0.49 (-0.88,-0.10) | -0.45 (-0.82,-0.08) | -0.43 (-1.06,0.19) | -0.24 (-0.66,0.18) | -0.20 (-0.50,0.11) | -0.20 (-0.65,0.26) | -0.08 (-0.50,0.35) | **CT** |

Results from the network meta-analysis (mixed [network] and indirect comparisons) are presented in the lower left triangle.Deeper background color means statistically significant. The data are the standardized mean difference (95% CI), SMD less than 0 means it can lower the BASFI score. HS:Hippotherapy Simulation; AE:Aerobic Exercises; PT:Physical Therapy; SE:Stretching Exercise; RE:Resistance exercise; CT:Conventional Therapy.

# **Supplementary Table S6 Results of network Meta-Analysis(BASDAI).**

| **AE+Pilates** |  |  |  |  |  |  |  |  |  |  |  |
| --- | --- | --- | --- | --- | --- | --- | --- | --- | --- | --- | --- |
| -0.36 (-1.56,0.84) | **HS** |  |  |  |  |  |  |  |  |  |  |
| -0.73 (-1.59,0.13) | -0.37 (-1.48,0.75) | **Yoga** |  |  |  |  |  |  |  |  |  |
| -0.74 (-1.60,0.11) | -0.38 (-1.37,0.60) | -0.02 (-0.74,0.71) | **Pilates** |  |  |  |  |  |  |  |  |
| -0.83 (-1.66,-0.01) | -0.47 (-1.44,0.50) | -0.11 (-0.80,0.59) | -0.09 (-0.69,0.51) | **AE+SE+Supervise** |  |  |  |  |  |  |  |
| -0.88 (-1.63,-0.14) | -0.52 (-1.61,0.57) | -0.15 (-0.85,0.54) | -0.14 (-0.82,0.55) | -0.05 (-0.70,0.60) | **Qigong** |  |  |  |  |  |  |
| -0.89 (-1.75,-0.02) | -0.53 (-1.36,0.31) | -0.16 (-0.90,0.58) | -0.14 (-0.67,0.39) | -0.05 (-0.55,0.44) | 0.00 (-0.71,0.70) | **AE+SE** |  |  |  |  |  |
| -0.93 (-1.64,-0.21) | -0.57 (-1.67,0.54) | -0.20 (-0.91,0.51) | -0.18 (-0.89,0.52) | -0.09 (-0.77,0.58) | -0.05 (-0.64,0.55) | -0.04 (-0.77,0.68) | **AE+PT** |  |  |  |  |
| -0.92 (-1.80,-0.04) | -0.56 (-1.47,0.34) | -0.19 (-0.95,0.56) | -0.18 (-0.73,0.37) | -0.09 (-0.65,0.47) | -0.04 (-0.76,0.68) | -0.03 (-0.39,0.32) | 0.01 (-0.73,0.75) | **SE** |  |  |  |
| -1.30 (-1.89,-0.71) | -0.94 (-1.99,0.11) | -0.57 (-1.20,0.05) | -0.56 (-1.18,0.06) | -0.47 (-1.05,0.11) | -0.42 (-0.88,0.04) | -0.42 (-1.05,0.22) | -0.37 (-0.78,0.03) | -0.38 (-1.04,0.27) | **AE** |  |  |
| -1.41 (-2.44,-0.38) | -1.05 (-2.30,0.21) | -0.68 (-1.60,0.25) | -0.66 (-1.59,0.26) | -0.57 (-1.47,0.33) | -0.52 (-1.42,0.37) | -0.52 (-1.46,0.41) | -0.48 (-1.39,0.43) | -0.49 (-1.43,0.46) | -0.10 (-0.95,0.74) | **RE** |  |
| -1.51 (-2.20,-0.83) | -1.15 (-2.14,-0.16) | -0.78 (-1.30,-0.27) | -0.77 (-1.28,-0.26) | -0.68 (-1.14,-0.22) | -0.63 (-1.09,-0.17) | -0.63 (-1.16,-0.10) | -0.58 (-1.08,-0.09) | -0.59 (-1.14,-0.04) | -0.21 (-0.56,0.14) | -0.10 (-0.88,0.67) | **CT** |

Results from the network meta-analysis (mixed [network] and indirect comparisons) are presented in the lower left triangle.Deeper background color means statistically significant. The data are the standardized mean difference (95% CI), SMD less than 0 means it can lower the BASFI score. HS:Hippotherapy Simulation; AE:Aerobic Exercises; PT:Physical Therapy; SE:Stretching Exercise; RE:Resistance exercise; CT:Conventional Therapy.

# **Supplementary Table S7 Results of network Meta-Analysis(BASMI)**

| **AE+Pilates** |  |  |  |  |  |  |  |  |  |  |  |
| --- | --- | --- | --- | --- | --- | --- | --- | --- | --- | --- | --- |
| -1.98 (-3.43,-0.52) | **Qigong** |  |  |  |  |  |  |  |  |  |  |
| -2.36 (-3.35,-1.37) | -0.39 (-1.46,0.68) | **AE** |  |  |  |  |  |  |  |  |  |
| -2.44 (-3.88,-1.00) | -0.46 (-1.82,0.89) | -0.08 (-1.12,0.97) | **AE+PT** |  |  |  |  |  |  |  |  |
| -2.93 (-4.53,-1.33) | -0.95 (-2.32,0.41) | -0.57 (-1.83,0.69) | -0.49 (-1.85,0.87) | **AE+SE+Supervise** |  |  |  |  |  |  |  |
| -3.04 (-5.23,-0.85) | -1.07 (-3.09,0.96) | -0.68 (-2.63,1.27) | -0.60 (-2.62,1.42) | -0.11 (-1.70,1.48) | **HS** |  |  |  |  |  |  |
| -3.12 (-4.85,-1.38) | -1.14 (-2.66,0.37) | -0.76 (-2.18,0.67) | -0.68 (-2.19,0.84) | -0.19 (-1.33,0.95) | -0.08 (-1.79,1.64) | **Pilates** |  |  |  |  |  |
| -3.12 (-5.00,-1.23) | -1.14 (-2.83,0.54) | -0.76 (-2.36,0.85) | -0.68 (-2.37,1.00) | -0.19 (-1.74,1.36) | -0.08 (-2.23,2.07) | 0.00 (-1.68,1.68) | **RE** |  |  |  |  |
| -3.16 (-5.05,-1.28) | -1.19 (-2.87,0.50) | -0.80 (-2.40,0.80) | -0.72 (-2.41,0.96) | -0.23 (-1.78,1.31) | -0.12 (-2.27,2.03) | -0.04 (-1.72,1.64) | -0.04 (-1.88,1.79) | **Yoga** |  |  |  |
| -3.26 (-5.00,-1.52) | -1.29 (-2.81,0.23) | -0.90 (-2.33,0.53) | -0.82 (-2.35,0.70) | -0.33 (-1.20,0.53) | -0.22 (-1.55,1.11) | -0.15 (-1.23,0.94) | -0.14 (-1.83,1.54) | -0.10 (-1.79,1.58) | **AE+SE** |  |  |
| -3.28 (-5.14,-1.42) | -1.31 (-2.97,0.35) | -0.92 (-2.50,0.66) | -0.84 (-2.50,0.82) | -0.35 (-1.44,0.74) | -0.24 (-1.73,1.25) | -0.16 (-1.43,1.11) | -0.16 (-1.98,1.65) | -0.12 (-1.93,1.69) | -0.02 (-0.69,0.65) | **SE** |  |
| -3.33 (-4.69,-1.96) | -1.35 (-2.43,-0.27) | -0.96 (-1.91,-0.02) | -0.89 (-1.96,0.19) | -0.40 (-1.23,0.44) | -0.28 (-2.00,1.43) | -0.21 (-1.28,0.86) | -0.21 (-1.50,1.09) | -0.16 (-1.46,1.13) | -0.06 (-1.14,1.01) | -0.04 (-1.31,1.22) | **CT** |

Results from the network meta-analysis (mixed [network] and indirect comparisons) are presented in the lower left triangle.Deeper background color means statistically significant. The data are the standardized mean difference (95% CI), SMD less than 0 means it can lower the BASFI score. HS:Hippotherapy Simulation; AE:Aerobic Exercises; PT:Physical Therapy; SE:Stretching Exercise; RE:Resistance exercise; CT:Conventional Therapy.

# **Supplementary Table S8 Results of network Meta-Analysis(CE)**

| **AE+SE+Supervise** |  |  |  |  |  |  |  |  |  |
| --- | --- | --- | --- | --- | --- | --- | --- | --- | --- |
| 0.07 (-0.96,1.10) | **AE+Pilates** |  |  |  |  |  |  |  |  |
| 0.40 (0.07,0.72) | 0.32 (-0.69,1.34) | **AE+SE** |  |  |  |  |  |  |  |
| 0.50 (0.09,0.90) | 0.42 (-0.60,1.45) | 0.10 (-0.17,0.37) | **SE** |  |  |  |  |  |  |
| 0.55 (-0.01,1.10) | 0.47 (-0.54,1.49) | 0.15 (-0.36,0.66) | 0.05 (-0.45,0.56) | **Pilates** |  |  |  |  |  |
| 0.74 (0.05,1.42) | 0.66 (-0.41,1.74) | 0.34 (-0.33,1.01) | 0.24 (-0.43,0.92) | 0.19 (-0.47,0.85) | **RE** |  |  |  |  |
| 0.89 (0.16,1.61) | 0.82 (0.08,1.55) | 0.49 (-0.21,1.20) | 0.39 (-0.32,1.11) | 0.34 (-0.36,1.05) | 0.15 (-0.63,0.94) | **Qigong** |  |  |  |
| 0.86 (0.42,1.29) | 0.78 (-0.15,1.72) | 0.46 (0.05,0.87) | 0.36 (-0.06,0.78) | 0.31 (-0.09,0.71) | 0.12 (-0.41,0.65) | -0.03 (-0.61,0.54) | **CT** |  |  |
| 1.74 (0.68,2.79) | 1.67 (0.94,2.39) | 1.34 (0.30,2.39) | 1.24 (0.19,2.29) | 1.19 (0.15,2.23) | 1.00 (-0.09,2.10) | 0.85 (0.08,1.62) | 0.88 (-0.08,1.84) | **AE+PT** |  |
| 1.97 (1.06,2.88) | 1.90 (1.41,2.39) | 1.58 (0.68,2.47) | 1.48 (0.57,2.38) | 1.42 (0.53,2.32) | 1.23 (0.28,2.19) | 1.08 (0.53,1.63) | 1.12 (0.32,1.91) | 0.23 (-0.30,0.77) | **AE** |

Results from the network meta-analysis (mixed [network] and indirect comparisons) are presented in the lower left triangle.Deeper background color means statistically significant. The data are the standardized mean difference (95% CI), SMD less than 0 means it can lower the BASFI score. HS:Hippotherapy Simulation; AE:Aerobic Exercises; PT:Physical Therapy; SE:Stretching Exercise; RE:Resistance exercise; CT:Conventional Therapy.

**Supplementary Table S9 Meta Regression.**

| Outcomes | Year | Treatment | Country | Sample Size | Dose |
| --- | --- | --- | --- | --- | --- |
| BASFI | **0.01** | 0.26 | 0.56 | **0.006** | 0.418 |
| BASDAI | 0.479 | 0.136 | 0.65 | 0.186 | 0.516 |
| BASMI | 0.512 | 0.476 | 0.272 | 0.364 | 0.48 |
| CE | 0.764 | 0.228 | 0.655 | 0.162 | 0.169 |

BASFI:Bath Ankylosing Spondylitis Functional Index; BASDAI:Bath Ankylosing Spondylitis Disease Activity Index; BASMI:Bath Ankylosing Spondylitis Metrology Index; CE:Chest Expansion .Bold font represents statistical significance.

# **Supplementary Table S10 Results of network Meta-Analysis(BASFI, Removal of High Risk Studies)**

| **HS** |  |  |  |  |  |  |  |  |  |  |  |
| --- | --- | --- | --- | --- | --- | --- | --- | --- | --- | --- | --- |
| -0.23 (-1.29,0.83) | **AE+Pilates** |  |  |  |  |  |  |  |  |  |  |
| -0.28 (-1.37,0.82) | -0.04 (-0.87,0.78) | **Yoga** |  |  |  |  |  |  |  |  |  |
| -0.47 (-1.25,0.31) | -0.24 (-1.02,0.55) | -0.19 (-1.02,0.64) | **AE+SE+Supervise** |  |  |  |  |  |  |  |  |
| -0.52 (-1.40,0.37) | -0.28 (-1.04,0.47) | -0.24 (-1.04,0.56) | -0.05 (-0.64,0.54) | **Pilates** |  |  |  |  |  |  |  |
| -0.55 (-1.59,0.48) | -0.32 (-0.87,0.23) | -0.28 (-1.07,0.51) | -0.09 (-0.83,0.66) | -0.04 (-0.76,0.68) | **AE+PT** |  |  |  |  |  |  |
| -0.84 (-1.53,-0.15) | -0.61 (-1.41,0.20) | -0.56 (-1.41,0.29) | -0.37 (-0.74,0.00) | -0.32 (-0.88,0.23) | -0.29 (-1.06,0.49) | **AE+SE** |  |  |  |  |  |
| -0.95 (-1.93,0.02) | -0.72 (-1.15,-0.29) | -0.68 (-1.38,0.03) | -0.48 (-1.15,0.18) | -0.44 (-1.07,0.20) | -0.40 (-0.74,-0.06) | -0.11 (-0.80,0.58) | **AE** |  |  |  |  |
| -1.04 (-1.82,-0.26) | -0.80 (-1.69,0.09) | -0.76 (-1.68,0.17) | -0.57 (-1.09,-0.05) | -0.52 (-1.18,0.14) | -0.48 (-1.34,0.38) | -0.20 (-0.56,0.17) | -0.08 (-0.87,0.71) | **SE** |  |  |  |
| -1.03 (-1.94,-0.12) | -0.80 (-1.35,-0.25) | -0.75 (-1.36,-0.14) | -0.56 (-1.12,-0.01) | -0.51 (-1.03,0.01) | -0.48 (-0.98,0.02) | -0.19 (-0.78,0.40) | -0.08 (-0.44,0.28) | 0.00 (-0.69,0.70) | **CT** |  |  |
| -1.15 (-2.22,-0.07) | -0.91 (-1.53,-0.29) | -0.87 (-1.70,-0.03) | -0.68 (-1.47,0.12) | -0.63 (-1.40,0.14) | -0.59 (-1.16,-0.02) | -0.30 (-1.12,0.51) | -0.19 (-0.64,0.26) | -0.11 (-1.01,0.79) | -0.11 (-0.68,0.46) | **Qigong** |  |

Results from the network meta-analysis (mixed [network] and indirect comparisons) are presented in the lower left triangle.Deeper background color means statistically significant. The data are the standardized mean difference (95% CI), SMD less than 0 means it can lower the BASFI score. HS:Hippotherapy Simulation; AE:Aerobic Exercises; PT:Physical Therapy; SE:Stretching Exercise; CT:Conventional Therapy.

# **Supplementary Table S11 Results of network Meta-Analysis(BASDAI, Removal of High Risk Studies)**

| **AE+Pilates** |  |  |  |  |  |  |  |  |  |  |  |
| --- | --- | --- | --- | --- | --- | --- | --- | --- | --- | --- | --- |
| -0.80 (-1.66,0.06) | **Qigong** |  |  |  |  |  |  |  |  |  |  |
| -0.85 (-2.03,0.33) | -0.05 (-1.13,1.02) | **Yoga** |  |  |  |  |  |  |  |  |  |
| -0.90 (-2.00,0.19) | -0.11 (-1.08,0.87) | -0.06 (-1.20,1.09) | **Pilates** |  |  |  |  |  |  |  |  |
| -0.93 (-1.73,-0.14) | -0.14 (-0.84,0.57) | -0.09 (-1.13,0.96) | -0.03 (-0.97,0.91) | **AE+PT** |  |  |  |  |  |  |  |
| -0.91 (-2.38,0.56) | -0.11 (-1.51,1.28) | -0.06 (-1.57,1.45) | -0.01 (-1.20,1.19) | 0.02 (-1.34,1.39) | **HS** |  |  |  |  |  |  |
| -1.27 (-2.37,-0.16) | -0.47 (-1.46,0.52) | -0.42 (-1.57,0.74) | -0.36 (-1.20,0.47) | -0.33 (-1.29,0.62) | -0.36 (-1.45,0.74) | **AE+SE+Supervise** |  |  |  |  |  |
| -1.31 (-1.96,-0.66) | -0.51 (-1.08,0.05) | -0.46 (-1.44,0.52) | -0.41 (-1.28,0.47) | -0.38 (-0.83,0.08) | -0.40 (-1.72,0.92) | -0.04 (-0.94,0.85) | **AE** |  |  |  |  |
| -1.44 (-2.59,-0.28) | -0.64 (-1.69,0.41) | -0.59 (-1.79,0.62) | -0.53 (-1.30,0.23) | -0.50 (-1.52,0.51) | -0.53 (-1.44,0.39) | -0.17 (-0.77,0.43) | -0.13 (-1.08,0.83) | **AE+SE** |  |  |  |
| -1.50 (-2.30,-0.70) | -0.70 (-1.34,-0.07) | -0.65 (-1.52,0.22) | -0.60 (-1.34,0.15) | -0.57 (-1.14,0.01) | -0.59 (-1.83,0.65) | -0.23 (-0.99,0.53) | -0.19 (-0.65,0.28) | -0.06 (-0.90,0.77) | **CT** |  |  |
| -1.61 (-2.84,-0.38) | -0.81 (-1.95,0.32) | -0.76 (-2.04,0.51) | -0.71 (-1.58,0.17) | -0.68 (-1.78,0.42) | -0.70 (-1.71,0.31) | -0.34 (-1.08,0.39) | -0.30 (-1.34,0.74) | -0.17 (-0.60,0.25) | -0.11 (-1.05,0.82) | **SE** |  |

Results from the network meta-analysis (mixed [network] and indirect comparisons) are presented in the lower left triangle.Deeper background color means statistically significant. The data are the standardized mean difference (95% CI), SMD less than 0 means it can lower the BASFI score. HS:Hippotherapy Simulation; AE:Aerobic Exercises; PT:Physical Therapy; SE:Stretching Exercise; CT:Conventional Therapy.

# **Supplementary Table S12 Results of network Meta-Analysis(BASMI, Removal of High Risk Studies)**

| **AE+Pilates** |  |  |  |  |  |  |  |  |  |  |  |
| --- | --- | --- | --- | --- | --- | --- | --- | --- | --- | --- | --- |
| -2.02 (-3.92,-0.11) | **Qigong** |  |  |  |  |  |  |  |  |  |  |
| -2.33 (-3.56,-1.11) | -0.32 (-1.78,1.14) | **AE** |  |  |  |  |  |  |  |  |  |
| -2.49 (-4.37,-0.60) | -0.47 (-2.16,1.22) | -0.15 (-1.59,1.28) | **AE+PT** |  |  |  |  |  |  |  |  |
| -2.90 (-5.42,-0.38) | -0.89 (-2.93,1.16) | -0.57 (-2.77,1.63) | -0.42 (-2.46,1.63) | **AE+SE+Supervise** |  |  |  |  |  |  |  |
| -3.04 (-6.13,0.04) | -1.03 (-3.74,1.68) | -0.71 (-3.54,2.13) | -0.56 (-3.27,2.16) | -0.14 (-2.14,1.86) | **HS** |  |  |  |  |  |  |
| -3.18 (-5.69,-0.67) | -1.16 (-3.19,0.87) | -0.84 (-3.03,1.35) | -0.69 (-2.73,1.35) | -0.27 (-1.82,1.27) | -0.13 (-2.32,2.06) | **Pilates** |  |  |  |  |  |
| -3.28 (-5.93,-0.62) | -1.26 (-3.47,0.95) | -0.94 (-3.30,1.42) | -0.79 (-3.00,1.43) | -0.37 (-2.54,1.80) | -0.23 (-3.04,2.58) | -0.10 (-2.26,2.06) | **Yoga** |  |  |  |  |
| -3.27 (-5.86,-0.67) | -1.25 (-3.38,0.88) | -0.93 (-3.22,1.36) | -0.78 (-2.92,1.36) | -0.36 (-1.46,0.74) | -0.22 (-1.89,1.45) | -0.09 (-1.50,1.32) | 0.01 (-2.25,2.27) | **AE+SE** |  |  |  |
| -3.29 (-6.06,-0.52) | -1.28 (-3.62,1.07) | -0.96 (-3.45,1.53) | -0.81 (-3.16,1.55) | -0.39 (-1.86,1.08) | -0.25 (-2.19,1.69) | -0.12 (-1.83,1.60) | -0.02 (-2.48,2.44) | -0.03 (-1.00,0.95) | **SE** |  |  |
| -3.44 (-5.52,-1.36) | -1.42 (-2.90,0.05) | -1.10 (-2.79,0.58) | -0.95 (-2.43,0.53) | -0.54 (-1.95,0.88) | -0.40 (-2.67,1.88) | -0.26 (-1.66,1.13) | -0.16 (-1.81,1.48) | -0.17 (-1.72,1.37) | -0.15 (-1.97,1.68) | **CT** |  |

Results from the network meta-analysis (mixed [network] and indirect comparisons) are presented in the lower left triangle.Deeper background color means statistically significant. The data are the standardized mean difference (95% CI), SMD less than 0 means it can lower the BASFI score. HS:Hippotherapy Simulation; AE:Aerobic Exercises; PT:Physical Therapy; SE:Stretching Exercise; CT:Conventional Therapy.

**Supplementary Table S13 The SUCRA Values of Each Treatment Modality (Removal of High Risk Studies).**

| Treatment | BASFI | Rank | BASDAI | Rank | BASMI | Rank |
| --- | --- | --- | --- | --- | --- | --- |
| CT | 18.5% | 10 | 18.6% | 10 | 24.4% | 11 |
| AE | 26.1% | 8 | 32.7% | 8 | 63% | 3 |
| Yoga | 76.9% | 3 | 63.3% | 4 | 33.9% | 8 |
| Qigong | 13.5% | 11 | 69.2% | 2 | 74.3% | 2 |
| AE+SE | 35.6% | 7 | 27.1% | 9 | 32.8% | 10 |
| AE+SE+Supervise | 67.1% | 4 | 39.3% | 7 | 49.2% | 5 |
| HS | 89.1% | 1 | 61.3% | 6 | 43.3% | 6 |
| SE | 20.1% | 9 | 15.8% | 11 | 33.4% | 9 |
| AE+Pilates | 81.2% | 2 | 96.9% | 1 | 99% | 1 |
| AE+PT | 59.2% | 6 | 61.7% | 5 | 59.6% | 4 |
| Pilates | 62.6% | 5 | 64% | 3 | 37.2% | 7 |

CT:Conventional Therapy; AE:Aerobic Exercises; RE: Resistance Exercise; PT:Physical Therapy; SE:Stretching Exercise; HS:Hippotherapy Simulation.BASFI:Bath Ankylosing Spondylitis Functional Index; BASDAI:Bath Ankylosing Spondylitis Disease Activity Index; BASMI:Bath Ankylosing Spondylitis Metrology Index.

**Supplementary Table S14 The Grading of Recommendations Assessment, Development and Evaluation (GRADE) assessment for BASFI.**

| Comparison | N of studies | Within-study bias | Reporting bias | Indirectness | Imprecision | Heterogeneity | Incoherence | Confidence rating |
| --- | --- | --- | --- | --- | --- | --- | --- | --- |
| AE vs CT | 3 | Major concerns | Low risk | No concerns | Some concerns | No concerns | No concerns | Very Low |
| AE+SE+Supervise vs CT | 2 | Some concerns | Low risk | No concerns | No concerns | Some concerns | No concerns | Low |
| Pilates vs CT | 2 | Major concerns | Low risk | No concerns | No concerns | No concerns | No concerns | Low |
| Qigong vs CT | 1 | Some concerns | Low risk | No concerns | Some concerns | No concerns | No concerns | Low |
| RE vs CT | 1 | Major concerns | Low risk | No concerns | Some concerns | No concerns | No concerns | Very Low |
| SE vs CT | 1 | Major concerns | Low risk | No concerns | Some concerns | No concerns | No concerns | Very Low |
| Yoga vs CT | 2 | Some concerns | Low risk | No concerns | No concerns | No concerns | No concerns | Moderate |
| AE vs Qigong | 2 | Some concerns | Low risk | No concerns | Some concerns | No concerns | No concerns | Low |
| AE vs AE+PT | 3 | Some concerns | Low risk | No concerns | No concerns | No concerns | No concerns | Moderate |
| AE+SE vs AE+SE+Supervise | 3 | Some concerns | Low risk | No concerns | Some concerns | No concerns | No concerns | Low |
| AE+SE vs HS | 1 | Some concerns | Low risk | No concerns | No concerns | No concerns | No concerns | Moderate |
| AE+SE vs SE | 4 | Some concerns | Low risk | No concerns | Some concerns | No concerns | No concerns | Low |
| AE+SE vs Pilates | 1 | Some concerns | Low risk | No concerns | Some concerns | No concerns | No concerns | Low |
| AE vs AE+Pilates | 2 | Some concerns | Low risk | No concerns | No concerns | No concerns | No concerns | Moderate |
| SE vs Pliates | 1 | Major concerns | Low risk | No concerns | Some concerns | No concerns | No concerns | Very Low |

The interventions in this study are similar in terms of Reporting bias, Indirectness, and Incoherence; therefore, we provide the following criteria for evaluating these three factors:

**Within-study bias:** high risk: If the study has unclear randomization processes, improper blinding methods, or significant missing data that were not adequately addressed. Low risk: If the study reports a reasonable randomization method, employs appropriate blinding, and has minimal missing data that were handled appropriately.

**Imprecision:** If the width of the 95% confidence interval is greater than 0.2, or if the confidence interval includes zero, the result is considered to have high imprecision.

**Heterogeneity:** low heterogeneity: I² < 40%, moderate heterogeneity: I² between 40% and 60%, high heterogeneity: I² > 60%.

CT:Conventional Therapy; AE:Aerobic Exercises; RE: Resistance Exercise; PT:Physical Therapy; SE:Stretching Exercise; HS:Hippotherapy Simulation.

**Supplementary Table S15 The Grading of Recommendations Assessment, Development and Evaluation (GRADE) assessment for BASDAI.**

| Comparison | N of studies | Within-study bias | Reporting bias | Indirectness | Imprecision | Heterogeneity | Incoherence | Confidence rating |
| --- | --- | --- | --- | --- | --- | --- | --- | --- |
| AE vs CT | 3 | Major concerns | Low risk | No concerns | Some concerns | No concerns | No concerns | Very Low |
| Qigong vs CT | 2 | Major concerns | Low risk | No concerns | No concerns | No concerns | No concerns | Low |
| AE+SE+Supervise vs CT | 2 | Major concerns | Low risk | No concerns | No concerns | No concerns | No concerns | Low |
| SE vs CT | 1 | Major concerns | Low risk | No concerns | No concerns | No concerns | No concerns | Low |
| AE+PT vs CT | 1 | Some concerns | Low risk | No concerns | No concerns | No concerns | No concerns | Moderate |
| RE vs CT | 1 | Major concerns | Low risk | No concerns | Some concerns | No concerns | No concerns | Very Low |
| Yoga vs CT | 2 | Major concerns | Low risk | No concerns | No concerns | No concerns | No concerns | Low |
| Pilates vs CT | 2 | Major concerns | Low risk | No concerns | No concerns | No concerns | No concerns | Low |
| AE+SE vs SE | 5 | Major concerns | Low risk | No concerns | Some concerns | No concerns | No concerns | Very Low |
| AE+SE vs HS | 1 | Some concerns | Low risk | No concerns | Some concerns | No concerns | No concerns | Low |
| AE vs AE+PT | 3 | Some concerns | Low risk | No concerns | Some concerns | No concerns | No concerns | Low |
| AE vs Qigong | 2 | Some concerns | Low risk | No concerns | Some concerns | No concerns | No concerns | Low |
| SE vs Pilates | 1 | Major concerns | Low risk | No concerns | Some concerns | No concerns | No concerns | Very Low |
| AE+SE vs Pilates | 1 | Some concerns | Low risk | No concerns | Some concerns | No concerns | No concerns | Low |
| AE+SE vs AE+SE+Supervise | 2 | Some concerns | Low risk | No concerns | Some concerns | No concerns | No concerns | Low |
| AE vs AE+Pilates | 2 | Some concerns | Low risk | No concerns | No concerns | No concerns | No concerns | Moderate |

The interventions in this study are similar in terms of Reporting bias, Indirectness, and Incoherence; therefore, we provide the following criteria for evaluating these three factors:

**Within-study bias:** high risk: If the study has unclear randomization processes, improper blinding methods, or significant missing data that were not adequately addressed. Low risk: If the study reports a reasonable randomization method, employs appropriate blinding, and has minimal missing data that were handled appropriately.

**Imprecision:** If the width of the 95% confidence interval is greater than 0.2, or if the confidence interval includes zero, the result is considered to have high imprecision.

**Heterogeneity:** low heterogeneity: I² < 40%, moderate heterogeneity: I² between 40% and 60%, high heterogeneity: I² > 60%.

CT:Conventional Therapy; AE:Aerobic Exercises; RE: Resistance Exercise; PT:Physical Therapy; SE:Stretching Exercise; HS:Hippotherapy Simulation.

**Supplementary Table S16 The Grading of Recommendations Assessment, Development and Evaluation (GRADE) assessment for BASMI.**

| Comparison | N of studies | Within-study bias | Reporting bias | Indirectness | Imprecision | Heterogeneity | Incoherence | Confidence rating |
| --- | --- | --- | --- | --- | --- | --- | --- | --- |
| AE vs CT | 1 | Major concerns | Low risk | No concerns | No concerns | No concerns | No concerns | Low |
| Yoga vs CT | 1 | Some concerns | Low risk | No concerns | Some concerns | No concerns | No concerns | Low |
| Qigong vs CT | 1 | Major concerns | Low risk | No concerns | No concerns | No concerns | No concerns | Moderate |
| RE vs CT | 1 | Major concerns | Low risk | No concerns | Some concerns | No concerns | No concerns | Very Low |
| AE+SE+Supervise vs CT | 2 | Major concerns | Low risk | No concerns | Some concerns | No concerns | No concerns | Very Low |
| AE+PT vs CT | 1 | Some concerns | Low risk | No concerns | Some concerns | No concerns | No concerns | Low |
| Pliates vs CT | 1 | Some concerns | Low risk | No concerns | Some concerns | No concerns | No concerns | Low |
| AE+SE vs AE+SE+Supervise | 2 | Some concerns | Low risk | No concerns | Some concerns | No concerns | No concerns | Low |
| AE+SE vs HS | 1 | Some concerns | Low risk | No concerns | Some concerns | No concerns | No concerns | Low |
| AE+SE vs SE | 4 | Some concerns | Low risk | No concerns | Some concerns | No concerns | No concerns | Low |
| AE vs AE+Pilates | 2 | Some concerns | Low risk | No concerns | No concerns | No concerns | No concerns | Moderate |
| AE vs AE+PT | 1 | Some concerns | Low risk | No concerns | Some concerns | No concerns | No concerns | Low |
| AE vs Qigong | 1 | Some concerns | Low risk | No concerns | Some concerns | No concerns | No concerns | Low |
| AE+SE vs Pilates | 1 | Some concerns | Low risk | No concerns | Some concerns | No concerns | No concerns | Low |

The interventions in this study are similar in terms of Reporting bias, Indirectness, and Incoherence; therefore, we provide the following criteria for evaluating these three factors:

**Within-study bias:** high risk: If the study has unclear randomization processes, improper blinding methods, or significant missing data that were not adequately addressed. Low risk: If the study reports a reasonable randomization method, employs appropriate blinding, and has minimal missing data that were handled appropriately.

**Imprecision:** If the width of the 95% confidence interval is greater than 0.2, or if the confidence interval includes zero, the result is considered to have high imprecision.

**Heterogeneity:** low heterogeneity: I² < 40%, moderate heterogeneity: I² between 40% and 60%, high heterogeneity: I² > 60%.

CT:Conventional Therapy; AE:Aerobic Exercises; RE: Resistance Exercise; PT:Physical Therapy; SE:Stretching Exercise; HS:Hippotherapy Simulation.

**Supplementary Table S17 The Grading of Recommendations Assessment, Development and Evaluation (GRADE) assessment for CE.**

| Comparison | N of studies | Within-study bias | Reporting bias | Indirectness | Imprecision | Heterogeneity | Incoherence | Confidence rating |
| --- | --- | --- | --- | --- | --- | --- | --- | --- |
| AE vs CT | 1 | Some concerns | Low risk | No concerns | No concerns | No concerns | No concerns | Moderate |
| AE+SE+Supervise vs CT | 1 | Some concerns | Low risk | No concerns | No concerns | No concerns | No concerns | Moderate |
| Pilates vs CT | 2 | Major concerns | Low risk | No concerns | Some concerns | No concerns | No concerns | Very Low |
| Qigong vs CT | 1 | Major concerns | Low risk | No concerns | Some concerns | No concerns | No concerns | Very Low |
| RE vs CT | 1 | Major concerns | Low risk | No concerns | Some concerns | No concerns | No concerns | Very Low |
| SE vs CT | 1 | Major concerns | Low risk | No concerns | Some concerns | No concerns | No concerns | Very Low |
| AE+SE vs SE | 4 | Major concerns | Low risk | No concerns | Some concerns | No concerns | No concerns | Very Low |
| SE vs Pliates | 1 | Major concerns | Low risk | No concerns | Some concerns | No concerns | No concerns | Very Low |
| AE vs AE+Pilates | 1 | Some concerns | Low risk | No concerns | No concerns | No concerns | No concerns | Moderate |
| AE vs AE+PT | 1 | Some concerns | Low risk | No concerns | Some concerns | No concerns | No concerns | Low |
| AE vs Qigong | 1 | Some concerns | Low risk | No concerns | No concerns | No concerns | No concerns | Moderate |
| AE+SE vs AE+SE+Supervise | 3 | Some concerns | Low risk | No concerns | No concerns | No concerns | No concerns | Moderate |

The interventions in this study are similar in terms of Reporting bias, Indirectness, and Incoherence; therefore, we provide the following criteria for evaluating these three factors:

**Within-study bias:** high risk: If the study has unclear randomization processes, improper blinding methods, or significant missing data that were not adequately addressed. Low risk: If the study reports a reasonable randomization method, employs appropriate blinding, and has minimal missing data that were handled appropriately.

**Imprecision:** If the width of the 95% confidence interval is greater than 0.2, or if the confidence interval includes zero, the result is considered to have high imprecision.

**Heterogeneity:** low heterogeneity: I² < 40%, moderate heterogeneity: I² between 40% and 60%, high heterogeneity: I² > 60%

CT:Conventional Therapy; AE:Aerobic Exercises; RE: Resistance Exercise; PT:Physical Therapy; SE:Stretching Exercise; HS:Hippotherapy Simulation;CE:Chest Expansion .

**Figure S1 Risk of bias assessment included in the study.**


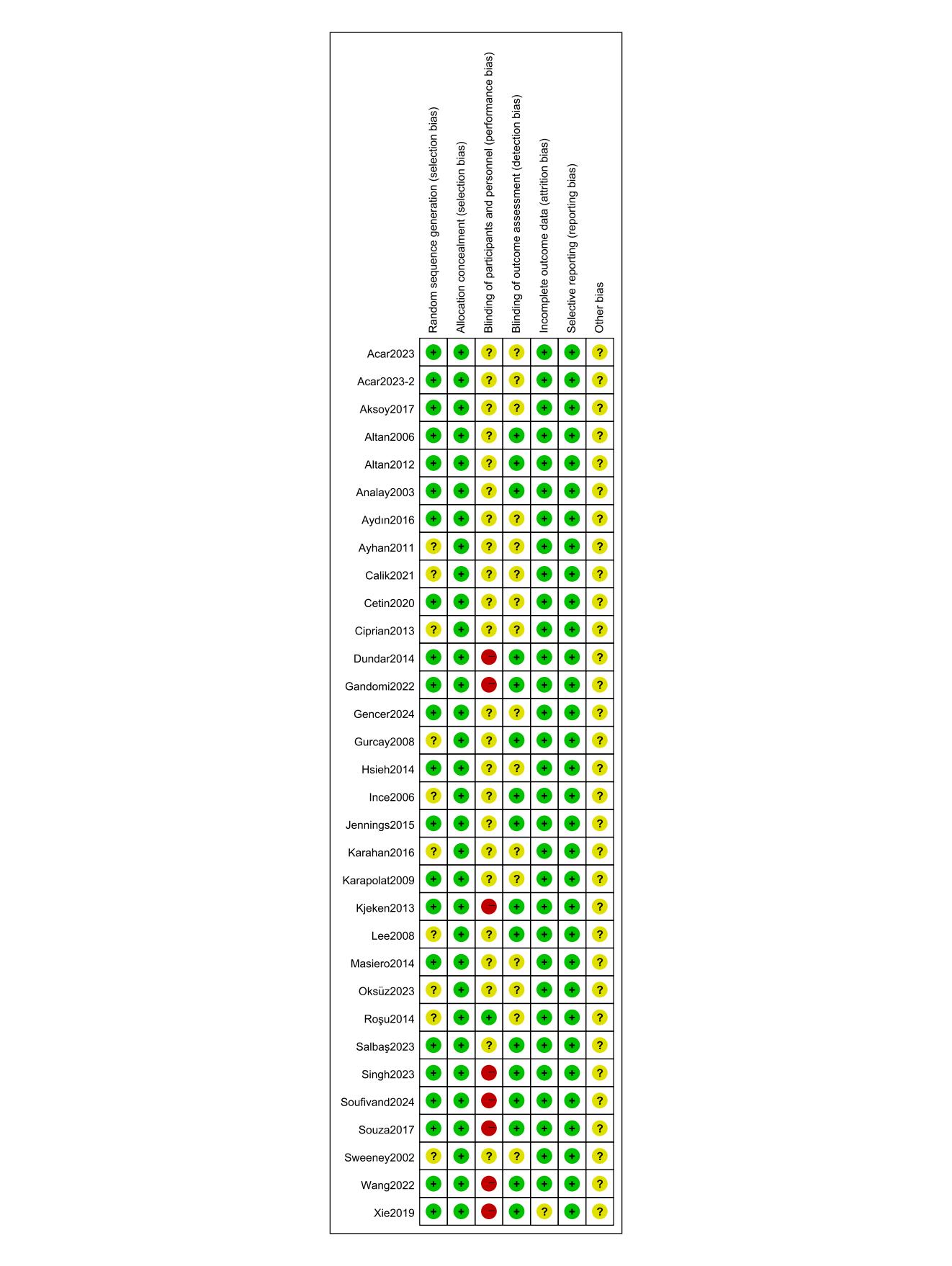


**Figure S2** **A pairwise comparison of the BASFI scores was conducted between all exercise interventions and CT.**
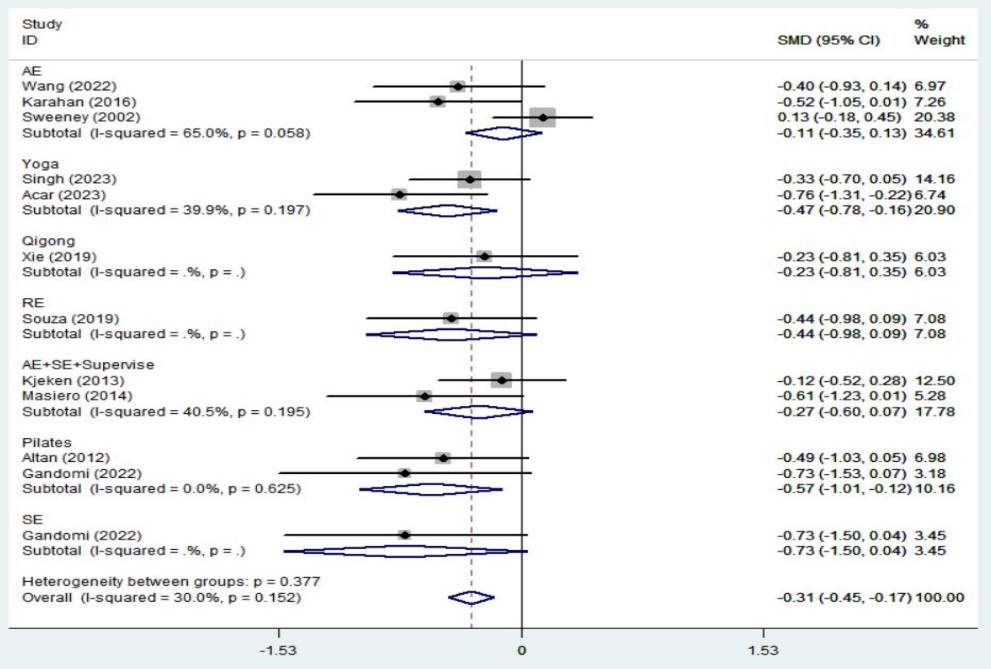


CT:Conventional Therapy; AE:Aerobic Exercises; RE: Resistance Exercise; SE:Stretching Exercise.

**Figure S3** **Non-concordance testing for BASFI scores.**


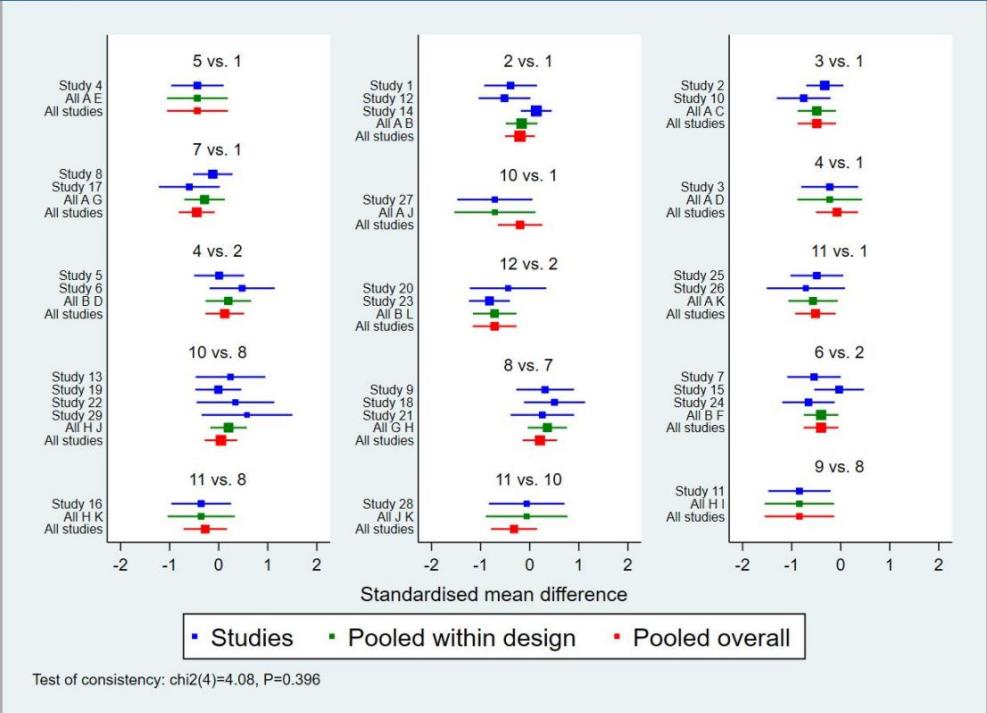


Note:1.CT;2.AE;3.Yoga;4.Qigong;5.RE;6.AE+PT;7.AE+SE+Supervise;8.AE+SE;9.HS;10.SE;11.Pilates;12.AE+Pilates

CT:Conventional Therapy; AE:Aerobic Exercises; RE: Resistance Exercise; PT:Physical Therapy; SE:Stretching Exercise; HS:Hippotherapy Simulation

**Figure S4** **A pairwise comparison was conducted among the BASFI scores, AE + SE + Supervise, and CT, as well as between SE and CT.**


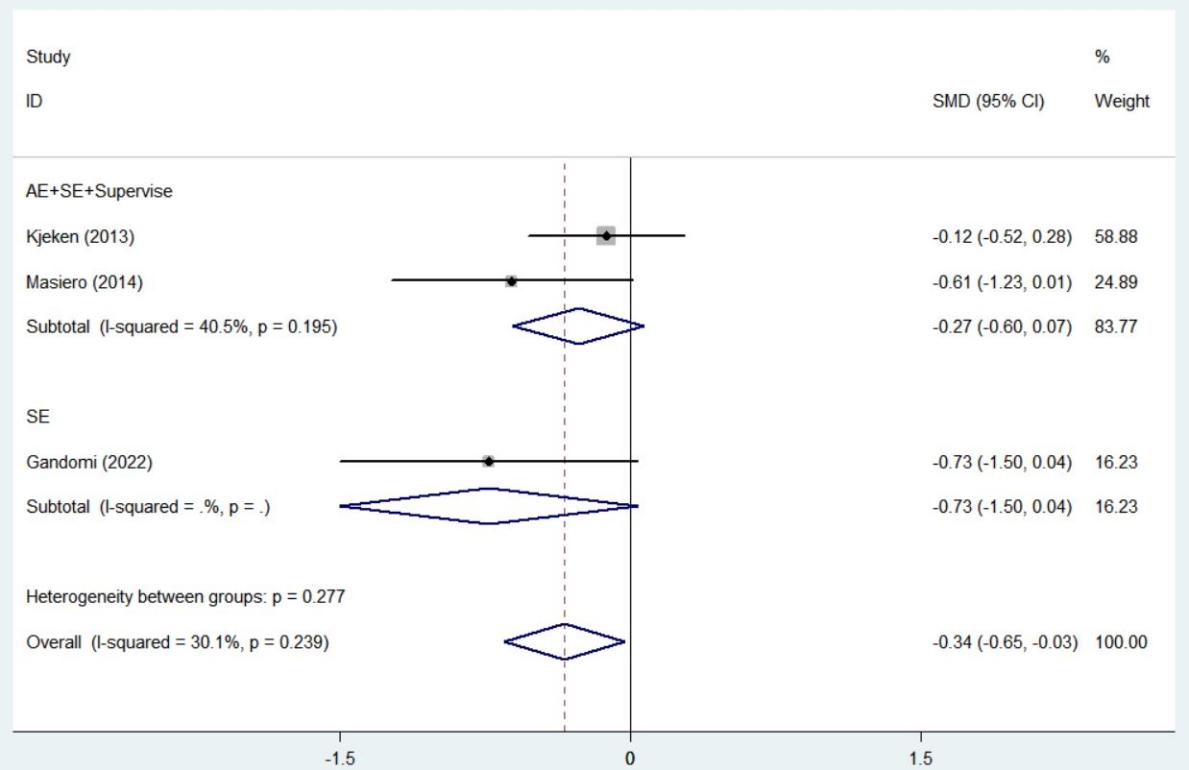


CT:Conventional Therapy; AE:Aerobic Exercises; SE:Stretching Exercise.

**Figure S5 BASFI score, pairwise comparison between AE + SE + Supervise and AE + SE.**


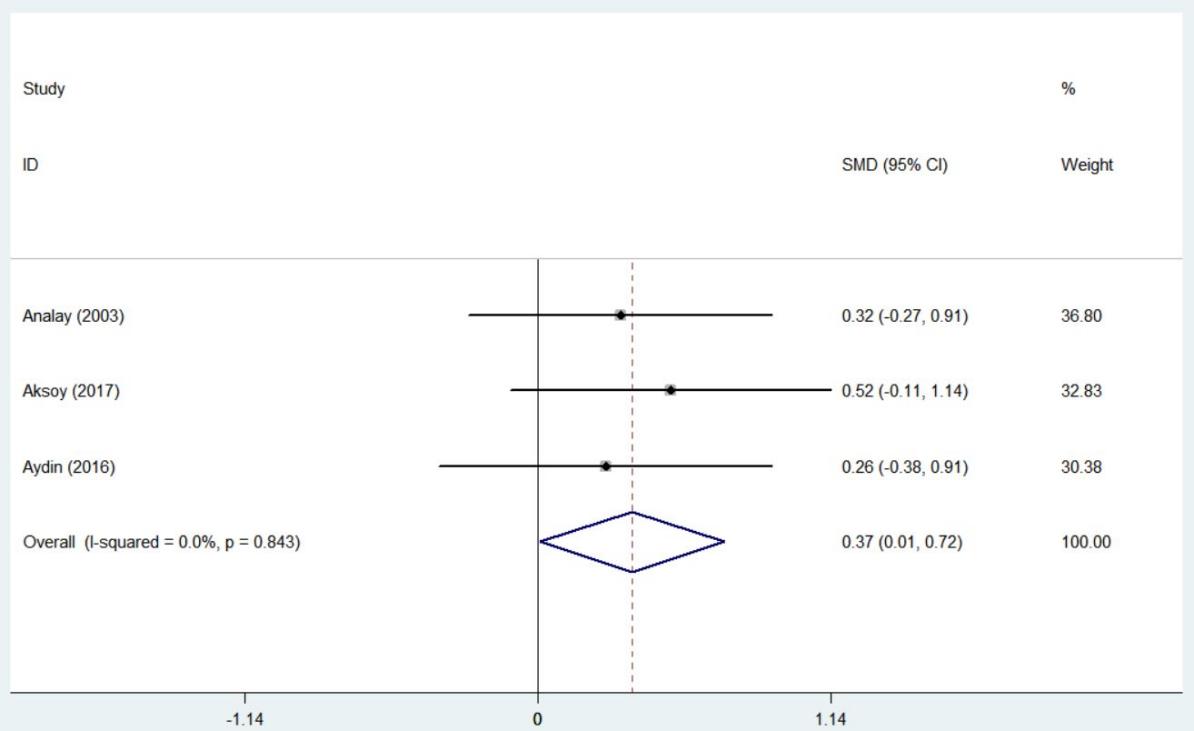


CT:Conventional Therapy; AE:Aerobic Exercises; SE:Stretching Exercise.

**Figure S6** **BASFI score, pairwise comparison between AE + SE and SE.**


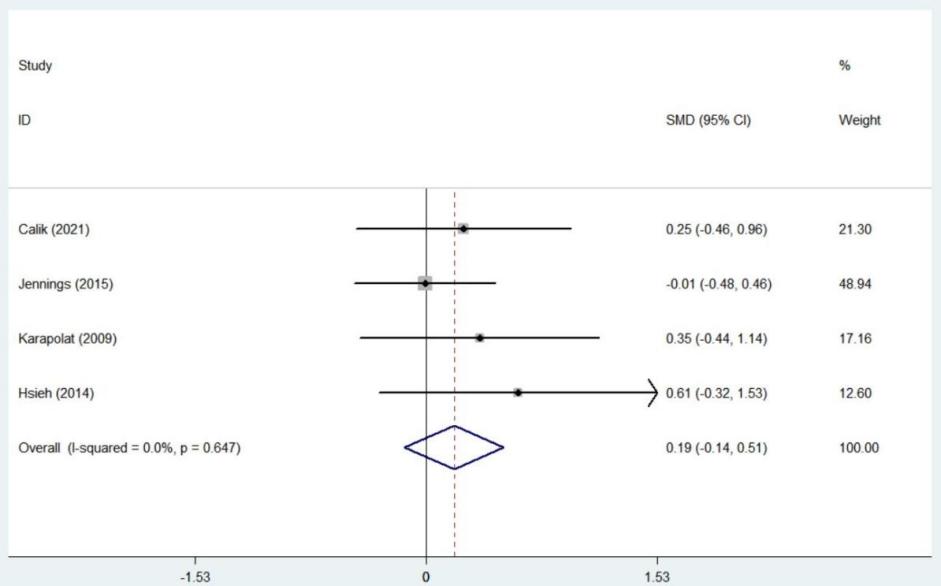


CT:Conventional Therapy; AE:Aerobic Exercises; SE:Stretching Exercise.

**Figure S7** **A pairwise comparison of the BASDAI scores was conducted between all exercise interventions and CT.**


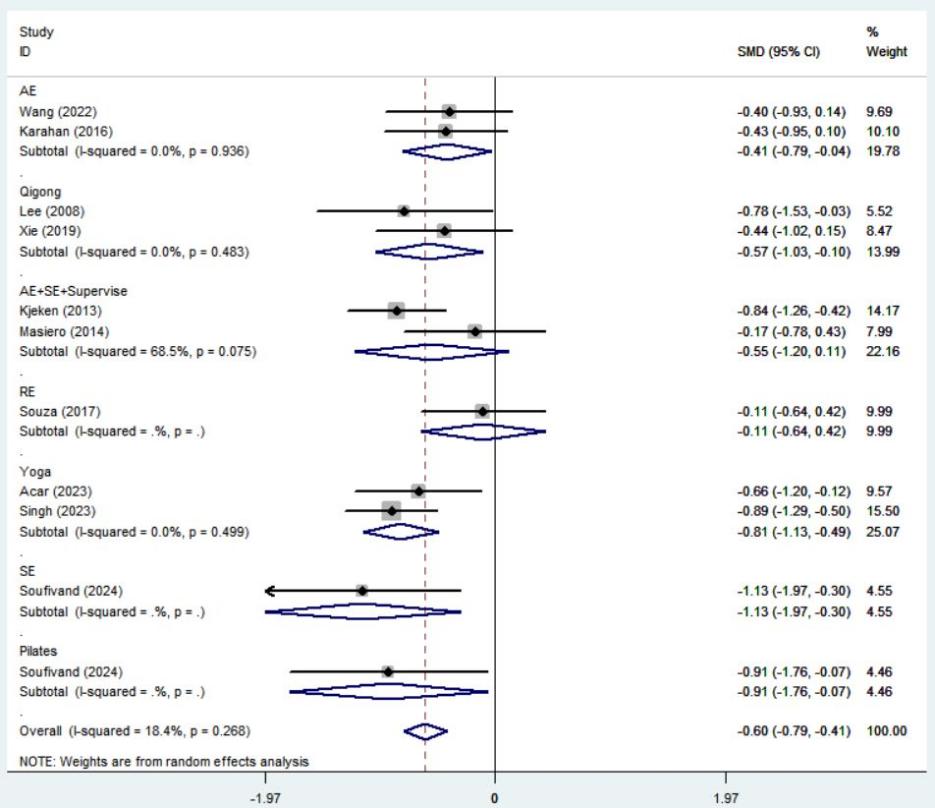


CT:Conventional Therapy; AE:Aerobic Exercises; RE: Resistance Exercise; SE:Stretching Exercise

**Figure S8 Non-concordance testing for BASDAI scores.**
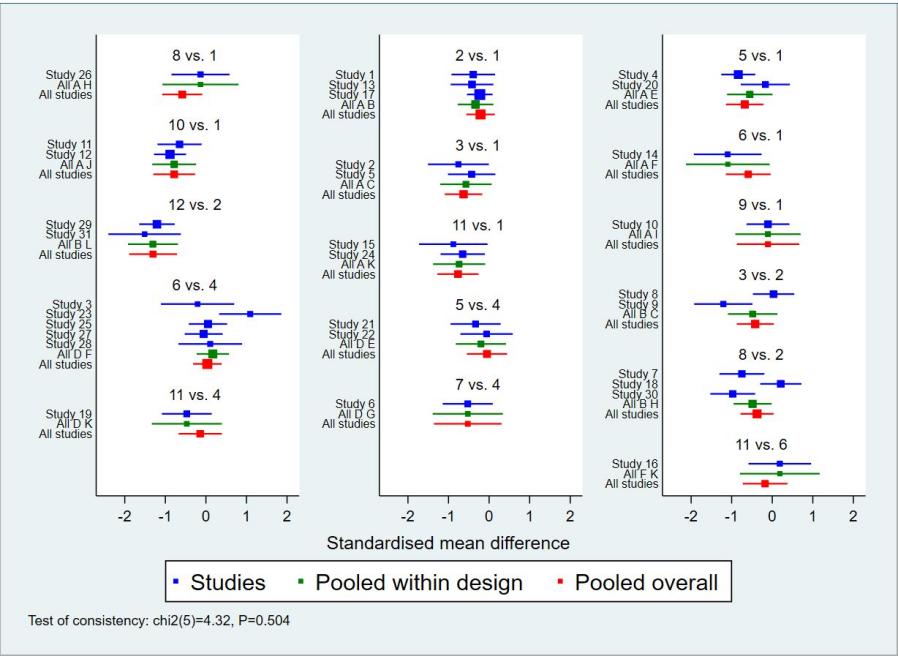


Note:1.CT;2.AE;3.Qigong;4.AE+SE;5.AE+SE+Supervise;6.SE;7.HS;8.AE+PT;9.RE;10.Yoga;11.Pilates;12.AE+Pilates

CT:Conventional Therapy; AE:Aerobic Exercises; RE: Resistance Exercise; PT:Physical Therapy; SE:Stretching Exercise; HS:Hippotherapy Simulation

**Figure S9** **A pairwise comparison of the BASMI scores was conducted between all exercise interventions and CT.**
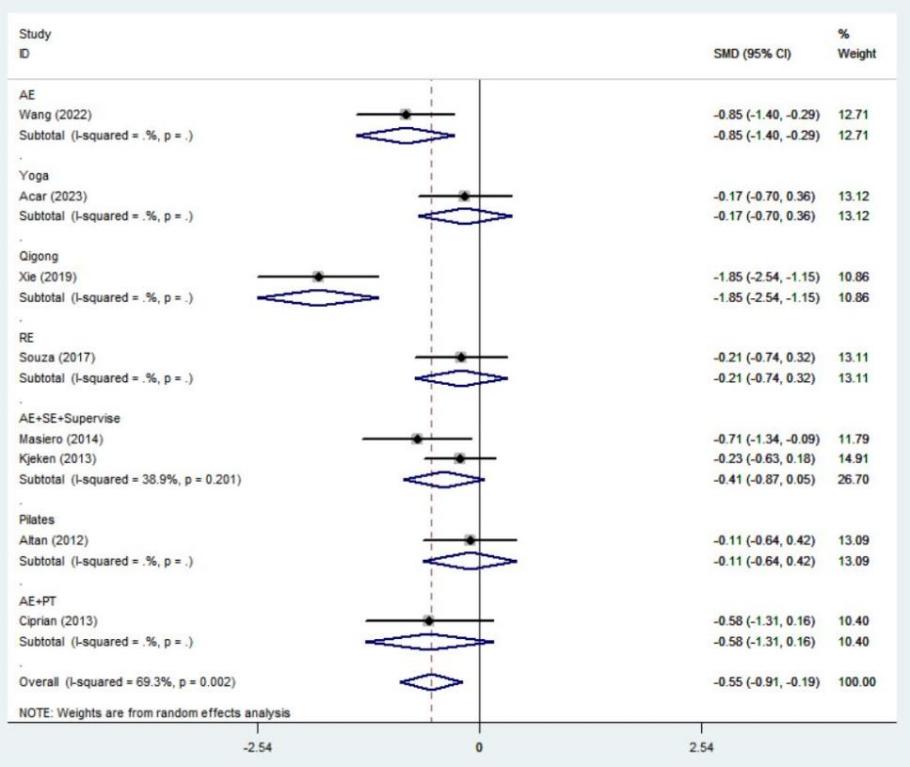


CT:Conventional Therapy; AE:Aerobic Exercises; RE: Resistance Exercise; PT:Physical Therapy; SE:Stretching Exercise; HS:Hippotherapy Simulation

**Figure S10 Non-concordance testing for BASMI scores.**
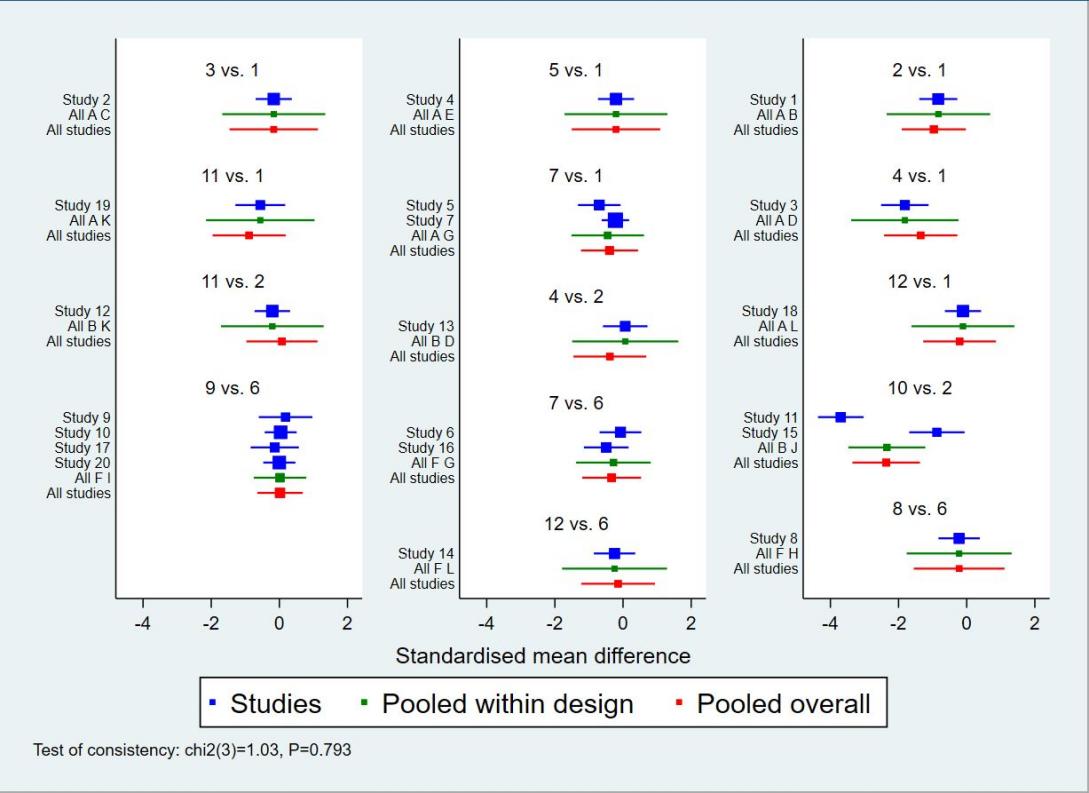


Note:1.CT;2.AE;3.Yoga;4.Qigong;5.RE;6.AE+SE;7.AE+SE+Supervise;8.HS;9.SE;10.AE+Pilates;11.AE+PT;12.Pilates

CT:Conventional Therapy; AE:Aerobic Exercises; RE: Resistance Exercise; PT:Physical Therapy; SE:Stretching Exercise; HS:Hippotherapy Simulation

**Figure S11 BASMI score, pairwise comparison of AE and Qigong with CT**


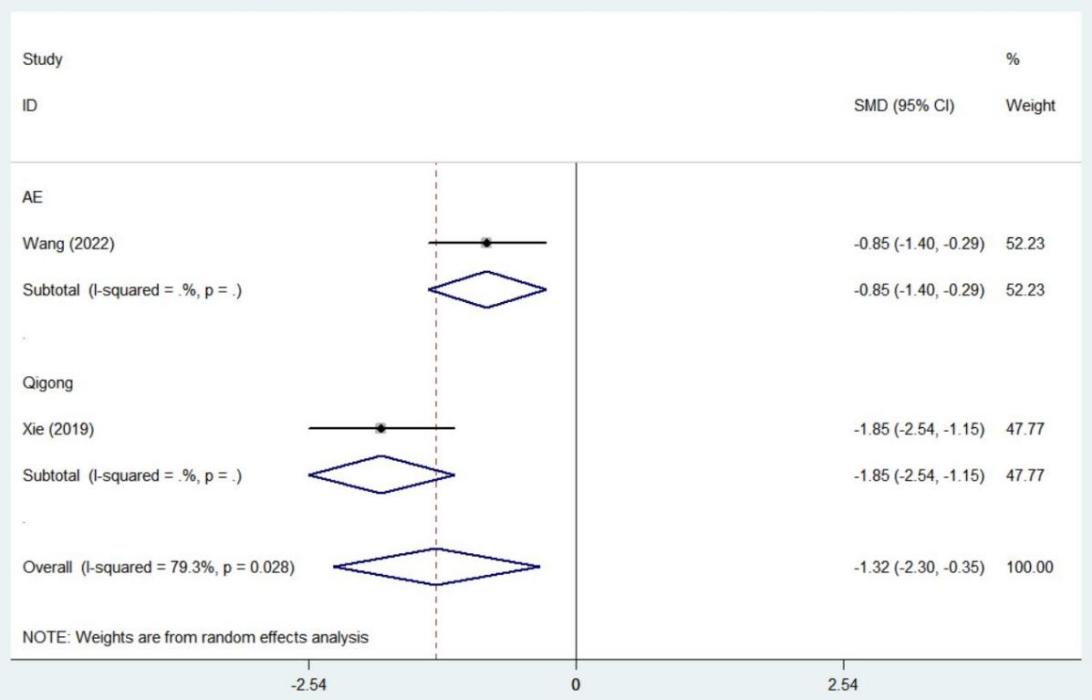


CT:Conventional Therapy; AE:Aerobic Exercises.

**Figure S12 Sensitivity analysis of pairwise comparison between AE, Qigong, and CT.**


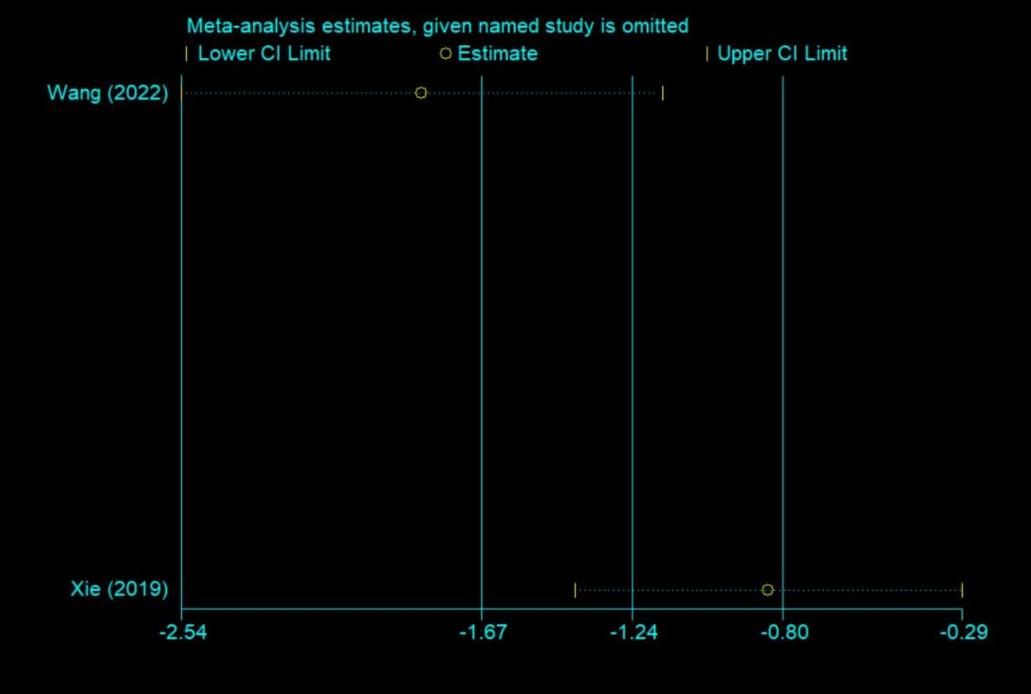


CT:Conventional Therapy; AE:Aerobic Exercises.

**Figure S13 A pairwise comparison of the CE was conducted between all exercise interventions and CT.**


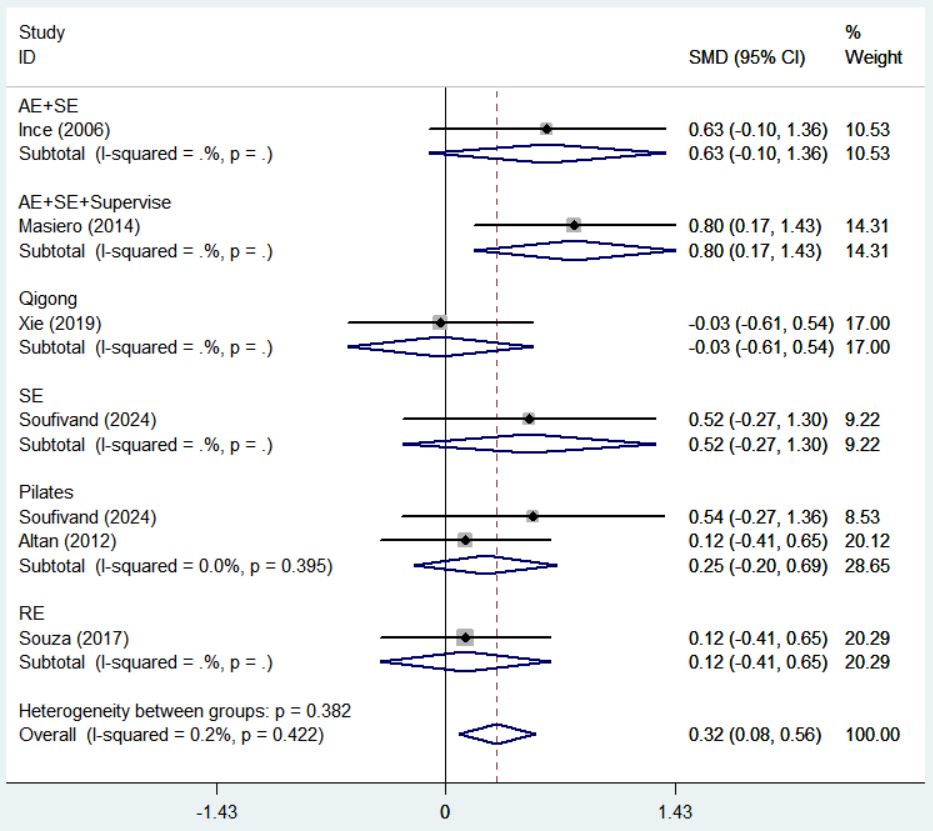


CT:Conventional Therapy; AE:Aerobic Exercises; RE: Resistance Exercise; PT:Physical Therapy; SE:Stretching Exercise.

**Figure S14 Non-concordance testing for CE.**
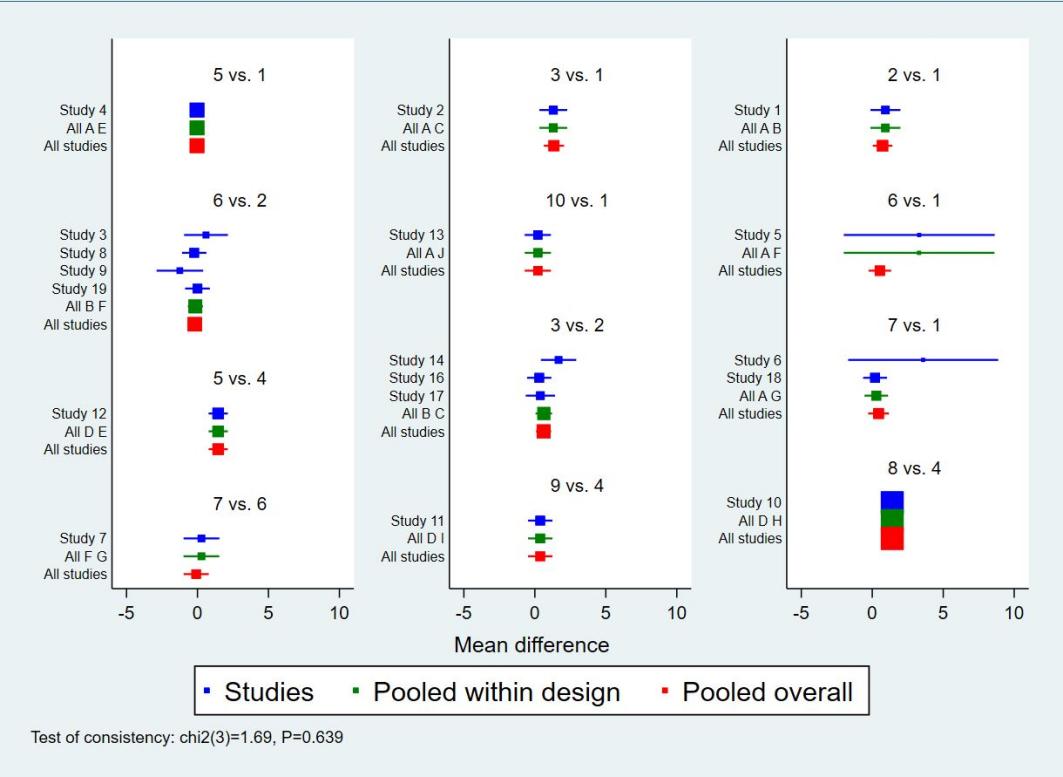


Note:1.CT;2.AE;3.Yoga;4.Qigong;5.RE;6.AE+SE;7.AE+SE+Supervise;8.HS;9.SE;10.AE+Pilates;11.AE+PT;12.Pilates

CT:Conventional Therapy; AE:Aerobic Exercises; RE: Resistance Exercise; PT:Physical Therapy; SE:Stretching Exercise; HS:Hippotherapy Simulation

**Figure S15 BASFI network diagram after removing high-risk studies.**


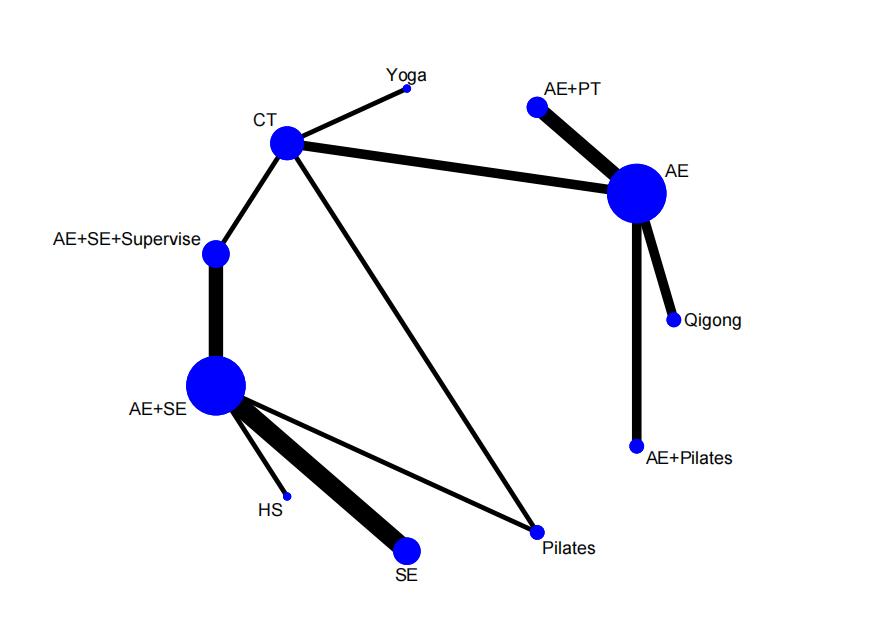


CT:Conventional Therapy; AE:Aerobic Exercises; RE: Resistance Exercise; PT:Physical Therapy; SE:Stretching Exercise; HS:Hippotherapy Simulation

**Figure S16 BASDAI network diagram after removing high-risk studies.**


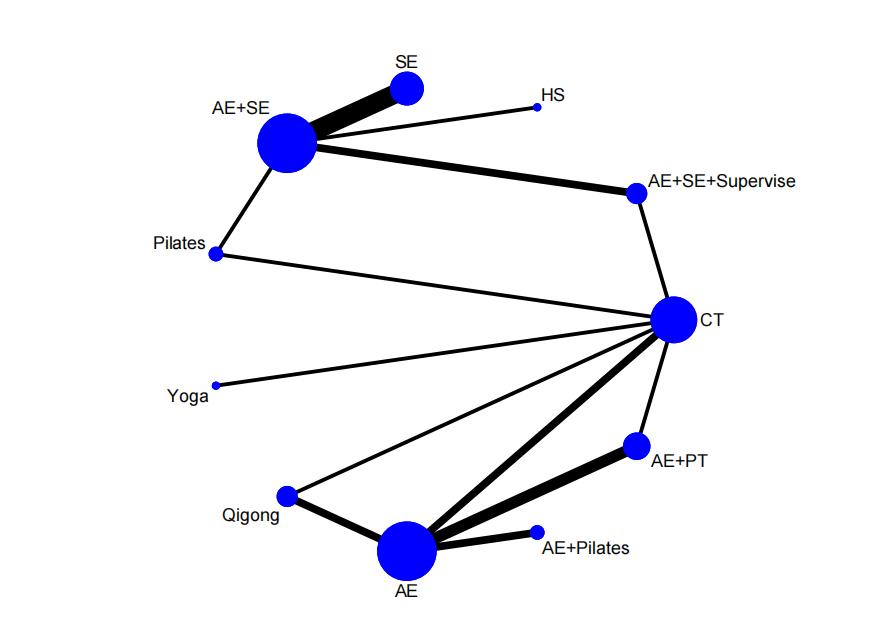


CT:Conventional Therapy; AE:Aerobic Exercises; PT:Physical Therapy; SE:Stretching Exercise; HS:Hippotherapy Simulation

**Figure S17 BASMI network diagram after removing high-risk studies.**


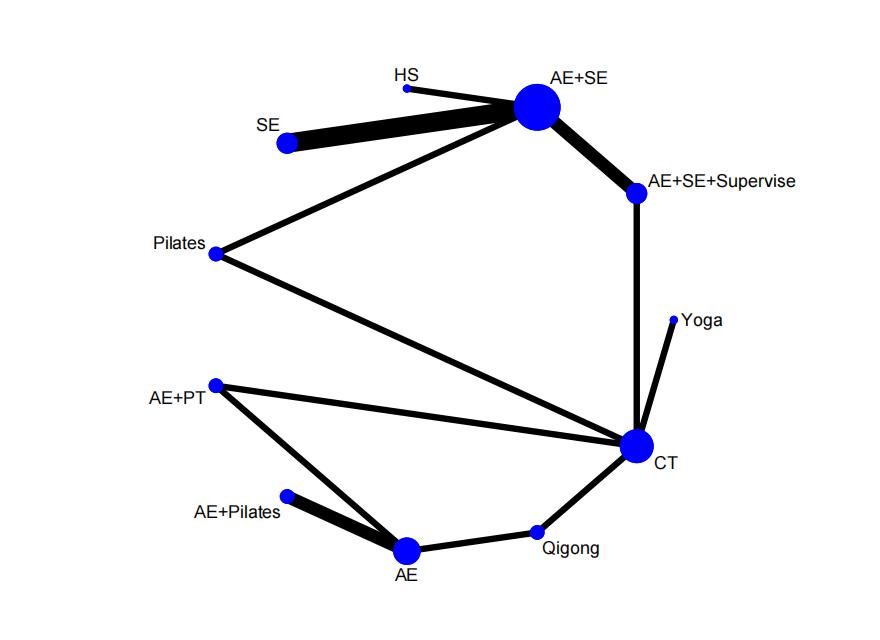


CT:Conventional Therapy; AE:Aerobic Exercises; PT:Physical Therapy; SE:Stretching Exercise; HS:Hippotherapy Simulation.

**Figure S18 BASFI score SUCRA chart for high-risk research removal.**


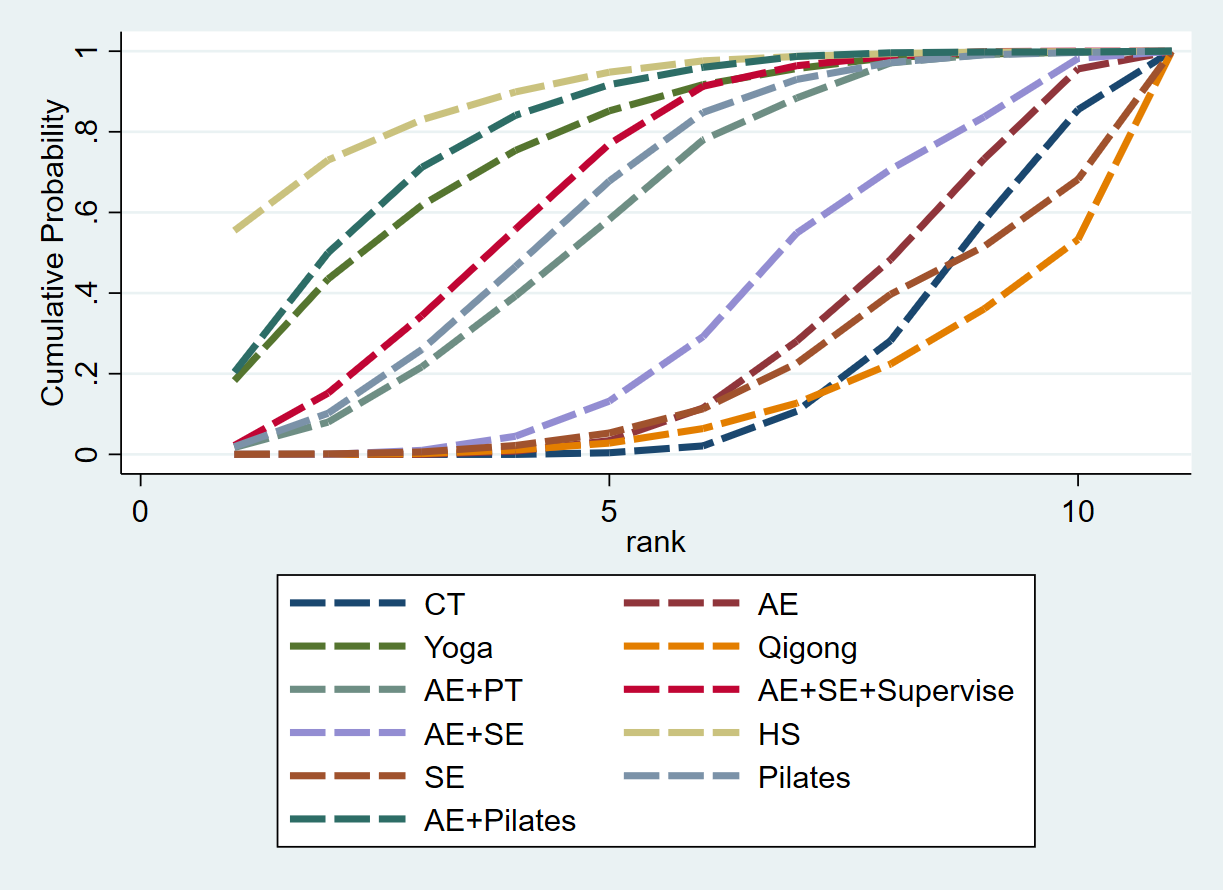


CT:Conventional Therapy; AE:Aerobic Exercises; RE: Resistance Exercise; PT:Physical Therapy; SE:Stretching Exercise; HS:Hippotherapy Simulation.

**Figure S19 BASDAI score SUCRA chart for high-risk research removal.**


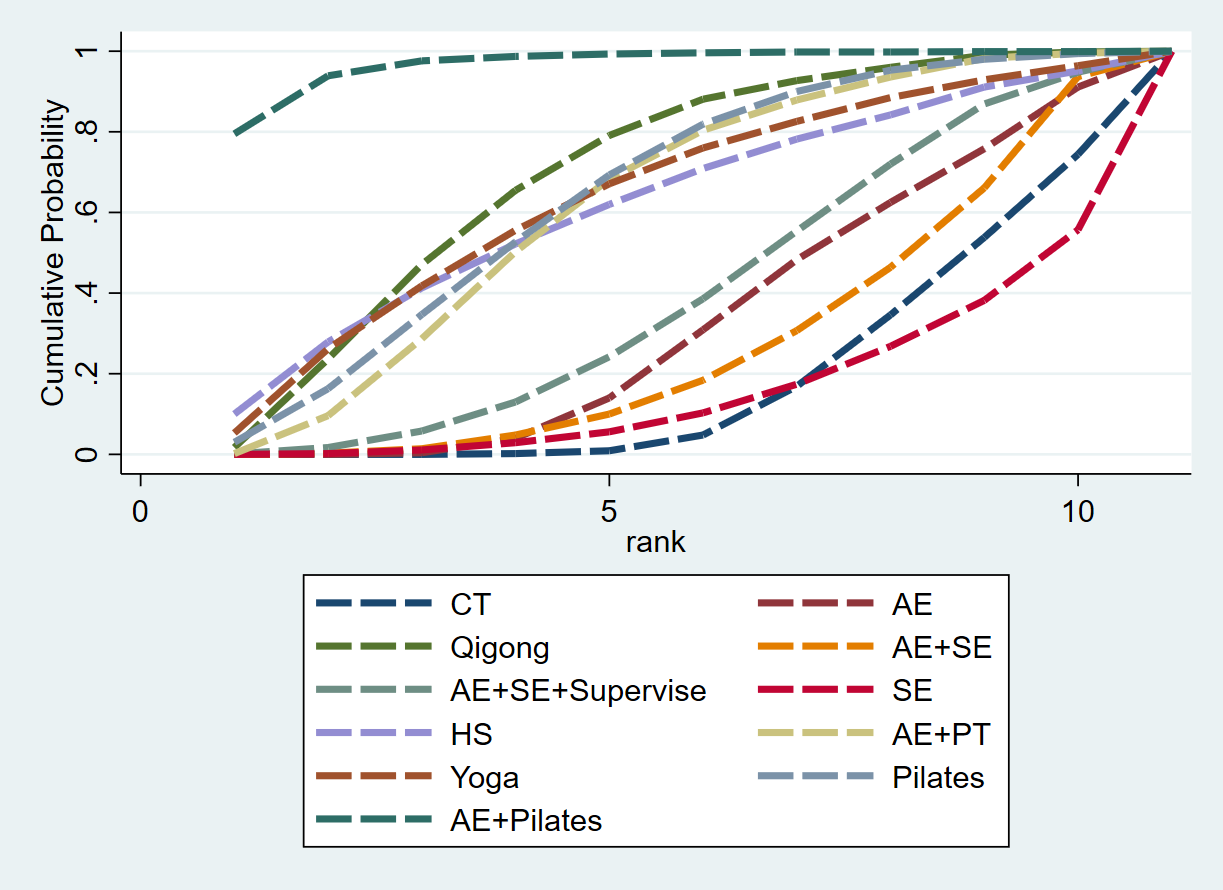


CT:Conventional Therapy; AE:Aerobic Exercises; PT:Physical Therapy; SE:Stretching Exercise; HS:Hippotherapy Simulation.

**Figure S20 BASDAI score SUCRA chart for high-risk research removal.**


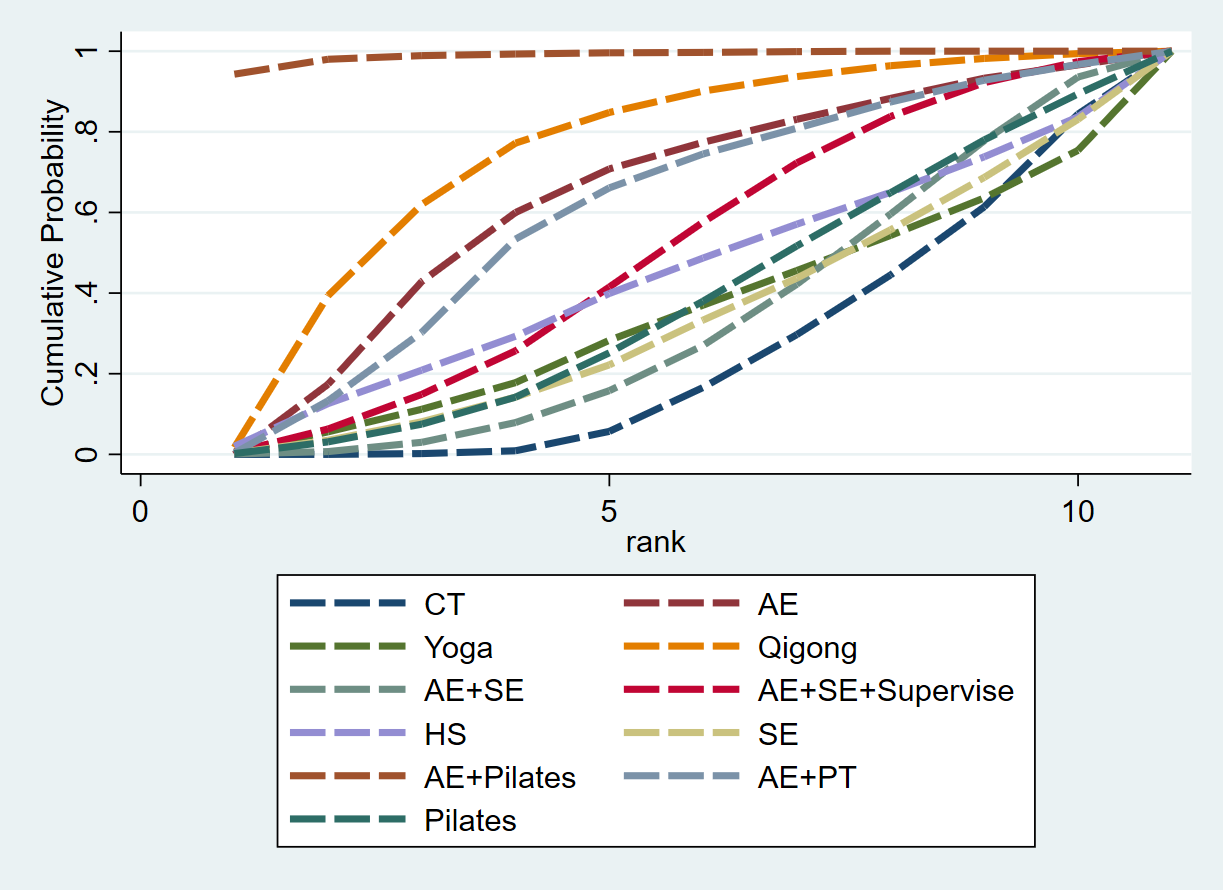


CT:Conventional Therapy; AE:Aerobic Exercises; PT:Physical Therapy; SE:Stretching Exercise; HS:Hippotherapy Simulation.

**Figure S21 BASFI scoring funnel plot for removing high-risk studies.**


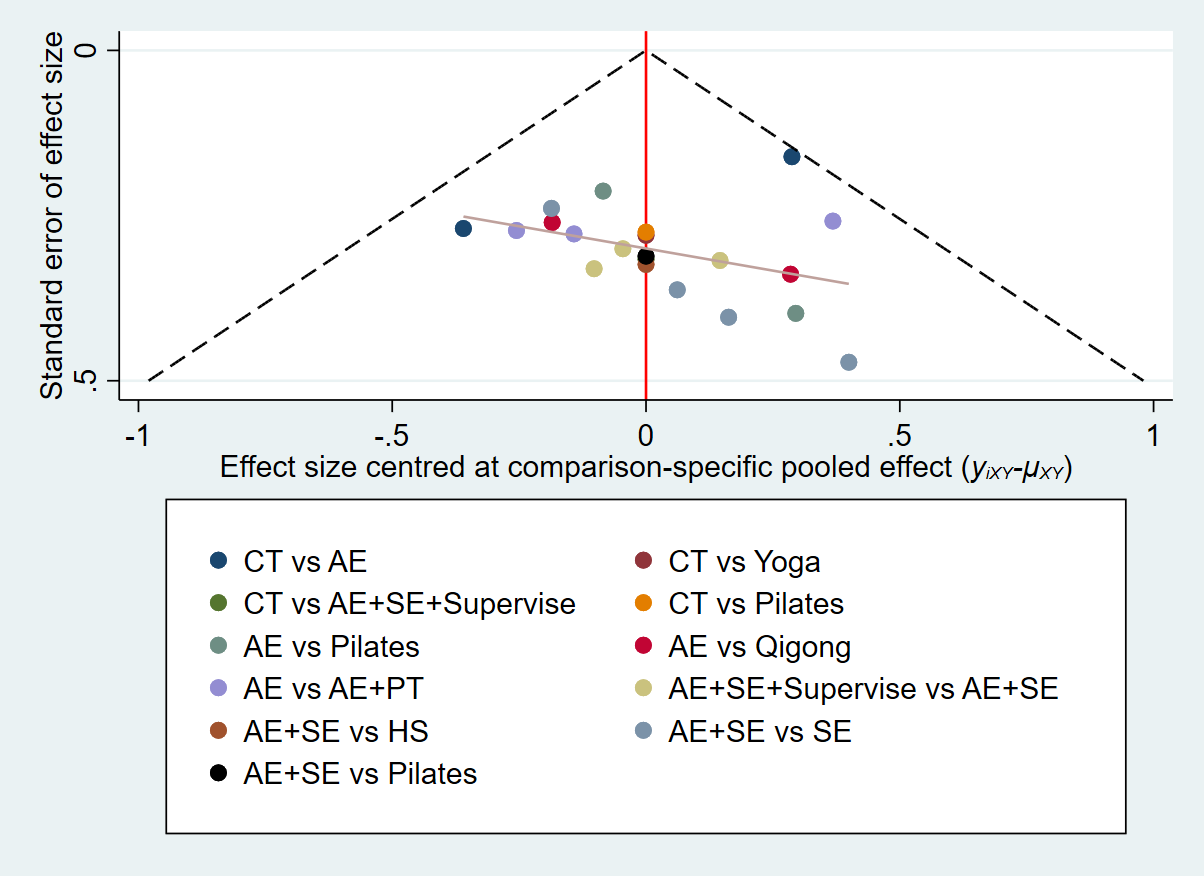


CT:Conventional Therapy; AE:Aerobic Exercises;PT:Physical Therapy; SE:Stretching Exercise; HS:Hippotherapy Simulation

**Figure S22 BASDAI scoring funnel plot for removing high-risk studies.**


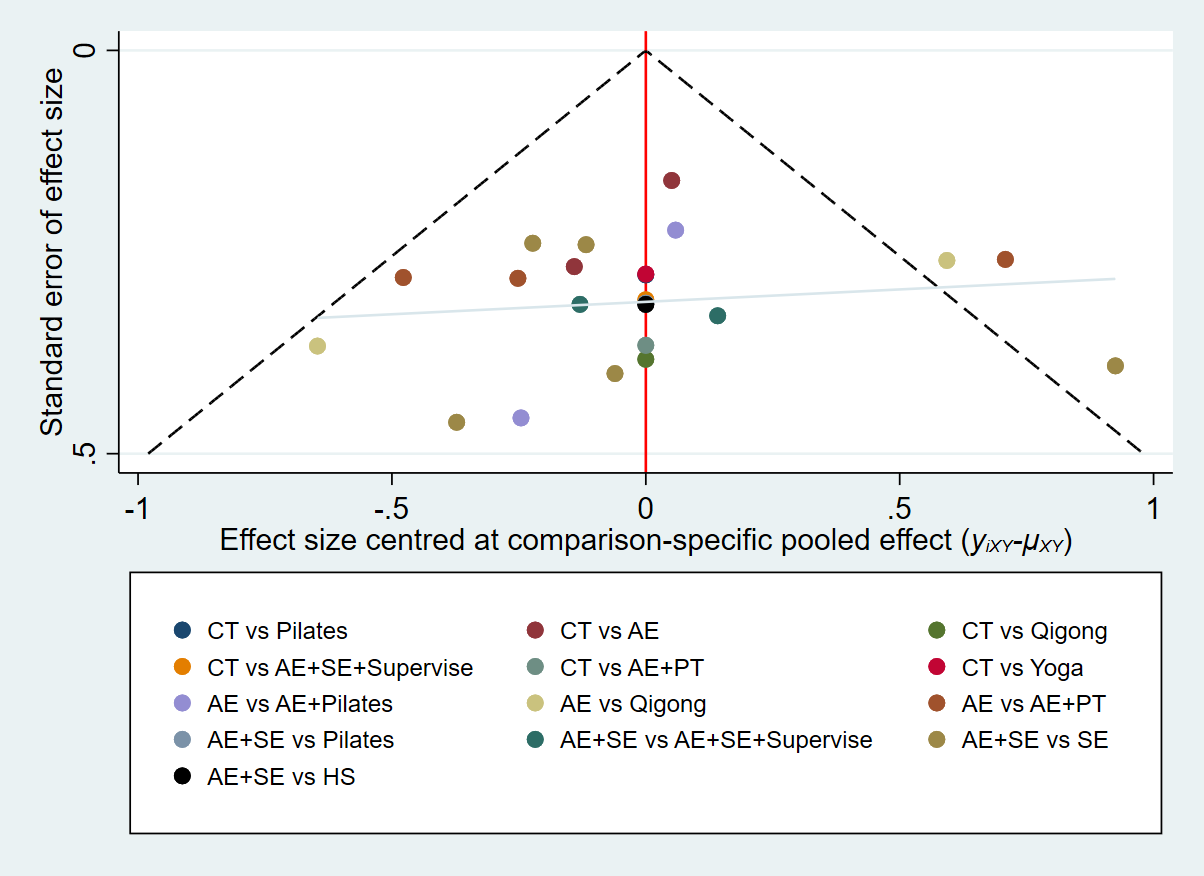


CT:Conventional Therapy; AE:Aerobic Exercises;PT:Physical Therapy; SE:Stretching Exercise; HS:Hippotherapy Simulation

**Figure S23 BASMI scoring funnel plot for removing high-risk studies.**


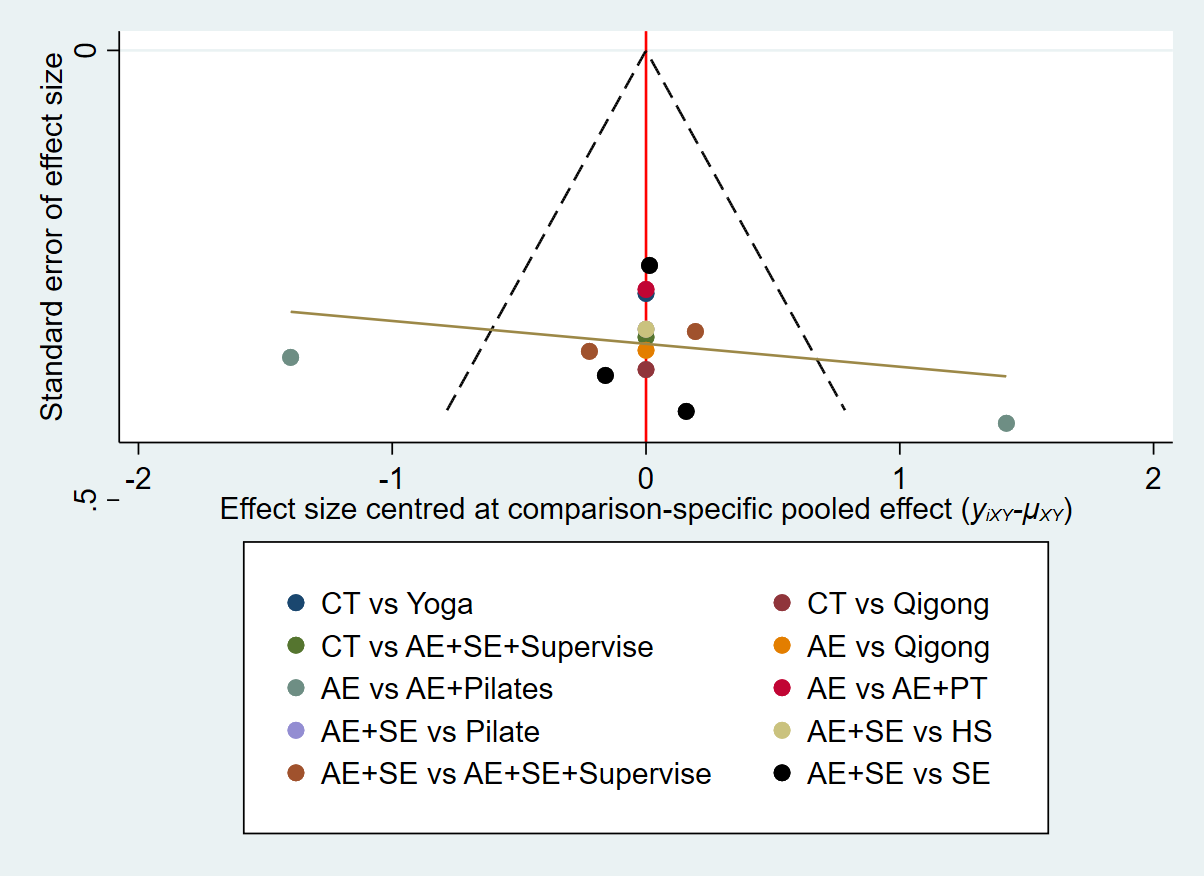


CT:Conventional Therapy; AE:Aerobic Exercises;PT:Physical Therapy; SE:Stretching Exercise; HS:Hippotherapy Simulation

**Figure S24 CE network diagram after removing high-risk studies.**


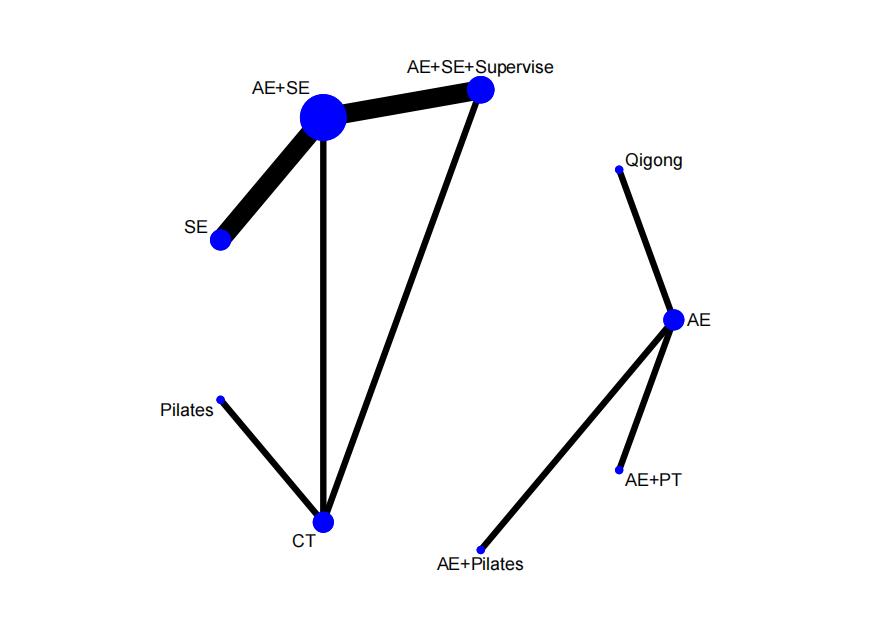


CT:Conventional Therapy; AE:Aerobic Exercises; PT:Physical Therapy; SE:Stretching Exercise.

**Figure S25 A pairwise comparison of the CE was conducted between all exercise interventions and CT(removal of High Risk Studies).**
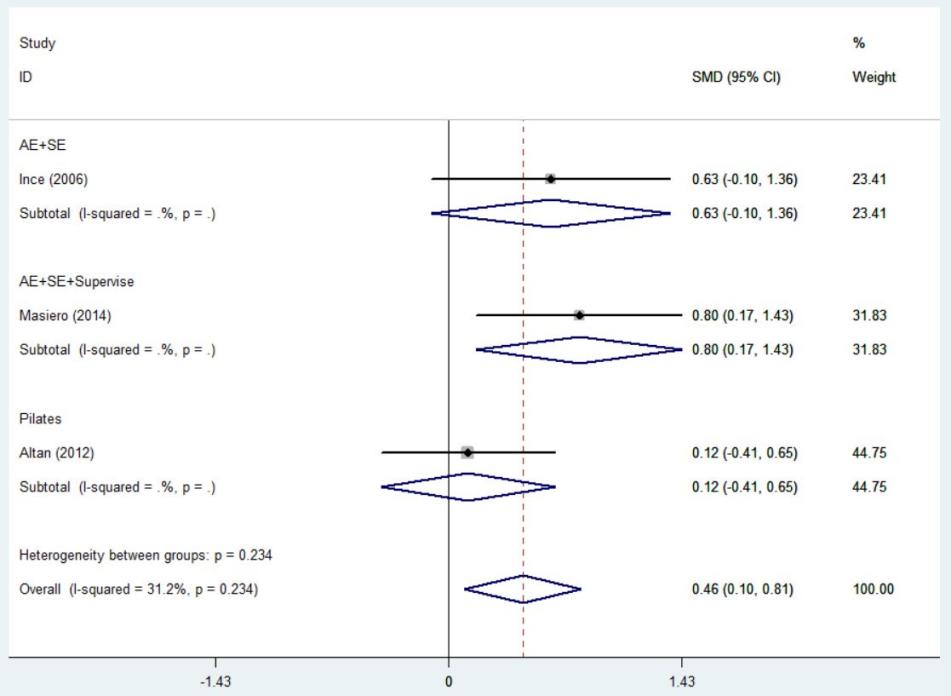


CT:Conventional Therapy; AE:Aerobic Exercises; SE:Stretching Exercise; CE:

**Figure S26 A direct pairwise comparison was conducted between SE and AE, as well as between AE+SE+Supervise and AE(removal of High Risk Studies).**


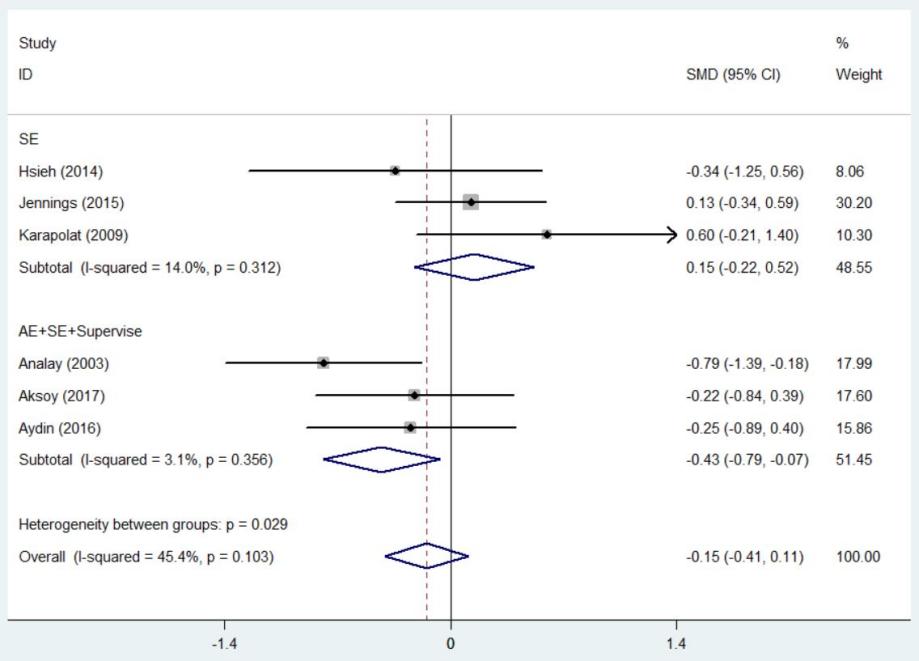


CT:Conventional Therapy; AE:Aerobic Exercises; SE:Stretching Exercise.

**Figure S27 A pairwise comparison was conducted between AE+Pilates, AE+PT, and Qigong, each directly compared with AE(removal of High Risk Studies).**


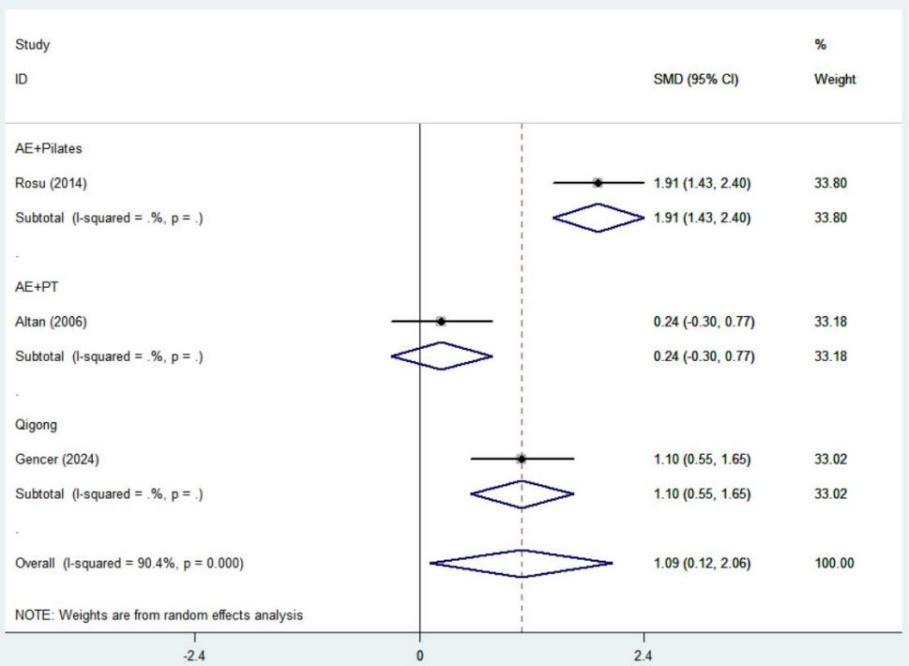


CT:Conventional Therapy; AE:Aerobic Exercises; PT:Physical Therapy.

**Figure S28 Sensitivity analysis of pairwise comparison between AE+Pilates, AE+PT, Qigong and CT(removal of High Risk Studies).**


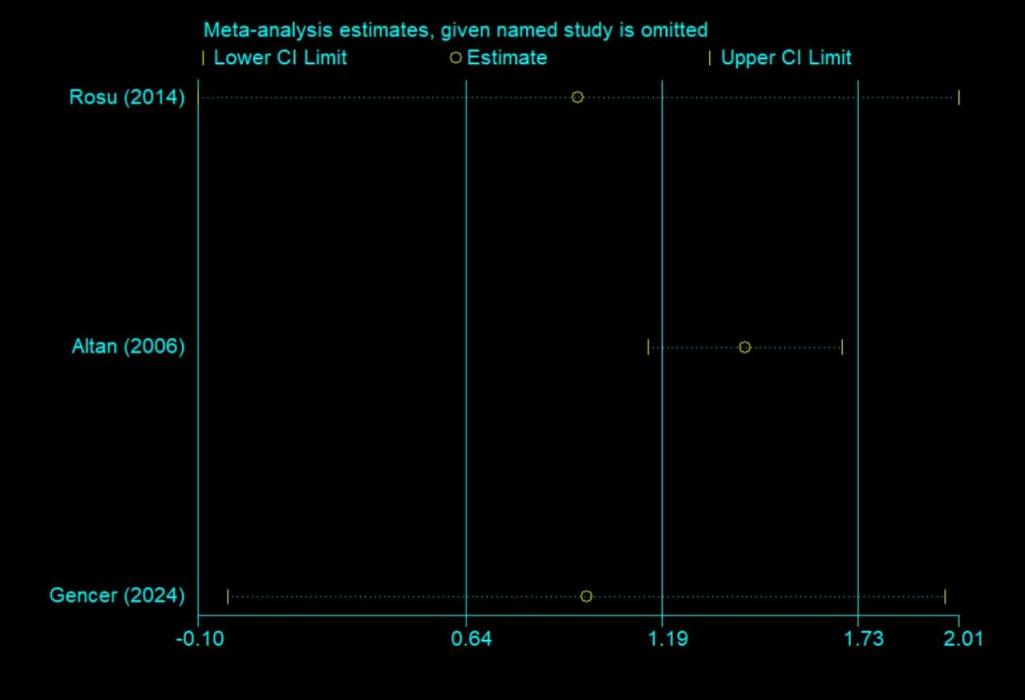


**Figure S29 Dose changes of AE reducing BASFI score.**


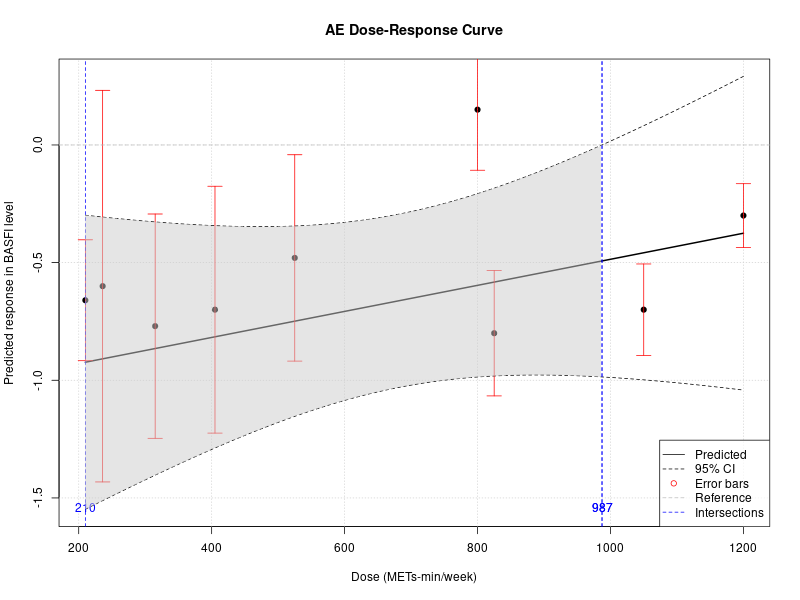


The nodes represent the original dataset. Shaded areas represent signifcant 95% credible intervals(CI). The blue dashed lines represent the beginning and end of the signifcant 95% CI, and the blue numbers represent the specifc dose values at the beginning and end (metabolic equivalents [METs]-minutes [min]/week).

AE:Aerobic Exercises

**Figure S30 Dose changes of AE+Pilates reducing BASFI score.**


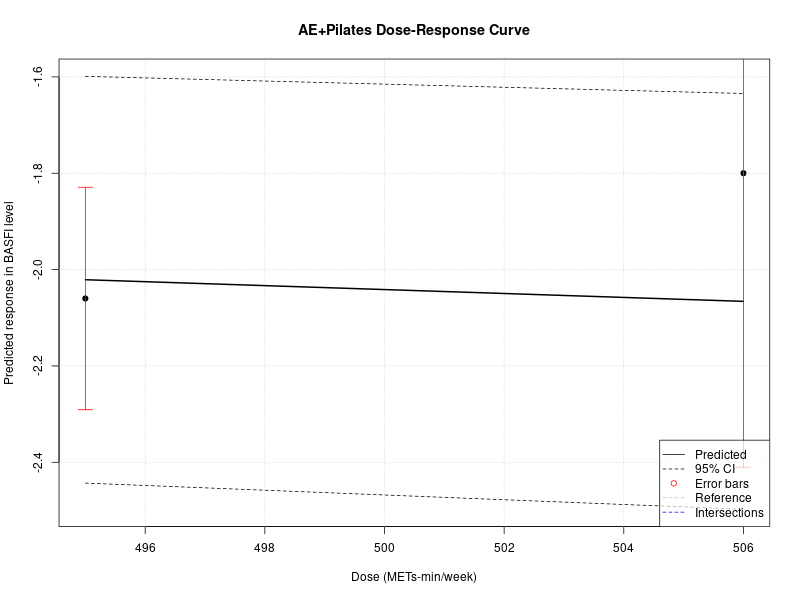


AE:Aerobic Exercises

**Figure S31 Dose changes of AE+PT reducing BASFI score.**


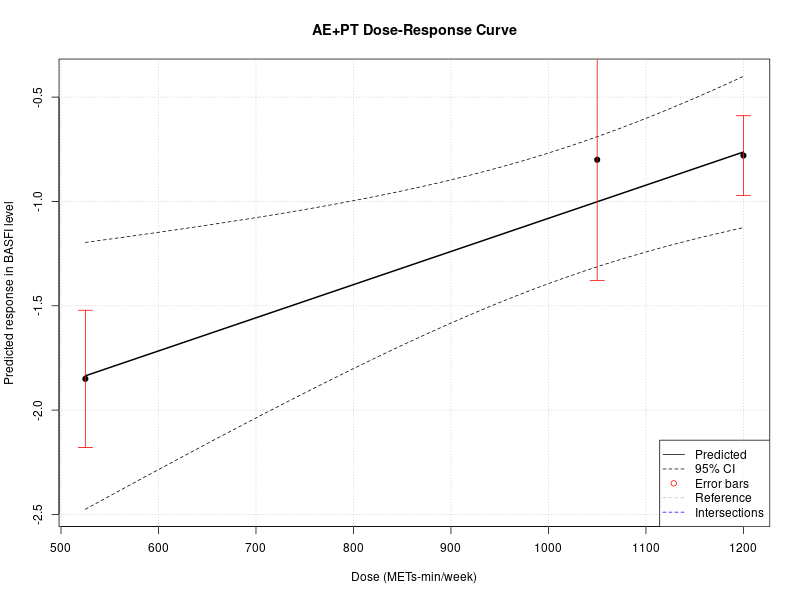


AE:Aerobic Exercises; PT:Physical Therapy.

**Figure S32 Dose changes of AE+SE reducing BASFI score.**


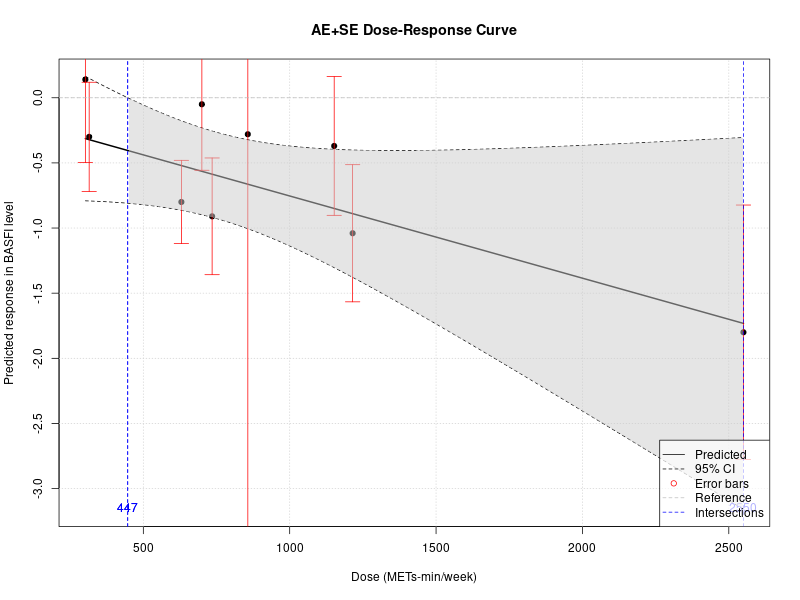


The nodes represent the original dataset. Shaded areas represent signifcant 95% credible intervals(CI). The blue dashed lines represent the beginning and end of the signifcant 95% CI, and the blue numbers represent the specifc dose values at the beginning and end (metabolic equivalents [METs]-minutes [min]/week).

AE:Aerobic Exercises; SE:Stretching Exercise

**Figure S33 Dose changes of AE+SE+Supervise reducing BASFI score.**


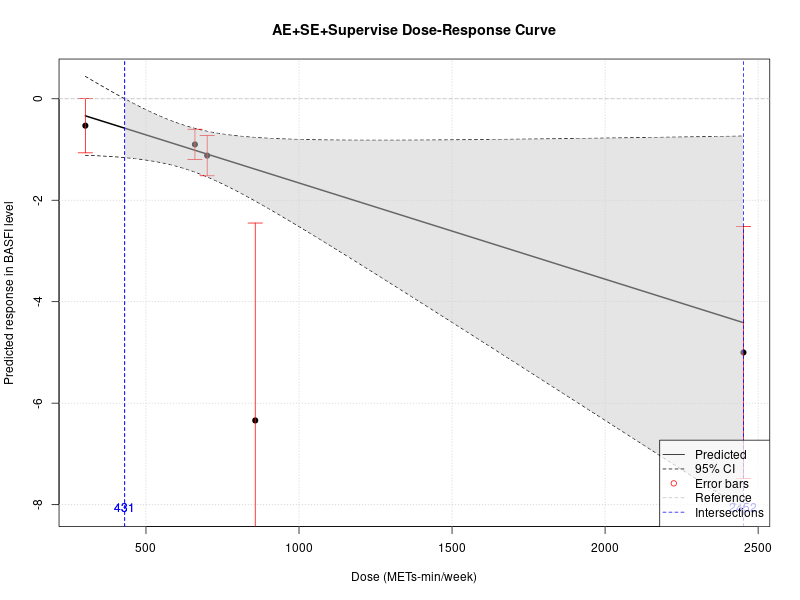


The nodes represent the original dataset. Shaded areas represent signifcant 95% credible intervals(CI). The blue dashed lines represent the beginning and end of the signifcant 95% CI, and the blue numbers represent the specifc dose values at the beginning and end (metabolic equivalents [METs]-minutes [min]/week).

AE:Aerobic Exercises; SE:Stretching Exercise

**Figure S34 Dose changes of Pilates reducing BASFI score.**


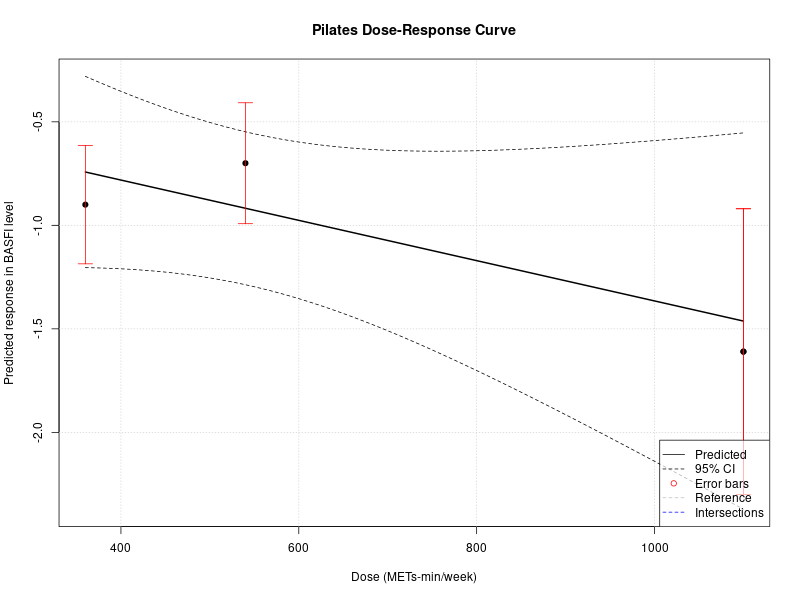


**Figure S35 Dose changes of Qigong reducing BASFI score.**


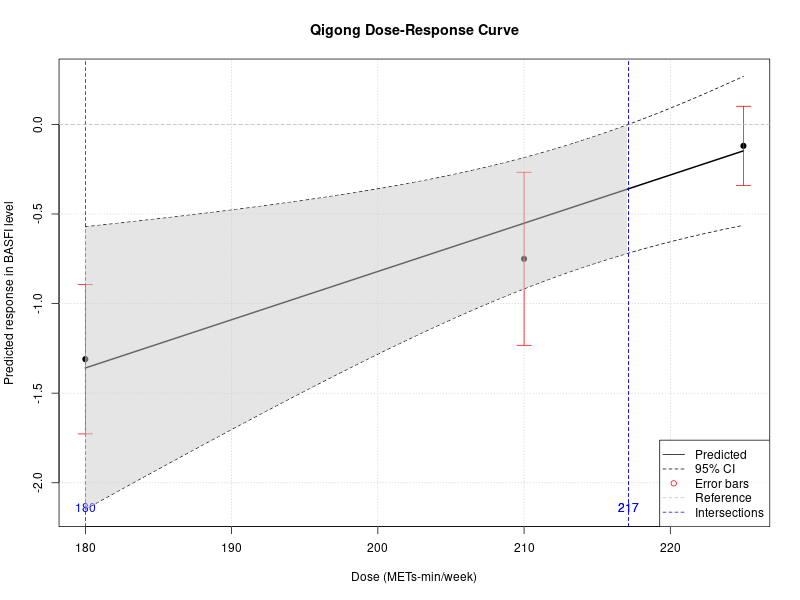


The nodes represent the original dataset. Shaded areas represent signifcant 95% credible intervals(CI). The blue dashed lines represent the beginning and end of the signifcant 95% CI, and the blue numbers represent the specifc dose values at the beginning and end (metabolic equivalents [METs]-minutes [min]/week).

**Figure S36 Dose changes of SE reducing BASFI score.**


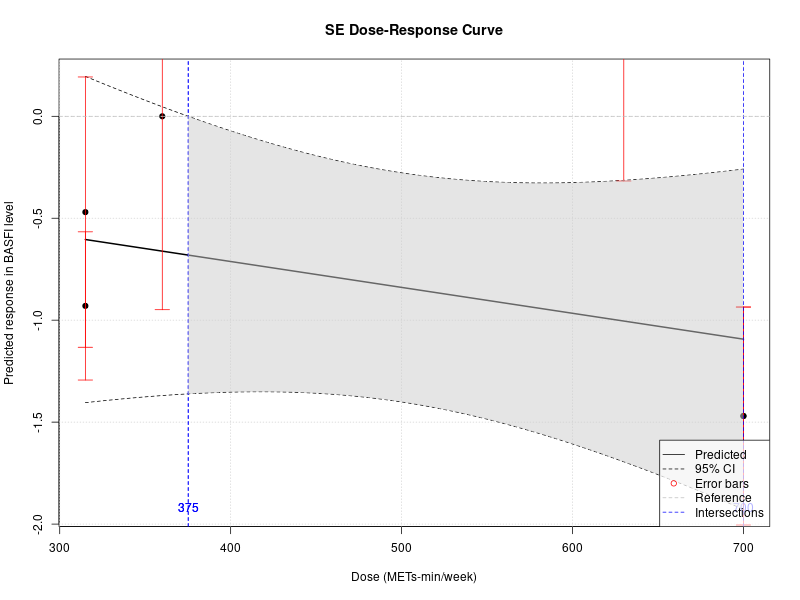


The nodes represent the original dataset. Shaded areas represent signifcant 95% credible intervals(CI). The blue dashed lines represent the beginning and end of the signifcant 95% CI, and the blue numbers represent the specifc dose values at the beginning and end (metabolic equivalents [METs]-minutes [min]/week).

SE:Stretching Exercise.

**Figure S37 Dose changes of Yoga reducing BASFI score.**


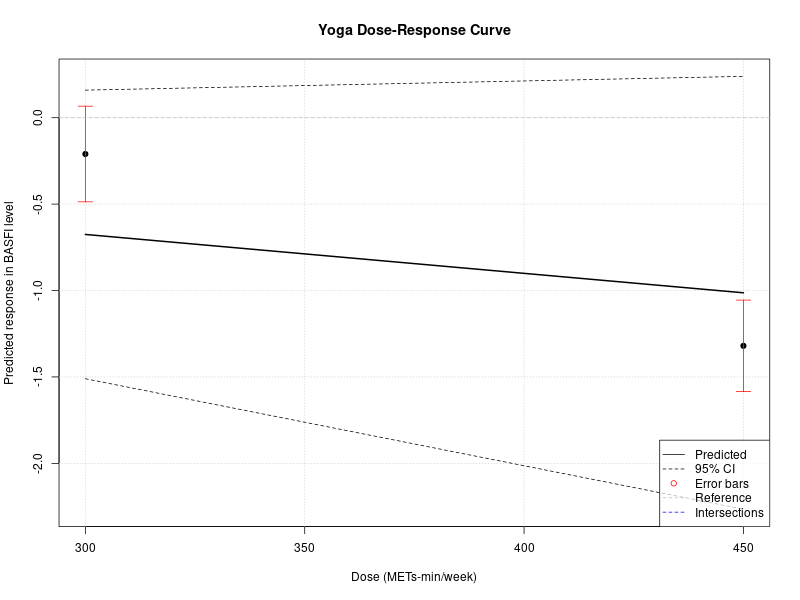


**Figure S38 Dose changes of AE reducing BASDAI score.**


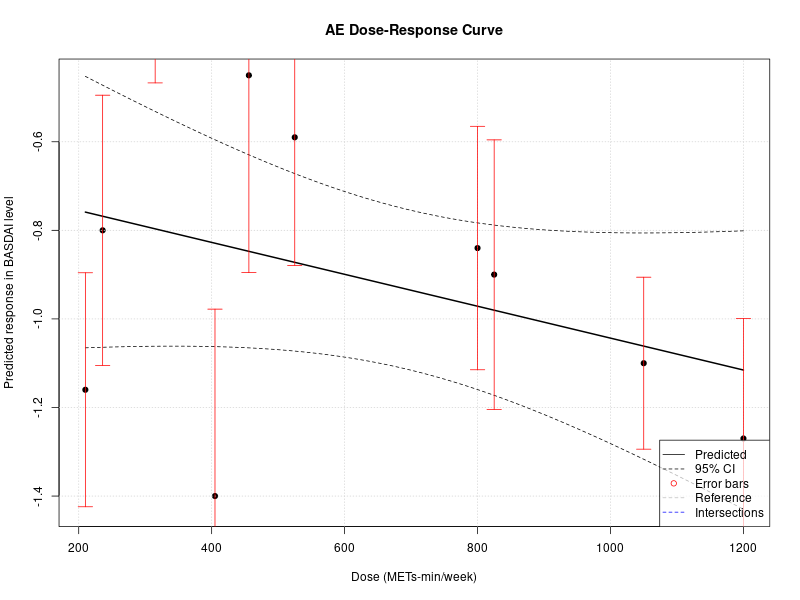


AE:Aerobic Exercises

**Figure S39 Dose changes of AE+Pilates reducing BASDAI score.**


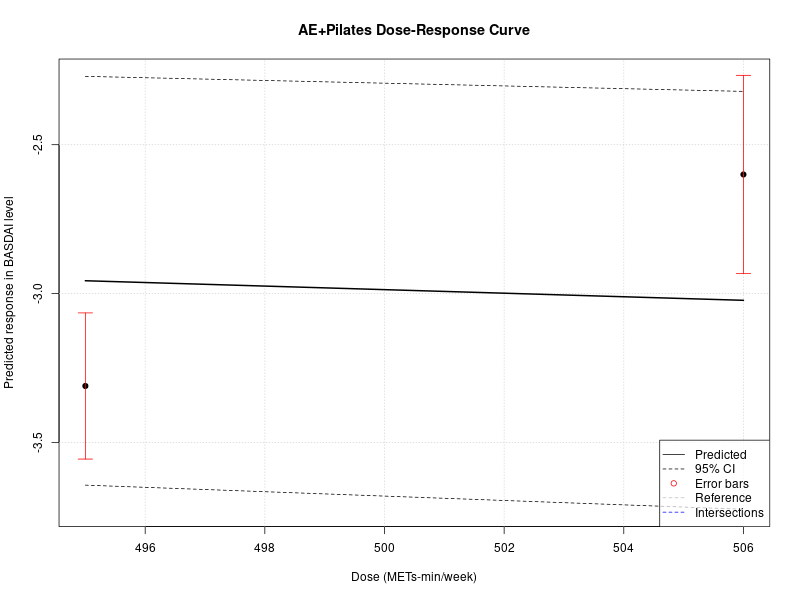


**Figure S40 Dose changes of AE+PT reducing BASDAI score.**


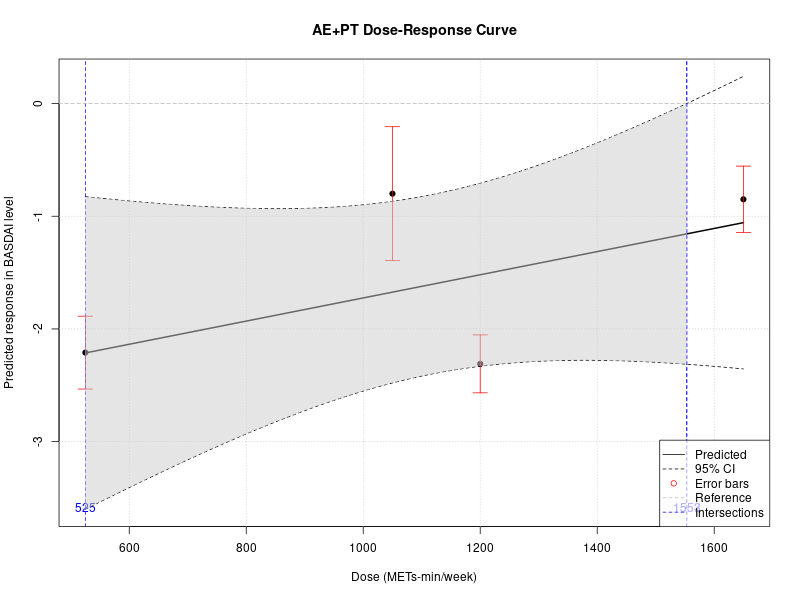


The nodes represent the original dataset. Shaded areas represent signifcant 95% credible intervals(CI). The blue dashed lines represent the beginning and end of the signifcant 95% CI, and the blue numbers represent the specifc dose values at the beginning and end (metabolic equivalents [METs]-minutes [min]/week).

AE:Aerobic Exercises; PT:Physical Therapy.

**Figure S41 Dose changes of AE+SE reducing BASDAI score.**


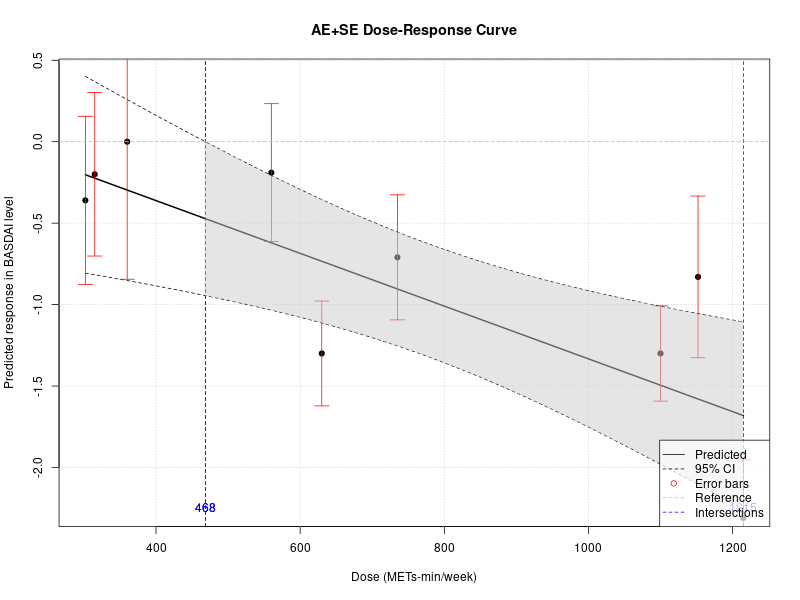


The nodes represent the original dataset. Shaded areas represent signifcant 95% credible intervals(CI). The blue dashed lines represent the beginning and end of the signifcant 95% CI, and the blue numbers represent the specifc dose values at the beginning and end (metabolic equivalents [METs]-minutes [min]/week).

AE:Aerobic Exercises;SE:Stretching Exercise.

**Figure S42 Dose changes of AE+SE+Supervise reducing BASDAI score.**


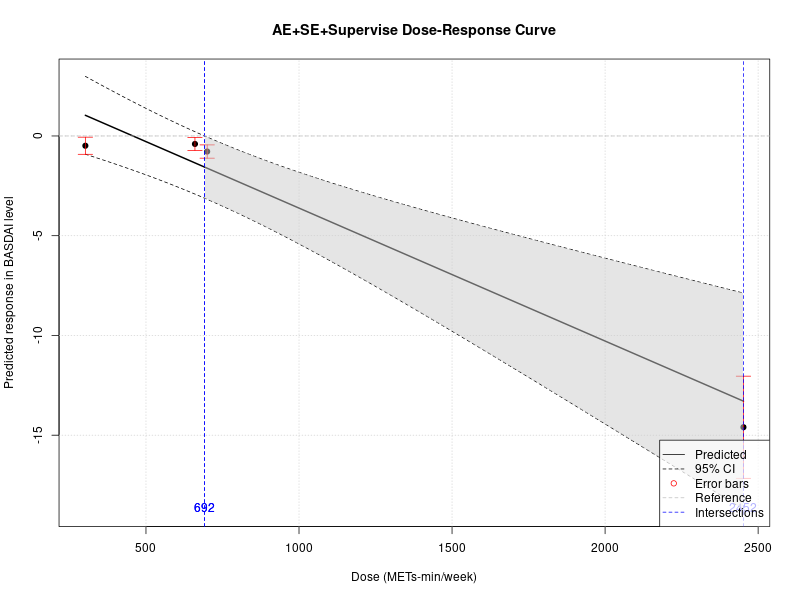


The nodes represent the original dataset. Shaded areas represent signifcant 95% credible intervals(CI). The blue dashed lines represent the beginning and end of the signifcant 95% CI, and the blue numbers represent the specifc dose values at the beginning and end (metabolic equivalents [METs]-minutes [min]/week).

AE:Aerobic Exercises; SE:Stretching Exercise

**Figure S43 Dose changes of Pilates reducing BASDAI score.**


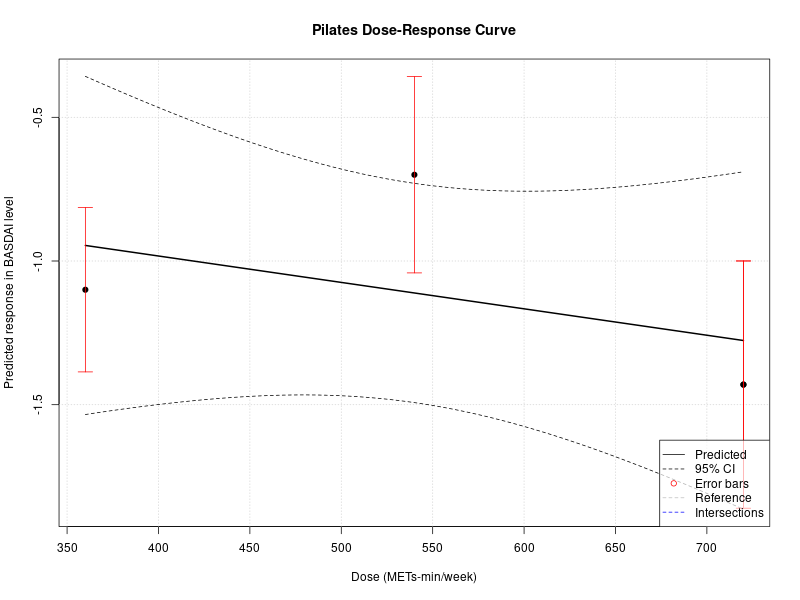


**Figure S44 Dose changes of Qigong reducing BASDAI score.**


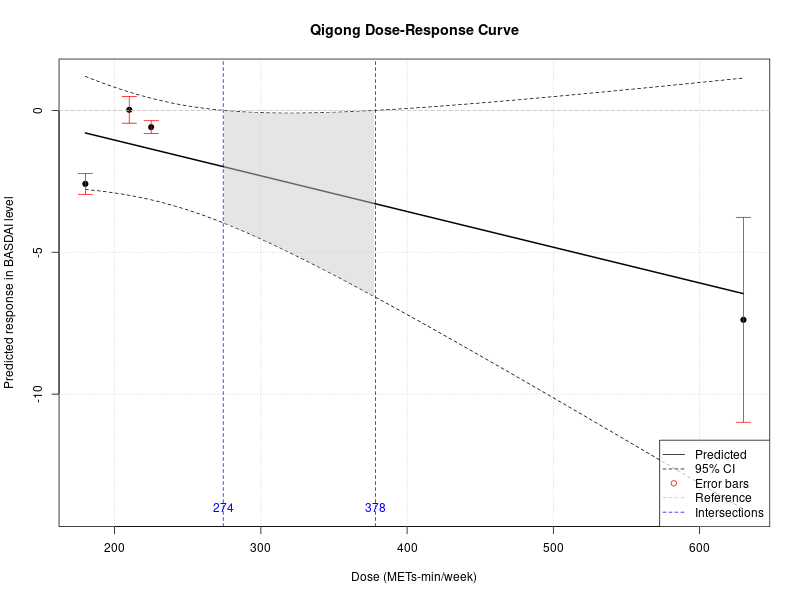


The nodes represent the original dataset. Shaded areas represent signifcant 95% credible intervals(CI). The blue dashed lines represent the beginning and end of the signifcant 95% CI, and the blue numbers represent the specifc dose values at the beginning and end (metabolic equivalents [METs]-minutes [min]/week).

**Figure S45 Dose changes of SE reducing BASDAI score.**


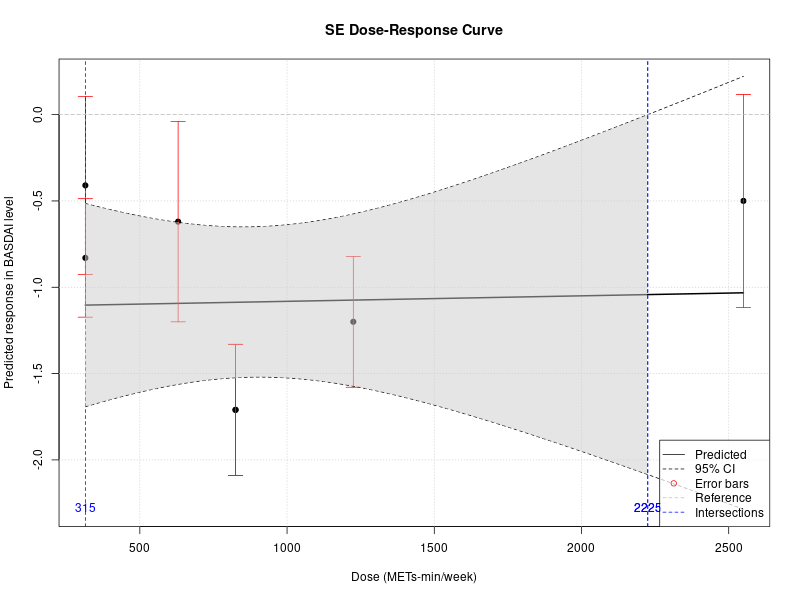


The nodes represent the original dataset. Shaded areas represent signifcant 95% credible intervals(CI). The blue dashed lines represent the beginning and end of the signifcant 95% CI, and the blue numbers represent the specifc dose values at the beginning and end (metabolic equivalents [METs]-minutes [min]/week).

SE:Stretching Exercise.

**Figure S46 Dose changes of SE reducing BASDAI score.**


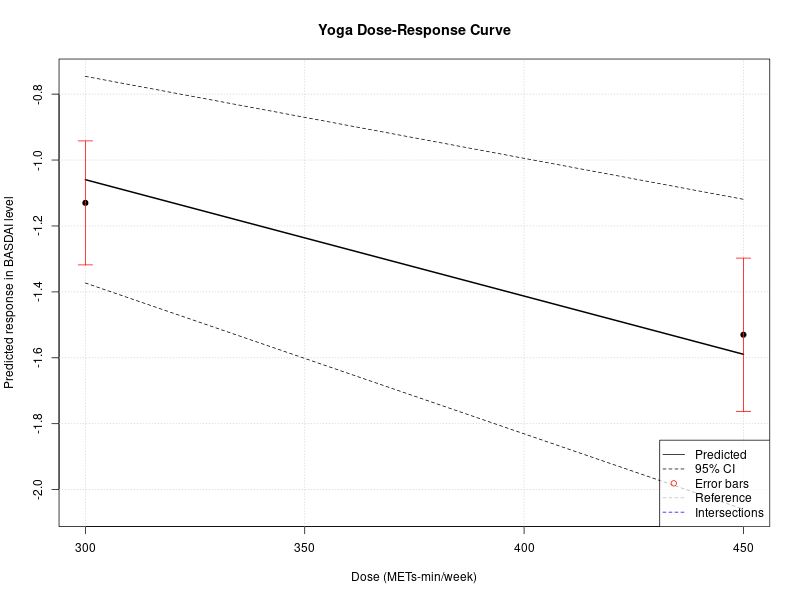


SE:Stretching Exercise.

**Figure S47 Dose changes of AE reducing BASMI score.**


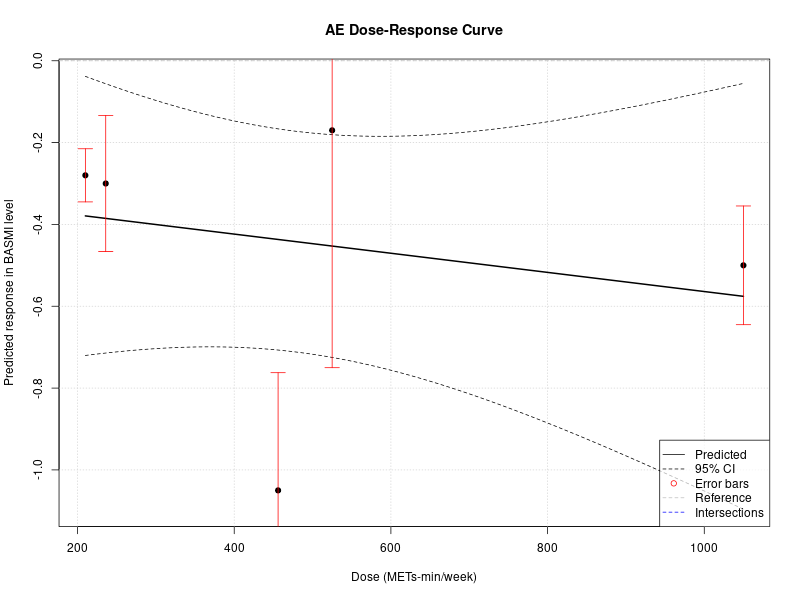


AE:Aerobic Exercises.

**Figure S48 Dose changes of AE+Pilates reducing BASMI score.**


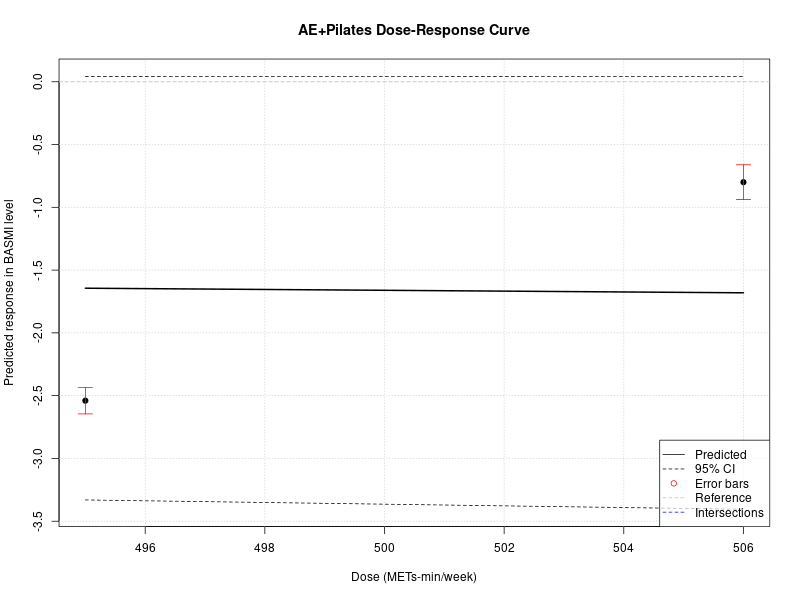


AE:Aerobic Exercises.

**Figure S49 Dose changes of AE+PT reducing BASMI score.**


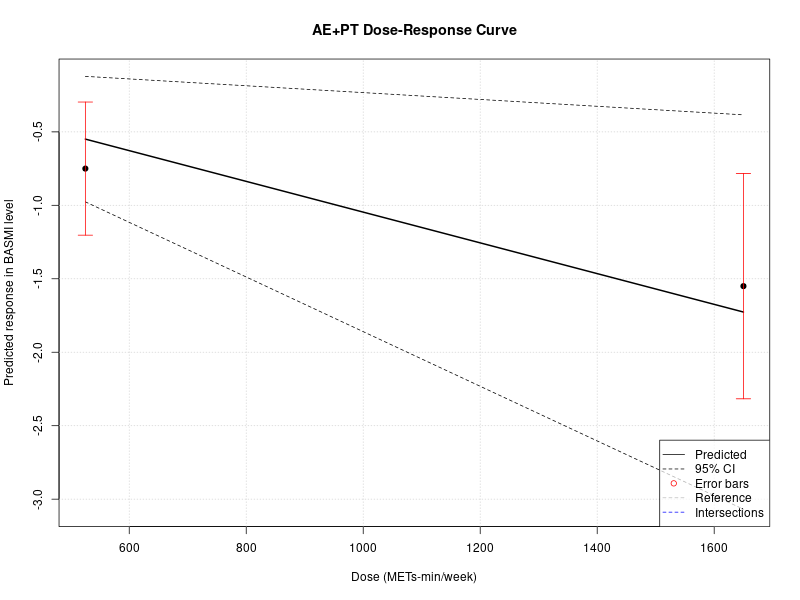


AE:Aerobic Exercises; PT:Physical Therapy.

**Figure S50 Dose changes of AE+SE reducing BASMI score.**


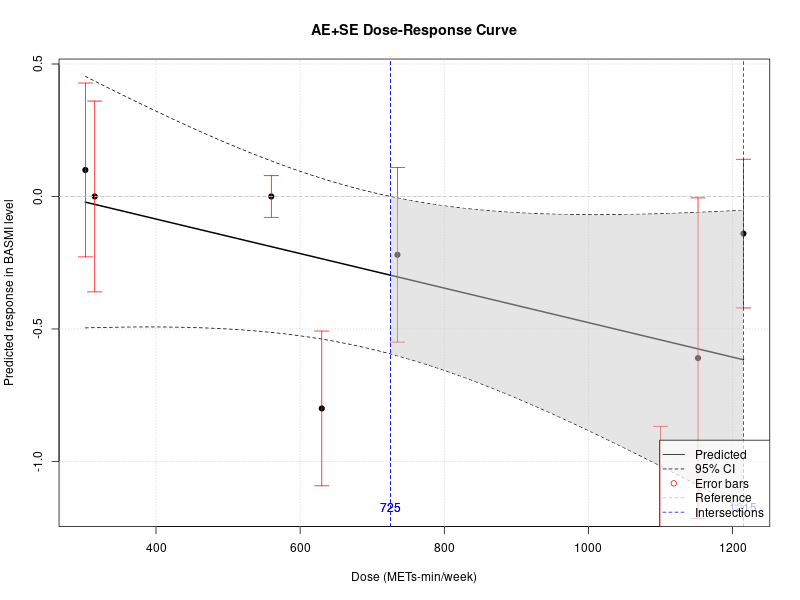


The nodes represent the original dataset. Shaded areas represent signifcant 95% credible intervals(CI). The blue dashed lines represent the beginning and end of the signifcant 95% CI, and the blue numbers represent the specifc dose values at the beginning and end (metabolic equivalents [METs]-minutes [min]/week).

AE:Aerobic Exercises; SE:Stretching Exercise.

**Figure S51 Dose changes of AE+SE+Supervise reducing BASMI score.**


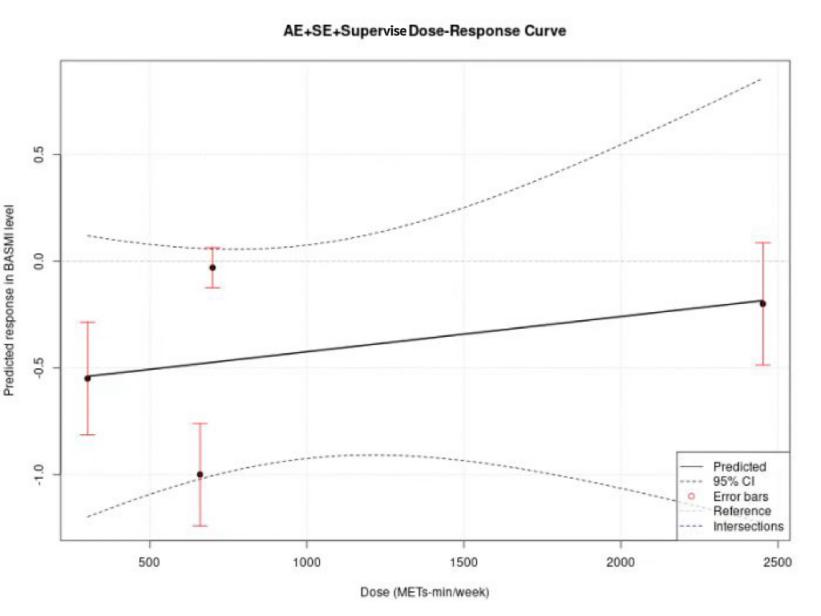


**Figure S52 Dose changes of Pilates reducing BASMI score.**


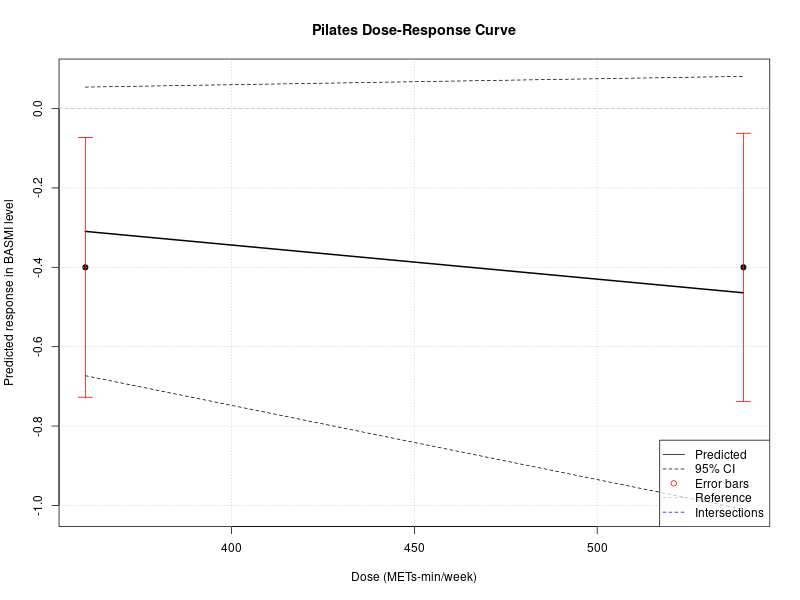


**Figure S53 Dose changes of Qigong reducing BASMI score.**


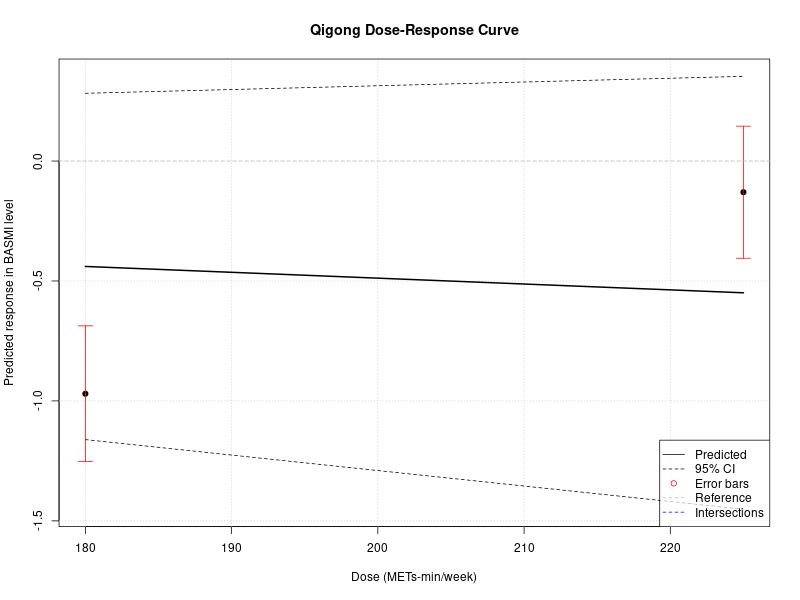


**Figure S54 Dose changes of SE reducing BASMI score.**


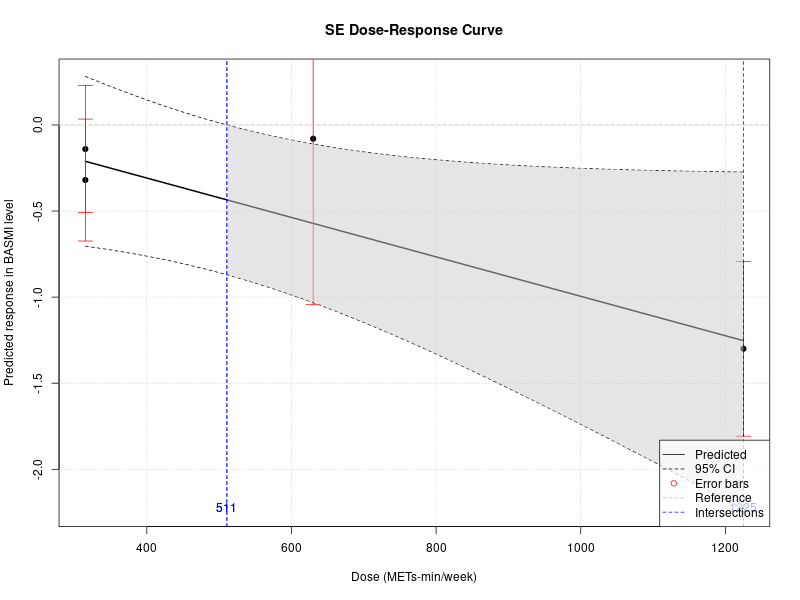


The nodes represent the original dataset. Shaded areas represent signifcant 95% credible intervals(CI). The blue dashed lines represent the beginning and end of the signifcant 95% CI, and the blue numbers represent the specifc dose values at the beginning and end (metabolic equivalents [METs]-minutes [min]/week).

SE:Stretching Exercise.

**Figure S55 Dose variation of AE to enhance chest expansion.**


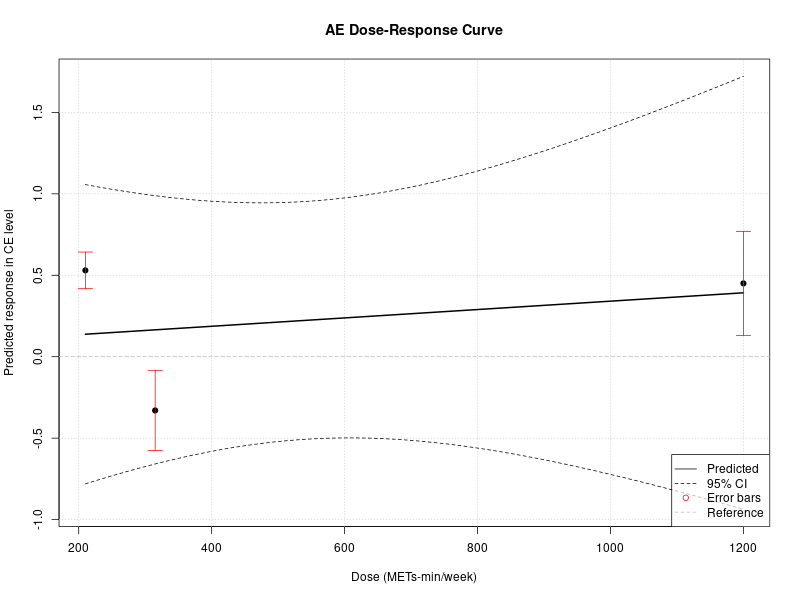


AE:Aerobic Exercises.

**Figure S56 Dose variation of AE+SE to enhance chest expansion.**


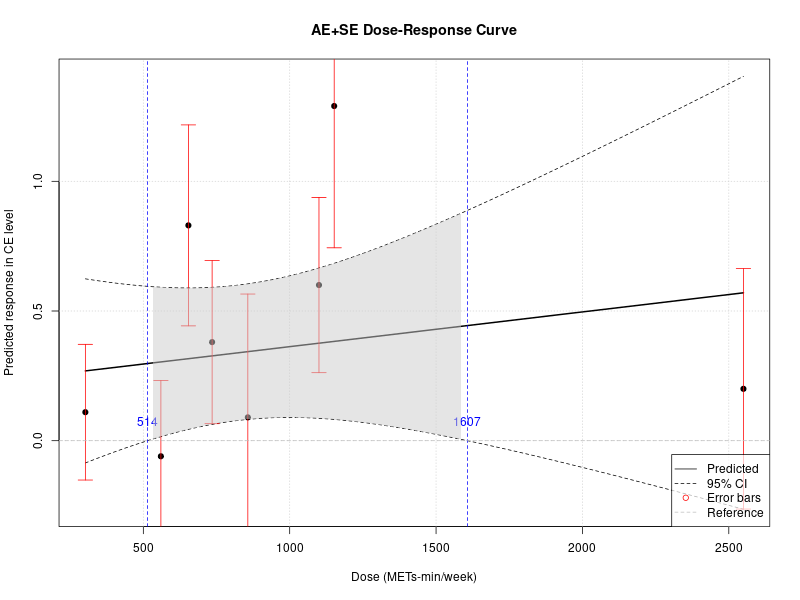


The nodes represent the original dataset. Shaded areas represent signifcant 95% credible intervals(CI). The blue dashed lines represent the beginning and end of the signifcant 95% CI, and the blue numbers represent the specifc dose values at the beginning and end (metabolic equivalents [METs]-minutes [min]/week).

AE:Aerobic Exercises; SE:Stretching Exercise.

**Figure S57 Dose variation of AE+SE+Supervise to enhance chest expansion.**

**
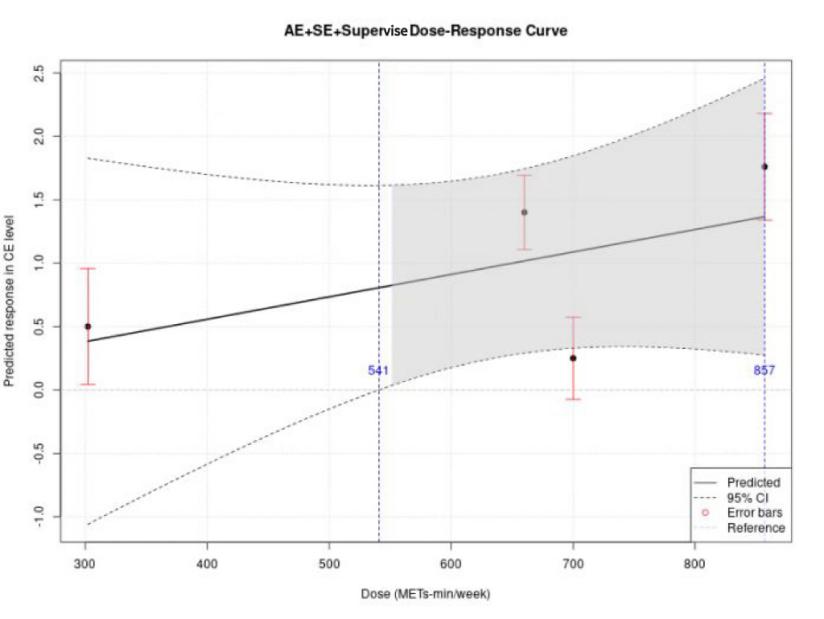
**

The nodes represent the original dataset. Shaded areas represent signifcant 95% credible intervals(CI). The blue dashed lines represent the beginning and end of the signifcant 95% CI, and the blue numbers represent the specifc dose values at the beginning and end (metabolic equivalents [METs]-minutes [min]/week).

AE:Aerobic Exercises; SE:Stretching Exercise.

**Figure S58 Dose variation of Pilates to enhance chest expansion.**


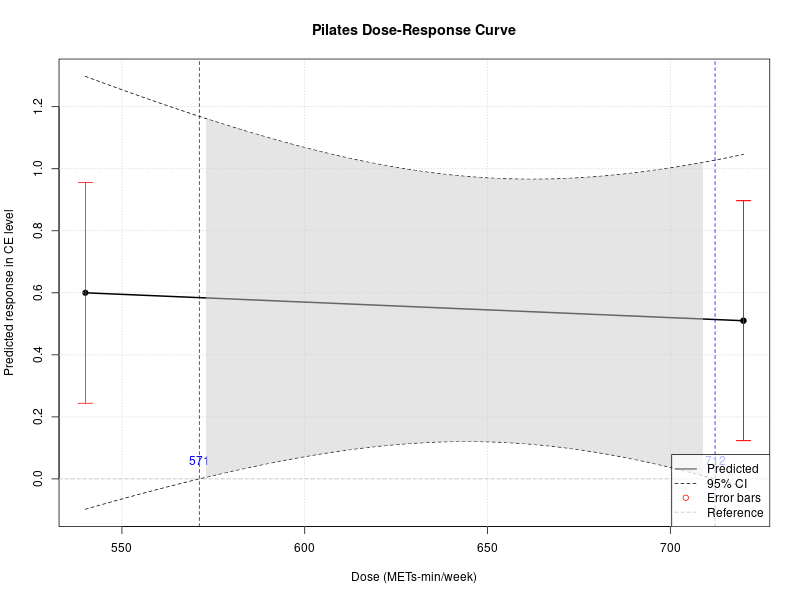


The nodes represent the original dataset. Shaded areas represent signifcant 95% credible intervals(CI). The blue dashed lines represent the beginning and end of the signifcant 95% CI, and the blue numbers represent the specifc dose values at the beginning and end (metabolic equivalents [METs]-minutes [min]/week).

**Figure S59 Dose variation of Qigong to enhance chest expansion.**


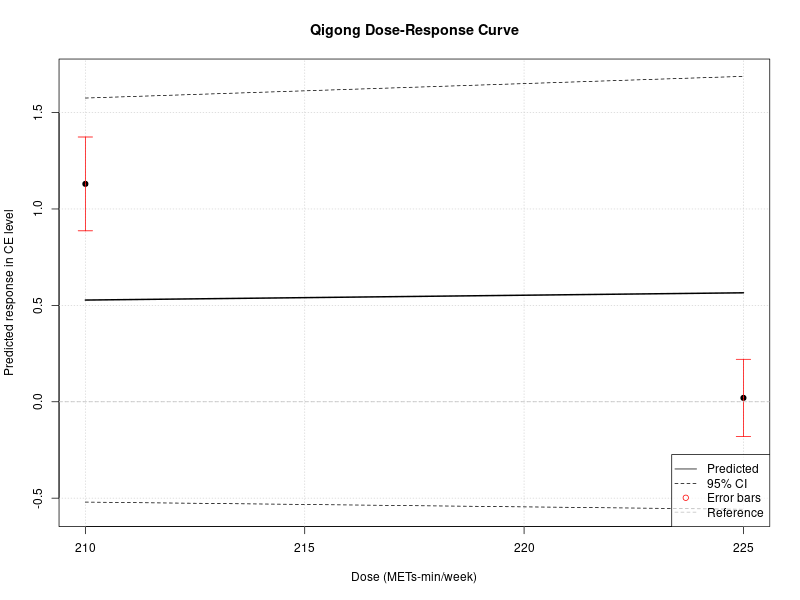


**Figure S60 Dose variation of SE to enhance chest expansion.**


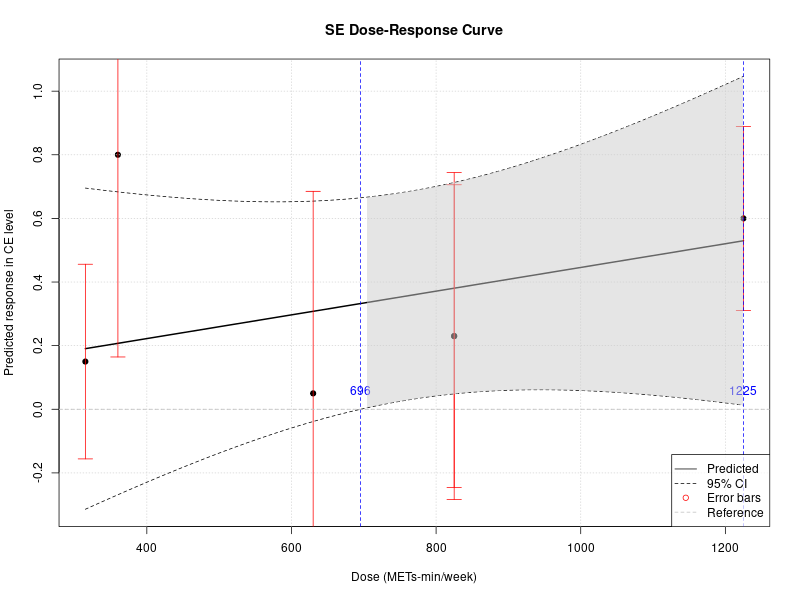


The nodes represent the original dataset. Shaded areas represent signifcant 95% credible intervals(CI). The blue dashed lines represent the beginning and end of the signifcant 95% CI, and the blue numbers represent the specifc dose values at the beginning and end (metabolic equivalents [METs]-minutes [min]/week).

SE:Stretching Exercise.
